# Binary combinatorial scanning reveals potent poly-alanine-substituted inhibitors of protein-protein interactions

**DOI:** 10.1038/s42004-022-00737-w

**Published:** 2022-10-14

**Authors:** Xiyun Ye, Yen-Chun Lee, Zachary P. Gates, Yingjie Ling, Jennifer C. Mortensen, Fan-Shen Yang, Yu-Shan Lin, Bradley L. Pentelute

**Affiliations:** 1 Department of Chemistry, Massachusetts Institute of Technology, 77 Massachusetts Avenue, Cambridge, MA 02139 USA; 2 Institute of Sustainability for Chemicals, Energy and Environment (ISCE2), Agency for Science, Technology and Research (A*STAR), 8A Biomedical Grove, Singapore, 138665 Singapore; 3 Department of Chemistry, Tufts University, 62 Talbot Avenue, Medford, MA 02155 USA; 4 Department of Chemistry and Frontier Research Center on Fundamental and Applied Sciences and Matters, National Tsing Hua University, 101, Sec. 2, Guang-Fu Road, Hsinchu, 300 Taiwan; 5 The Koch Institute for Integrative Cancer Research, Massachusetts Institute of Technology, 500 Main Street, Cambridge, MA 02142 USA; 6 Center for Environmental Health Sciences, Massachusetts Institute of Technology, 77 Massachusetts Avenue, Cambridge, MA 02139 USA; 7 Broad Institute of MIT and Harvard, 415 Main Street, Cambridge, MA 02142 USA; 8 Present Address: Department of Chemistry, National Cheng Kung University, No.1, University Road, Tainan City, 701 Taiwan; 9 Present Address: Disease Intervention Technology Laboratory (DITL), Agency for Science, Technology and Research (A*STAR), 61 Biopolis Drive, Singapore, 138673 Singapore

**Keywords:** Combinatorial libraries, Drug screening, Peptides

## Abstract

Establishing structure–activity relationships is crucial to understand and optimize the activity of peptide-based inhibitors of protein–protein interactions. Single alanine substitutions provide limited information on the residues that tolerate simultaneous modifications with retention of biological activity. To guide optimization of peptide binders, we use combinatorial peptide libraries of over 4,000 variants—in which each position is varied with either the wild-type residue or alanine—with a label-free affinity selection platform to study protein–ligand interactions. Applying this platform to a peptide binder to the oncogenic protein MDM2, several multi-alanine-substituted analogs with picomolar binding affinity were discovered. We reveal a non-additive substitution pattern in the selected sequences. The alanine substitution tolerances for peptide ligands of the 12ca5 antibody and 14-3-3 regulatory protein are also characterized, demonstrating the general applicability of this new platform. We envision that binary combinatorial alanine scanning will be a powerful tool for investigating structure–activity relationships.

## Abstract (web summary)

Alanine substitution in peptides is crucial for studying peptide-based inhibitors of protein–protein interactions, but limited information is obtained from single alanine substitution. Here, the authors develop a label-free combinatorial alanine affinity selection platform to establish multi-alanine mutational tolerance and provide structure-activity relationships.

## Introduction

Protein–protein interactions (PPIs) drive many aspects of biological function and are heavily involved in disease progression. The extensive (1000–5000 Å^2^), shallow and flat PPI interface challenges the development of PPI modulators using small molecules^1–3^. Peptides, on the other hand, can mimic the native binding epitope to recognize the PPI interface with high binding affinity and specificity. Recent advances in affinity selections and biological display methods have accelerated the generation of peptide-based PPI inhibitors^1,4^. To improve biophysical and pharmacological properties, iterative optimization is necessary. This process involves various structural modifications, for example, side chain modifications or macrocyclization to develop better analogs^5,6^. Modifying peptides while maintaining their binding affinity is crucial for hit-to-lead drug development, thus calling for a deep understanding of structure–activity relationships (SAR)^7^.

Alanine scanning informs SAR of peptides by systematically substituting each residue with alanine. This approach characterizes alanine tolerable residues and irreplaceable ‘hotspot’ residues essential for activity. Hotspots are identified by point alanine mutations that give rise to inactive mutants^8^. The alanine tolerable residues are often subjected to structure modifications without impairing the bioactivity. When multiple modifications happen simultaneously, non-additive combination effects emerge^9–13^, leading to unforeseen boosts or disruptions in activity. Complementary to single-point alanine scanning, shotgun alanine scanning is widely employed in protein mutagenesis to interrogate the pairwise and higher order combination effect of multi-point mutations^14–18^. As an example, the phage-displayed γ-receptor protein library was constructed by varying eleven residues to wild type (WT) or alanine^19,20^. The library was subjected to bioactivity assays to select for active strains, which presented a specific ratio of wild type to alanine at each residue. Pairwise analysis showed the frequency of most double alanine mutations followed a normal distribution when the two mutated residues were located in discontiguous regions^14,21^. However, for peptides an alanine mutation at one residue may affect the tolerance of an alanine mutation at a neighbouring residue. An analysis of the combined effects of multi-site modifications is thus critical at revealing the comprehensive peptide SAR landscape.

**Fig. 1:**
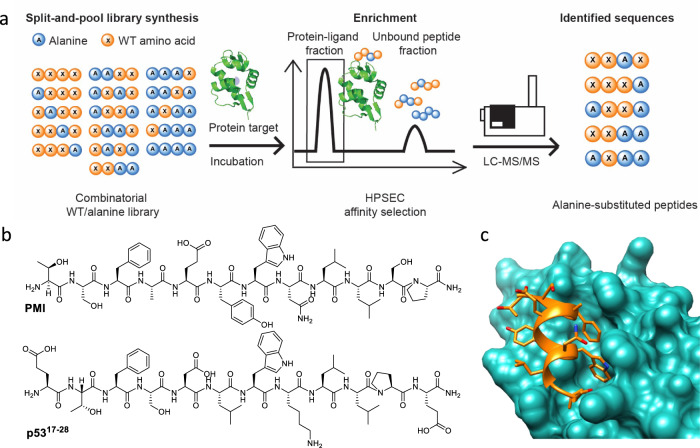
Combinatorial alanine scanning enables identification of alanine-containing peptide binders to proteins of interest. **a** A chemically synthesized combinatorial alanine library was generated by split-and-pool solid phase peptide synthesis. The library was cleaved, deprotected, solid phase extracted, and incubated with protein targets. The peptide–protein complex eluate was separated from unbound peptides by size-exclusion chromatography. Bound peptide binders were dissociated from the protein and then sequenced by Q-TOF liquid chromatography-tandem mass spectrometry (LC-MS/MS). **b** Chemical structure of p53^17–28^ peptide (ETFSDLWKLLPE-OH) and PMI (TSFAEYWNLLSP-NH_2_). **c** Crystal structure of the PMI–MDM2 complex (TSFAEYWNLLS-NH_2_, PDB entry 3LNZ)^23^.

We established a label-free combinatorial alanine library affinity selection (Fig. 1a) platform to rapidly identify multiple sites in peptide-based lead compounds that tolerate modification while maintaining bioactivity. Peptide libraries were synthesized by the split-and-pool solid-phase peptide synthesis (SPPS) method and enriched by affinity selection according to an established protocol^22^. The peptide library was incubated with the target protein and subjected to high-performance size-exclusion chromatography (HPSEC) to separate bound and unbound ligands. The bound variants were decoded by liquid chromatography-tandem mass spectrometry (LC-MS/MS)^23^. Synthetic peptide libraries avoid possible loss of sequences due to expression limitation or proteolysis characteristic of biological methods^4,24^. This in-solution affinity selection is a powerful alternative to on-bead screening and provides fine control over the selection conditions.

The strategy was applied to the PMI peptide inhibitor of oncoprotein mouse double minute 2 homolog (MDM2). The PMI sequence (TSFAEYWNLLSP-NH_2_) with high affinity (dissociation constant, *K*_D_ = 7.7 ± 4.5 nM) against MDM2 was discovered by phage display^25,26^ (Fig. 1b, c). MDM2 is an E3 ubiquitin ligase of the transcription factor p53 that responds to stress by promoting DNA repair, cell cycle arrest, senescence and apoptosis^27^. MDM2 recognizes the N-terminal p53 transactivation domain (p53^17–28^, see Fig. 1b for amino acid sequence) and promotes the ubiquitin-mediated p53 degradation. Disrupting p53–MDM2 binding is a strategy to restore p53 activity and promote apoptosis of cancer cells^28^. Aided by the combinatorial alanine scanning technique developed here, we identified several PMI analogs with simultaneous multi-alanine substitutions that maintained high affinity for MDM2. In some cases, we found the multi-alanine PMI variants lead to active cysteine (Cys)-substituted peptide macrocycles. To expand the application scope, we applied the strategy on peptide binders of antibody 12ca5 and the signalling protein 14-3-3σ, and identified their alanine tolerance.

## Results and discussion

The combinatorial alanine scanning platform was developed to identify alanine-containing peptide binders of the target protein (Fig. 1a). A PMI-derived combinatorial alanine library was prepared by split-and-pool SPPS. During synthesis, each position was evenly pooled to give either the wild-type amino acid or alanine, resulting in a library of 4,096 peptide variants. A C-terminal lysine is included to improve de novo LC-MS/MS sequencing. Prior to affinity selection, the library solution was incubated with MDM2 in Tris buffer (pH = 7.5) to reach equilibrium. The peptide–protein complexes were enriched by HPSEC where the early protein fraction eluted. Bound peptides were dissociated from the protein, sequenced by LC-MS/MS and analyzed with the PEAKS software suite^23^. Identified sequences were filtered based on the library design^23^. Quality control of the peptide library confirmed the recovery of sequences and even distribution prior to screening. To identify non-specific binders in the PMI-based library, we screened in parallel against the 12ca5 clone of antihemagglutinin antibody, for which no sequences were enriched.

### Single position SAR analysis

**Fig. 2:**
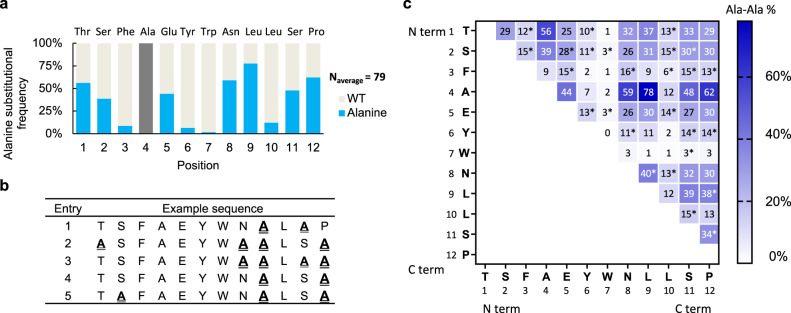
Combinatorial alanine scanning of peptide PMI combined with affinity selection mass spectrometry identifies multi-alanine substituted variants. **a** 1-D single position alanine substitution frequency. The alanine tolerance was indicated by the single position alanine substitution frequency on the *y*-axis. Residues displaying low alanine substitution frequency (Phe3, Tyr6, Trp7 and Leu10) correspond to the hotspot residues. The *x*-axis indexes the PMI sequence from the *N*- to the *C*-terminus. Ala4 is excluded. **b** A subset of identified sequences from the affinity selections. **c** The pairwise alanine tolerance was indicated by the 2-D pairwise alanine substitution frequency matrix. Each box represents the pairwise alanine substitution frequency (Ala-Ala%) of two residues, calculated as the ratio of number of observed simultaneous pairwise alanine substitutions to the total number of identified sequences, expressed as Ala-Ala% = [(*n*_Ala,Ala_)/*n*_total_] × 100%. At 95% confidence level, 25 out of 55 total possible pairwise substitution frequencies are non-additive of single alanine frequencies. Non-additive pairs are marked with asterisks (*). Ala4 is excluded from the analysis.

Positional alanine substitution frequency (Fig. 2a) can be used to differentiate binding hotspots from non-essential residues. Unique alanine mutant peptides were recovered from the affinity selection (Fig. 2b). For each position, the alanine frequency was determined by dividing the number of alanine mutations to the total number of identified sequences, averaging by three replicates (*N*_average_ = 79). Four positions Phe3, Tyr6, Trp7 and Leu10 are of low alanine frequency (Ala%<10%), consistent with hotspots determined by point alanine mutagenesis^26^.

To provide quantitative data on residue-specific contributions to binding affinity, positional alanine frequencies from the combinatorial scanning were converted to changes in Gibbs free binding energy (∆∆*G*_scanning_). This calculation assumes that the ratio of WT to Ala for each position (*n*_WT_*/n*_Ala_) approximates the ratio of equilibrium association constants *K*_A,WT_ to *K*_A,Ala_, such that ∆∆*G*_scanning_ is given by: *∆∆G*_Ala-WT_ = *RT* ln(*K*_A,WT_*/K*_A,Ala_) *= RT* ln(*n*_WT_*/n*_Ala_)^19^. By comparing the *∆∆G*_Ala-WT_ values calculated from combinatorial scanning reported here (∆∆*G*_scanning_) to the ∆∆*G*_binding_ values previously measured by point alanine mutagenesis^26^, we found the two correlated linearly (R^2^ = 0.88, Supplementary Fig. 1 and Supplementary Note 1). While ∆∆*G*_scanning_ for each position derived from the combinatorial scanning correlated well with the previously reported ∆∆*G*_binding_ values, the slope was found to be 0.36. This significant deviation from 1.0 suggests a numerical discrepancy between the ∆∆*G*_scanning_ and ∆∆*G*_binding_ and indicates that combinatorial alanine scanning tends to underestimate the effect of individual alanine point mutations. In particular, the ∆∆*G*_scanning_ values of the four hotspot residues (Phe3, Tyr6, Trp7 and Leu10) were all >1.0 kcal/mol, consistent with the conventional definition of hotspot^8^. Therefore, the combinatorial alanine scanning informs on SAR at the single position level.

### Pairwise SAR analysis reveals non-additive Ala-substituted pairs

To identify pairs of nonadditive Ala substitutions that might contribute to binding, a pairwise alanine substitution frequency matrix was generated^9,14^ (Fig. 2c). The pairwise alanine substitution frequency (Ala-Ala%) was computed by dividing the number of simultaneous pairwise alanine substitutions by the total number of identified sequences for each pair. Each box in the matrix presents a distinct pair of residues. For example, the residue pair (Thr1, Ser2) located in the first row and the second column of the matrix shows that 29% of the decoded sequences contained simultaneous (T1A, S2A) substitutions.

A statistical test showed that a number of Ala-Ala%’s were not a mere product of two single Ala%’s but statistically different from such a simple combination. A moderate non-additive combination effect was revealed by comparing the observed pairwise alanine substitution frequency (Ala-Ala%)_Observed_ to the product of single alanine frequencies (Ala-Ala%)_Additive_. The theoretical additive double-mutant frequencies were computed from a large number of sets (1,000 sets) of randomly-generated and independent alanine-substituted sequences, in which the randomization of each position is weighted by its positional alanine substitution frequency^14^. Each set contains hypothetical peptide sequences that follow the positional SAR. If a non-additive combination effect is present, (Ala-Ala%)_Observed_ would not be equal to (Ala-Ala%)_Additive_. To assess the statistical significance of the deviation, we compared the observed (Ala-Ala%)_Observed_ to the theoretical (Ala-Ala%)_Additive_. The difference (Ala-Ala%)_Observed_–(Ala-Ala%)_Additive_ was compared to the standard deviation (σ) of the theoretical additive values calculated from the random sets of sequences and assessed by the z-test (Supplementary Fig. 2). To normalize for randomness, the statistical test was averaged by three replicate selections.

At 95% confidence (|z score | >1.96σ), 25 out of 55 (44%) pairwise alanine substitution probabilities are statistically distinct from a simple combination of the corresponding two single alanine substitution probabilities (marked with asterisks in Fig. 2c). Among these 25 non-additive pairs, 23 pairs show positive non-additivity, and 2 pairs show negative non-additivity (Supplementary Fig. 2).

### Pairwise SAR validation

**Fig. 3:**
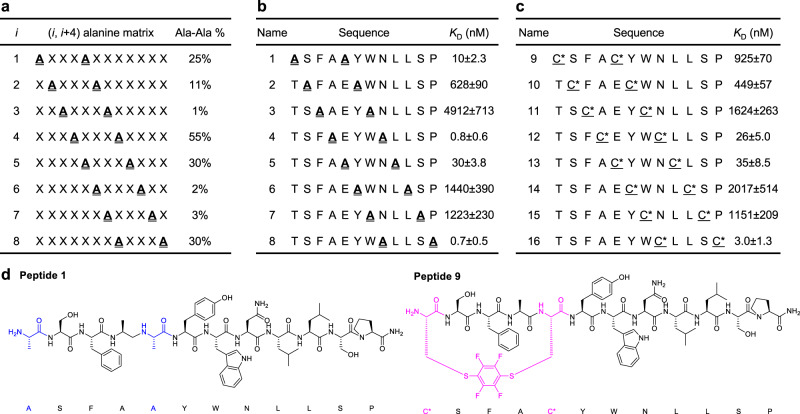
High-frequency alanine pairs observed in the affinity selection show high tolerance for double mutations. **a** (*i, i* + 4) pairwise alanine substitution frequency extracted from Fig. 2c. **b** Binding affinity of (*i, i* + 4) pairwise alanine-substituted peptides. **c** Binding affinity of (*i, i* + 4) perfluoroaryl stapled peptides. The three most potent stapled peptides correlate with the three highest frequency pairwise alanine substitutions. Therefore, the (*i, i* + 4) pairwise alanine substitution frequencies accurately indicate retention of high affinity in peptide binders substituted at the corresponding positions. Binding dissociation constants (*K*_D_) were determined by a competition assay using BLI. **d** Representative chemical structures of an (*i, i* + 4) pairwise alanine-substituted peptide (**1**) and an (*i, i* + 4) perfluoroaryl-stapled peptide (**9**).

We envisioned the positions displaying high pairwise substitution frequencies in our combinatorial alanine library (Fig. 3a, extracted from Figs. 2c and 3d) would tolerate double mutations. To validate the correlation between pairwise substitution frequencies and binding affinity of double-mutants, a series of (*i, i* + 4) pairwise alanine substituted peptides were prepared by automated fast-flow peptide synthesis^29^. (*i, i* + 4) Positions are chosen for subsequent macrocyclization^30^. The binding constant was determined by performing a competition assay by biolayer interferometry (BLI). Replacing the above average-frequency (*i, i* + 4) alanine-substituted pairs to alanine has minor impact on the binding affinity (Fig. 3b; PMI, *K*_D_ = 7.7 ± 4.5 nM).

Pairwise alanine-tolerated positions prompt sites amenable to further modifications, i.e., stapling. Side chain stapling is used for reinforcing helical structure and improving peptide stability toward proteases^31–33^. To leverage the pharmacological properties of the identified MDM2 binders, a hexafluorobenzene-mediated cysteine arylation reaction was employed to generate (*i, i* + 4) stapled PMI analogs (peptides **9-16**)^34^. BLI competition assays showed that the three highest frequency (*i, i* + 4) pairwise alanine substitutions (peptides **4**, **5** and **8** in Fig. 3c) gave rise to the three highest affinity (*i, i* + 4) perfluoroaryl-stapled peptide binders at the corresponding positions. Peptides **12** and **13** showed slightly attenuated binding, and **16** exhibited a comparable binding to the parent PMI inhibitor (*K*_D_ = 7.7 ± 4.5 nM). This result indicates that peptides stapled at high (*i, i* + 4) alanine frequency positions can maintain low nanomolar binding affinity to MDM2.

### Multiple alanine-substituted peptides retain potent binding affinity

**Fig. 4:**
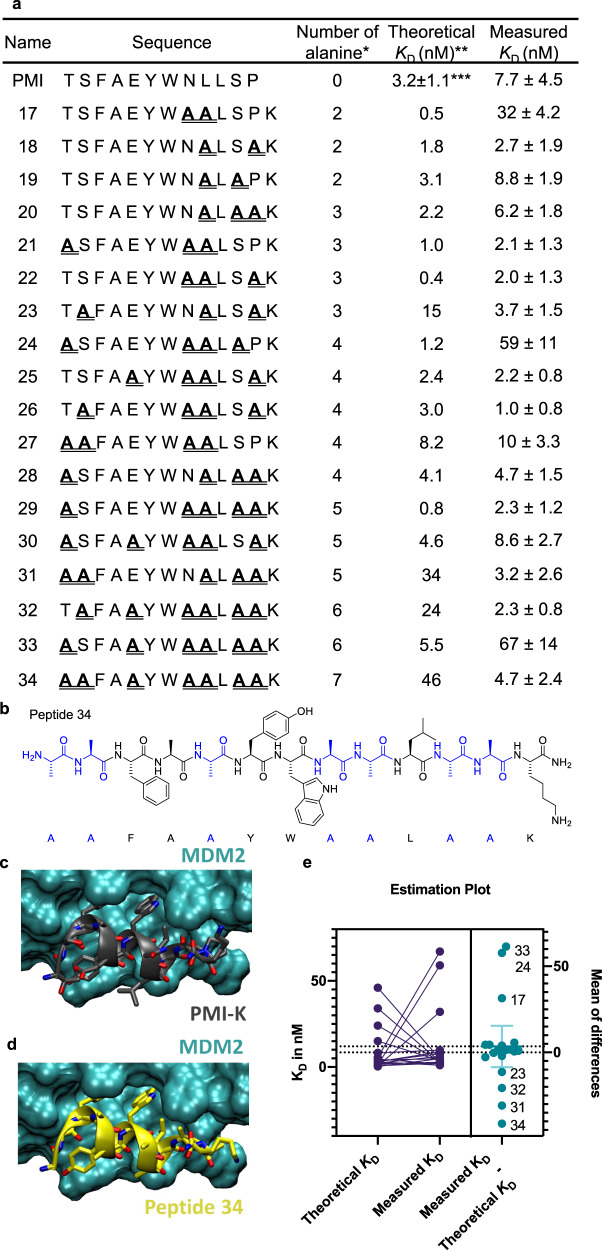
Multiple alanine-substituted peptides exhibit nanomolar binding affinity. **a** A subset of alanine-substituted peptides were identified and resynthesized and validated after increasing the selection stringency. Dissociation constants (*K*_D_) were determined by a competition assay using BLI. *Ala4 is not considered. **Theoretical *K*_D_ (*K*_D_’) was calculated by adding the residue-specific energy contributions of individual alanine mutations (detailed calculation described in Supplementary Fig. 4). ***The reported *K*_D_ of PMI^25^. **b** A 13-mer peptide **34** with seven alanine substitutions was identified (*K*_D_ = 4.7 ± 2.5 nM). Molecular docking results of (**c**) PMI-K (gray) and (**d**) peptide **34** (yellow) bound to MDM2 (cyan). **e** Paired t-test of theoretical *K*_D_’ and measured *K*_D_. The left plot shows the *K*_D_ and *K*_D_’ values. The right plot denotes the mean of difference calculated from the paired t-test. Error bar denotes 95% confidence level.

By increasing the selection stringency, a small number of potent multiple alanine-substituted binders were identified (Fig. 4a and Supplementary Method 2). Using a smaller amount of the library increased the ligand–protein binding threshold, and consequently reduced the number of identified peptides. We used the same algorithm shown in Supplementary Figs. 2 and 3 to generate a pairwise matrix of the multiple alanine-substituted peptides (Supplementary Fig. 3). As shown by the matrix, high alanine percentage (Ala% > 45%) was observed with the substitution pairs (T1, L9), (N8, L9), (L9, S11), and (L9, P12). Peptides with different alanine content were individually synthesized, and their binding affinity was validated by a competition assay using BLI. Several peptides exhibited low nanomolar binding affinity. Triple alanine sequences frequently occurred in the most stringent selection (Supplementary Fig. 4). They retained or even enhanced the protein–ligand binding interaction (Fig. 4a).

To study the non-additive effect of multi-alanine substituted variants, we calculated the theoretical binding affinity (*K*_D_’) by adding ∆∆*G*_binding_, the residue-specific energy contribution of alanine mutations, of all substituted positions for a given peptide (Supplementary Fig. 5) and using the reported affinity of PMI (*K*_D_ = 3.2 ± 1.1 nM^26^). The theoretical binding affinity *K*_D_’ falls under the additivity assumption. Then we compared the theoretical *K*_D_’ to the measured *K*_D_ of alanine substituted variants by running a paired t-test (Fig. 4c). At 95% confidence, 7 out of 19 (37%) variants showed significant difference in the theoretical *K*_D_’ and measured *K*_D_. Three variants showed negative non-additivity while four variants showed positive non-additivity.

To analyze the non-additivity observed in the multi-alanine variants with a complex substitution pattern, we analyzed the 7 variants. Comparing the theoretical and measured binding affinities, peptides **17**, **22** and **24** displayed ten-fold higher experimental *K*_D_ values, while peptides **31**, **32**, **33** and **34** exhibited ten-fold lower experimental *K*_D_ values relative to the calculated *K*_D_’ (Fig. 4a). Within the three negative non-additive variants, they all share N8A and L9A substitutions. The pairwise matrix indicated that the (N8, L9) pair is negatively non-additive, therefore, this pair may contribute to the non-additive decrease in binding affinity of peptide **17**, even though it only contains a smaller number of alanine substitutions. On the other hand, the four positive non-additive variants all contain the (S11, P12) Ala substitution pair, which may contribute to the non-additive increase in the binding affinity of peptides **31**, **32**, and **34**. The theoretical *K*_D_’ given by simple addition of positional ∆∆*G*_binding_ can thus deviate significantly from the measured *K*_D_, and may not accurately predict high-affinity multi-alanine substituted PMI variants.

Noticeably, peptide **34** has all of its non-hotspot residues substituted to alanine, while still maintaining a binding affinity comparable to PMI (Fig. 4b). Single alanine mutagenesis predicts the *K*_D_ of peptide **34** to be 10-fold weaker than PMI, while peptide **34**, shows a *K*_D_ of 4.7 ± 2.4 nM. Rigid molecular docking of peptide **34** to MDM2 using AutoDock Vina (except the side chains of the peptide, which were kept flexible) also shows that the calculated binding affinity was improved upon Ala substitutions at all seven non-hotspot residues, with a ∆∆*G* of –3.4 kcal/mol for PMI and –2.5 kcal/mol for PMI-Lys (Fig. 4c, d, Supplementary Table 1 and Supplementary Method 3). Even though it is generally accepted that multiple alanine substitutions are detrimental to binding affinity^35^, in our hands multi-alanine-substituted MDM2 binding peptides could be identified in the enriched sequences.

### Multi-alanine substituted variants tolerate stapling

**Fig. 5:**
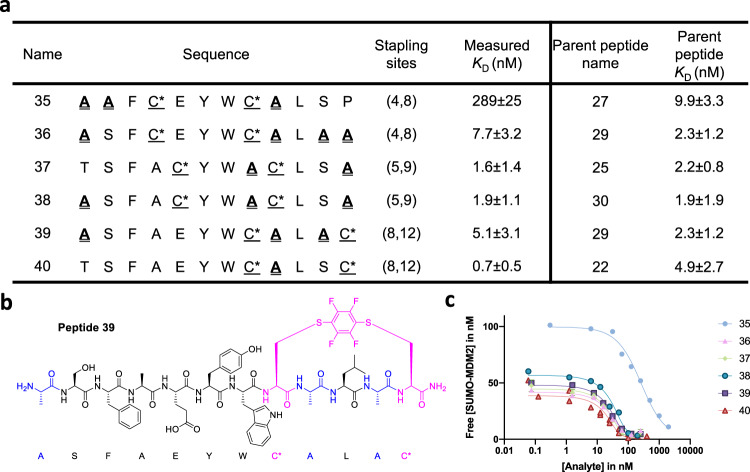
Perfluoroaryl-stapled and alanine-substituted peptides demonstrate high-affinity binding. **a** Perfluoroaryl-stapled and alanine-substituted peptides **35–40** with low nanomolar binding affinity were generated at tolerable (*i, i* + 4) stapling sites (Fig. 3c). **b** Structure of peptide **39**, alanine is colored in purple and stapling sites are colored in blue. **c** Example competition binding curves of peptides. C* = cysteine stapled with hexafluorobenzene.

To test the tolerance of multi-alanine-substituted peptides for subsequent modifications, we applied the (*i*, *i* + 4) perfluoroaryl stapling strategy to alanine-substituted sites of peptides **22**, **25**, **27**, **29** and **30** (Fig. 5)^34^. Three stapling positions, (4, 8), (5, 9) and (8, 12), were chosen based on the tolerance for pairwise substitutions. The highest affinity (*K*_D_ = 0.7 ± 0.5 nM) was found with the (8,12) perfluoroaryl-stapled peptide **40**. Except stapled peptide **35**, stapled peptides **36**, **37**, **38** and **39** showed comparable binding affinity to PMI.

To assess the stability of stapled peptides, we performed a serum stability assay (Supplementary Fig. 6)^31,36,37^. Three stapled peptides were selected for assessment. Compared with the unmodified PMI-NH_2_ peptide (*t*_1/2_ = 2.2 min), linear peptide **32** (*t*_1/2_ = 3.9 min) and **34** (*t*_1/2_ = 5.1 min), the half-life of stapled peptides **36**, **38** and **39** increased to 26, 27 and 25 min, respectively (Supplementary Fig. 6).

**Fig. 6:**
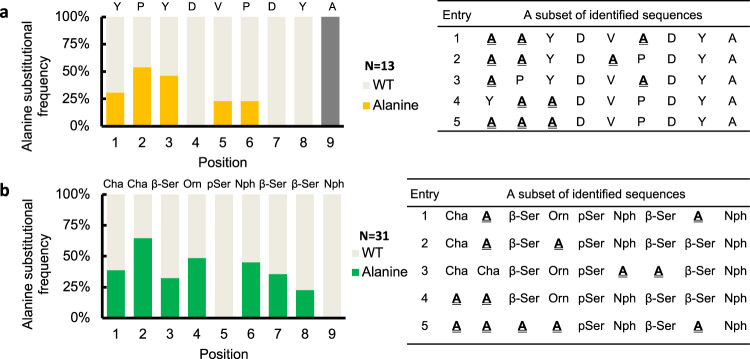
Combinatorial alanine scanning is applicable to peptide binders for antibody 12ca5 and regulatory protein ﻿14-3-3σ. **a** Alanine substitution frequency at each position of the 12ca5 binder HA epitope (sequence: YPYDVPDYA). **b** Alanine substitution frequency at each position of the protein 14-3-3σ binder 14-3-3.6 construct (sequence: Cha-Cha-β-Ser-Orn-pSer-Nph-β-Ser-β-Ser-Nph); abbreviations: Cha cyclohexyl alanine, β-Ser beta-homoserine, Orn ornithine, pSer phosphoserine, Nph 4-nitro phenylalanine. Wild-type alanine is excluded from the bar graph.

To demonstrate a more general applicability of this platform, in addition to MDM2, the combinatorial alanine scanning was also applied to peptide binders of antibody 12ca5 and the signalling protein 14-3-3σ. The epitope used for 12ca5 has the sequence YPYDVPDYA; the previously characterized protein 14-3-3σ binder 14-3-3.6 was used for 14-3-3σ and its sequence can be found in Fig. 6b. A beta-alanine spacer was used between the library construct and the C-terminal lysine. The combinatorial alanine scanning of YPYDVPDYA (*K*_D_ = 4 nM) showed no alanine substitution at Asp4, Asp7 and Tyr8, which were previously reported as the hotspot residues of the epitope (Fig. 6a)^26^. The combinatorial alanine scanning of peptide 14-3-3.6 (*K*_D_ = 3 nM) showed no alanine replacement at phosphoserine 5 and nitro-phenylalanine 9 (Fig. 6b), consistent with the close interactions these two residues have with 14-3-3σ, as seen in the co-crystal structure^38^. Remarkably, multiple alanine-substituted binders were identified under the most stringent condition as shown in the subset of sequences (Fig. 6).

## Conclusions

We developed a label-free combinatorial alanine affinity selection platform to establish mutational tolerance, inform structure–activity relationships, and facilitate the optimization of peptide-based PPI modulators. Using various statistical analyses and peptide modifications, several sequence activity relationships were inferred. At the single substitution level, the determined alanine substitution frequencies differentiated between hotspot and non-hotspot residues of the MDM2 peptide binder PMI. At the double substitution level, statistical analyses of the pairwise alanine substitution frequencies identified a moderate but significant non-additive combination effect. We can identify suitable sites that can tolerate simultaneous mutations and stapling based on high pairwise alanine substitution percentages, as validated by binding experiments with (*i, i* + 4) doubly-alanine-substituted peptides and (*i, i* + 4) stapled peptides.

A handful of multiple alanine-substituted binders were found to maintain their binding affinity. This finding occurred when alanine was combinatorically introduced to peptide libraries that underwent affinity selections. The multi-substituted peptides can further tolerate cysteine-based stapling and still retain nanomolar binding affinity, despite the fact that stapling can potentially change the binding affinity of peptides^30^. This study shows the potential of the combinatorial alanine scanning platform to identify multiple positions within a sequence that can simultaneously accommodate further modifications. These high affinity stapled peptides may further be modified at the remaining alanine residues and provide a starting point in the development of the next generation of therapeutics targeting the oncogenic p53–MDM2 interaction.

Our study demonstrates a broad alanine tolerance landscape of peptide-based binders, provides insights into the non-additivity of combinatorial substitutions, and identifies multi-alanine-substituted MDM2-binding peptides. These variants can guide peptide ligand optimization through chemical stapling and improve the throughput of hit-to-lead optimization processes. Albeit these peptide variants are synthetically accessible, their sequencing may be limited by solubility and ionization efficiency, to the extent that library quality control studies are necessary. We envision that combinatorial alanine scanning can be a valuable tool for determining the feasible structural modifications of potentially therapeutic peptide leads toward enhancing their biophysical properties, such as binding affinity, serum stability, rigidity, and lipophilicity.

## Methods

### Materials

Unless otherwise noted, all commercial chemicals and reagents were used without further purification. H-Rink Amide-ChemMatrix resin was purchased from PCAS BioMatrix Inc. TentaGel S NH_2_ (130 μm, 0.2-0.35 mmol/g) was purchased from Rapp Polymere GmbH (Tuebingen, Germany). *Linkers*: 4-[(*R*,*S*)-α-[1-(9*H*-Fluoren-9-yl)-methoxyformamido]-2,4-dimethoxybenzyl]-phenoxyacetic acid (Fmoc-Rink amide linker) was purchased from Chem-Impex International (Wood Dale, IL). *Amino acids*: Fmoc-Ala-OH, Fmoc-β-Ala-OH, Fmoc-Asn(Trt)-OH, Fmoc-Asp(*t*Bu)-OH, Fmoc-Cys(Trt)-OH, Fmoc-Glu(*t*Bu)-OH, Fmoc-Leu-OH, Fmoc-Lys(Boc)-OH, Fmoc-Phe-OH, Fmoc-Pro-OH, Fmoc-Ser(*t*Bu)-OH, Fmoc-Thr(*t*Bu)-OH, Fmoc-Trp(Boc)-OH, Fmoc-Tyr(*t*Bu)-OH, and Fmoc-Val-OH were purchased from Novabiochem (Billerica, MA). *Noncanonical amino acids*: Fmoc-β-cyclohexyl-*L*-alanine (Cha), Fmoc-*O*-tert-butyl-*L*-β-homoserine (β-Ser), Fmoc-*L*-Orn (Boc)-OH (Orn), Fmoc-Ser(PO(OBzl)OH)-OH (pSer) and Fmoc-4-nitro-*L*-phenylalanine (Nph) were purchased from Chem-Impex International (Wood Dale, IL). *N*-α-Fmoc-*O*-benzyl-L-phosphoserine was purchased from MilliporeSigma (St. Louis, MO). *Reagents used in solid phase peptide synthesis*: Piperidine (ReagentPlus; 99%), formic acid (≥98%) were purchased from Sigma-Aldrich (St. Louis, MO). Diisopropylethylamine (DIEA; biotech. grade; 99.5%) was purchased from MilliporeSigma and purified by a Seca Solvent Purification system from Pure Process Technology (Nashua, NH). *Reagents for peptide cleavage*: Trifluoroacetic acid (TFA; for HPLC, ≥ 99%), triisopropylsilane (TIPS; 98%), 1,2-ethanedithiol (EDT; ≥ 98%), phenol (≥99%), and thioanisole (≥99%) were purchased from Sigma-Aldrich (St. Louis, MO). *Reagents used in peptide post-synthesis modifications*: hexafluorobenzene (≥99%) was purchased from TCI (Portland, OR), 2-Amino-2-(hydroxymethyl)propane-1,3-diol Tris (base) was purchased from J.T. Baker (Center Valley, PA). Acetic anhydride (≥98%) was purchased from Sigma-Aldrich (St. Louis, MO). *Solvents*: Deionized water was obtained from a Milli-Q Reference water purification system (Millipore). *N*,*N*-Dimethylformamide (DMF; peptide synthesis grade), dichloromethane (DCM), diethyl ether (Et_2_O), and acetonitrile (MeCN; HPLC grade) were purchased from VWR International (Philadelphia, PA). *Proteins*: Mouse anti-hemagglutinin (HA) monoclonal antibody clone 12ca5 (anti-HA mAb 12ca5) was purchased from Columbia Biosciences (Frederick, MD). Bovine serum albumin (BSA; RIA grade) was purchased from Amresco (Solon, OH). 14-3-3sigma was kindly provided by Professor Christian Ottmann^38^. *Buffers and other reagents*: Phosphate-buffered saline 10x (10x PBS, Molecular biology grade) was purchased from Corning (Manassas, VA). Tween 20 (reagent grade) was purchased from Amresco (Solon, OH). Trichloroacetic acid (ACS grade, TCA) was purchased from MilliporeSigma (St. Louis, MO). Bradford assay kit purchased from Sigma-Aldrich (St. Louis, MO). Human serum was purchased from MilliporeSigma (St. Louis, MO).

### SUMO-^25-109^MDM2 expression

The plasmid construct and *E. coli* expression of SUMO-^25-109^MDM2 were achieved^39^. In brief, the ^25-109^MDM2 gene was purchased from Addgene (pGEX-4T MDM2 wild type (WT), 16237). The SUMO tag was incorporated using the Champion™ pET SUMO Expression System (Invitrogen, CA). SUMO-^25-109^MDM2 was expressed in Rosetta (DE3) pLysS cells. The bacteria were inoculated and cultured for 6 hours to reach OD600 = 0.5 under ambient temperature, induced with 0.4 mM IPTG for 4 hours, and pelleted. Approximately 1 L broth produced 10 g cell pellet, which was resuspended in 30 mL of 50 mM Tris-HCl, 150 mM NaCl, pH 7.5 buffer containing 40 mg lysozyme, 1 mg Roche DNAase I, and one tablet of Roche protease inhibitor cocktail, and then sonicated for three times for 20 s. The suspension was then centrifuged at 30,000 rcf for 30 min to clarify the lysate. The supernatant was loaded onto a 5 mL HisTrap FF crude Ni-NTA columns (GE Healthcare, UK), and washed sequentially with 30 mL of 20 mM Tris-HCl, 150 mM NaCl, pH 8.5 and then 30 mL 20 mM Tris-HCl 150 mM NaCl, 80 mM imidazole pH 8.5. The protein was eluted with 10 mL 20 mM Tris-HCl, 500 mM imidazole, 500 mM NaCl, pH 8.5. The eluted protein was buffer exchanged into 20 mM Tris-HCl, 50 mM NaCl, pH 8.5 using a HiPrep 26/10 Desalting column (GE Healthcare, UK). The resulting protein solution was purified the same day using a 5 mL HiTrap Q HP (GE Healthcare, UK) anion exchange columns with a linear NaCl gradient (B% graded from 5% to 25%, B = 20 mM Tris-HCl, 500 mM NaCl, pH 8.5). Fractions containing pure SUMO-^25-109^MDM2, as determined by SDS-PAGE, were collected and concentrated to 0.5 mg/mL using a 3,000 Da nominal molecular weight limit Amicon Ultra-15 Centrifuge Filter Unit (EMD Millipore), and immediately flash frozen and stored at −80 °C.

### Peptide synthesis

H-Rink Amide-ChemMatrix resin (200 mg, 0.49 mmol/g, 0.10 mmol) was used to prepare peptides. Peptides containing noncanonical amino acids were prepared by manual solid-phase peptide synthesis. Peptides without noncanonical amino acids were prepared by fully automated SPPS^29^. Upon completion, resins were washed with DCM (3x) and dried under reduced pressure.

### Solid-phase peptide synthesis

200 mg of H-Rink Amide-ChemMatrix resin (0.10 mmol) was placed in a 5 mL Torviq fritted syringe. After swelling in DMF for 10 min, the resin was then washed with DMF (3x). Each Fmoc-protected amino acid (0.50 mmol, 5.0 eq) was dissolved in 0.38 M HATU solution (DMF as solvent, 1.3 mL, 0.50 mmol). Immediately before the coupling, DIEA (130 μL, 0.72 mmol, 6.9 eq) was added to the mixture. The resulting mixture was sonicated briefly, and then transferred to the fritted syringe containing the resin. Coupling was performed for 20 min. The resin was washed with DMF (3x). Fmoc deprotection was done by treating the resin with 20% piperidine in DMF (3 mL) for 5 min and this step was repeated twice. The resulting resin was then washed with DMF (3x). The synthetic cycle was repeated to completion of the peptide sequence.

#### Cleavage of peptides without cysteine

Synthesized peptide was cleaved from resin and globally deprotected by treating the peptidyl resin with a cleavage cocktail containing 94% TFA, 2.5% EDT, 2.5% water, and 1.0% TIPS (*v/v*), for 1 h at ambient temperature. TFA was removed under a gentle stream of nitrogen gas, and crude peptide was precipitated by the addition of cold Et_2_O (−80 °C). After centrifugation at 3220 rcf for 3 min, supernatant was removed, and precipitated peptide was triturated three times with cold Et_2_O. The resulting material was dissolved in 50% MeCN in water with 0.1% TFA, and lyophilized.

#### Cleavage of cysteine-containing peptides

Synthesized peptide was cleaved from resin and globally deprotected by treating the peptidyl resin with reagent K containing 82.5% TFA, 5.0% phenol, 5.0% water, 5.0% thioanisole, and 2.5% EDT (*v/v*), for 1 h at ambient temperature. TFA was removed under a gentle stream of nitrogen gas, and crude peptide was precipitated by the addition of cold Et_2_O (−80 °C). After centrifugation at 3220 rcf for 3 min, supernatant was removed, and precipitated peptide was triturated three times with cold Et_2_O. The resulting material was dissolved in 50% MeCN in water (*v/v*) with 0.1% TFA, and lyophilized.

### Purification of crude peptide

Crude peptides were purified by a Biotage Selekt flash purification system. Water with 0.1% TFA (solvent A) and MeCN with 0.1% TFA (solvent B) were utilized as mobile phases for purifications. The crude peptide was dissolved in minimal amount of 10% MeCN in water with 0.1% TFA, and then loaded onto a 10 g Biotage SNAP Bio C18 20 μm column. The purification was performed using a gradient as following: 10% B for 3 column volume (CV), linear ramp from 10% B to 70% B for 30 CV, 25 mL/min flow rate.

### Peptide acetylation

A 100 mg portion of peptidyl resin was placed into a 5 mL Torviq fritted syringe and subsequently swelled in DMF. After removing DMF, a solution of Ac_2_O, DIEA, and DMF (2 mL, 85:315:1600, *v/v*) was added to the peptidyl resin. The resulting mixture was occasionally agitated for 45 min. After draining the solution, the remaining resin was washed with DMF (3x), DCM (3x) and dried under reduced pressure.

### Peptide stapling

Crude cysteine-containing peptide (~14 mg, 0.010 mmol) was placed in a falcon tube, followed by the addition of DMF (1 mL). 200 mM hexafluorobenzene (DMF as solvent, 2.5 mL) and 150 mM Tris base (DMF as solvent, 1.5 mL) were added to the falcon tube. The tube was sealed and mixed on a nutating mixer for 4 h. The mixture was quenched by adding water with 0.1% TFA (20 mL, *v/v*), and then purified by a Biotage Selekt flash purification system with the gradient: 25% B for 3 CV, linear ramp from 25% B to 75% B for 30 CV; 25 mL/min flow rate.

### LC-MS characterization

LC-MS characterizations were carried out using an Agilent 6520 quadrupole time-of-flight LC-MS or an Agilent 6550 quadrupole time-of-flight LC-MS. The Agilent 6520 ESI-QTOF mass spectrometer was placed in-line with an Agilent 1290 Infinity liquid chromatography system, whereas the Agilent 6550 ESI-QTOF mass spectrometer was placed in-line with an Agilent 1290 Infinity LC system. Total ion current (TIC) chromatograms were plotted. Mass spectra were integrated over the principal TIC peaks. High-performance liquid chromatography was done by following methods: (solvent A: water with 0.1% formic acid; solvent B: MeCN with 0.1% formic acid). **Method A:** Column: Phenomenex Luna C18(2) column (0.5 ×150 mm, 3 μm particle size, 100 Å pore size). Gradient: 1% B (A-B min), linearly ramp from 1% B to 61% B (B-C min), 61% B to 95% B (C-D min). Flow rate is 50 μL/min. MS acquisition is from 4 to 14 min. **Method B:** Column: Agilent Zorbax 300SB C18 column (2.1 ×150 mm, 5 μm particle size, 300 Å pore size). Gradient: 1% B (0-2 min), linearly ramp from 1% B to 61% B (2-11 min), 61% B to 95% B (11-12 min). Flow rate is 0.6 mL/min. MS acquisition is from 4 to 14 min. **Method C:** Column: Phenomenex Jupiter C4 column (1.0 ×150 mm, 5 μm particle size, 300 Å pore size). Gradient: 1% B (0-2 min), linearly ramp from 1% B to 91% B (2-18 min), 61% B to 91% B (18-21 min). Flow rate is 100 μL/min. MS acquisition is from 4 to 18 min. **Method D:** Column: Phenomenex Jupiter C4 column (1.0 ×150 mm, 5 μm particle size, 300 Å pore size). Gradient: 1% B (0-2 min), linearly ramp from 1% B to 61% B (2-12 min), 61% B to 95% B (11-16 min). Flow rate is 100 μL/min. MS acquisition is from 4 to 12 min. **Method E:** Column: Agilent Zorbax 300SB C3 column (2.1 ×150 mm, 5 μm particle size, 300 Å pore size). Gradient: 1% B (0-2 min), linearly ramp from 1% B to 91% B (2-12 min), 91% B to 91% B (12-13 min). Flow rate is 500 μL/min. MS acquisition is from 4 to 12 min. LC-MS characterization data are shown in Supplementary Note 2.

### Representative protocol for split-and-pool solid phase peptide synthesis

#### Coupling

400 mg of 130 μm TentaGel resin (0.26 mmol/g, 0.10 mmol, 782770 beads/g, 3.13 ×10^5^ beads) was placed in a 10 mL polypropylene syringe containing a porous polypropylene disc (Torviq). After swelling in DMF for 10 min, the resin was washed with DMF (3x). Fmoc-Rink amide linker (560 mg, 1.0 mmol, 10.0 equiv) was dissolved in 0.38 M HATU solution in DMF (2.4 mL, 0.49 mmol, 9.0 equiv), activated with DIEA (544 μL, 1.56 mmol, 30.0 equiv). The mixture was sonicated briefly, transferred to the fritted syringe containing the resin, and allowed to stir for 20 minutes. After the coupling solution was drained, and the resin was washed with DMF (3x). Fmoc protection group was achieved by soaking the resin with 20% piperidine in DMF (6 mL) for 5 min for twice, and then washed with DMF (3x). A sequential Fmoc-Lys(Boc)-OH coupling, Fmoc removal and DMF washes were performed in the same manner.

#### Portioning

The peptidyl resin was suspended in DMF (6 mL), homogenized, then evenly divided among two 10 mL Torviq fritted syringes. After draining DMF from the syringes, the couplings were carried out as follows: Fmoc-protected WT amino acid and Fmoc-Ala-OH (0.26 mmol) were separately dissolved in 0.38 M HATU (0.66 mL, 0.26 mmol), activated with DIEA (136 μL, 0.36 mmol). Each of the activated amino acids was added to the individual resin-containing syringes (~200 mg, 0.052 mmol). After coupling for 20 min, the coupling solution was removed, and the resin was washed with DMF (3x). Then, all portions of peptidyl resin were combined and washed with DMF. Fmoc deprotection was achieved by treating the resin with 20% piperidine in DMF (6 mL, 5 min batch treatment), and this step was repeated twice. The resulting resin was then washed with DMF (3x). Twelve cycles of split-and-pool synthesis were performed using this procedure.

#### Cleavage from resin and global side chain deprotection

The library was cleaved from resin and globally deprotected by treating the peptidyl resin with a cleavage cocktail containing 94% TFA, 2.5% EDT, 2.5% water, and 1.0% TIPS (v/v), for 1 h at ambient temperature. TFA was removed under a gentle stream of nitrogen gas, and the crude peptide was precipitated using cold Et_2_O (−80 °C). After centrifugation at 3220 rcf for 3 min, the supernatant was removed, and the precipitated peptide was triturated three times with cold Et_2_O. The resulting material was dissolved in 50% MeCN in water with 0.1% TFA, and lyophilized.

#### Solid phase extraction

The library (60 mg) was dissolved in 6.0 mL of 5% MeCN in water with 0.1% TFA. 6 mL Bond Elut C18 cartridge (Agilent, P/N 12256130) was used for the solid phase extraction. A cartridge was conditioned with MeCN with 0.1% TFA (~10 mL), and then equilibrated with 1% MeCN in water with 0.1% TFA (~10 mL). Afterward, the library solution was loaded, and the cartridge was washed with 1% MeCN in water with 0.1% TFA (~10 mL). Sample elution was achieved by passing 70% MeCN in water with 0.1% TFA (~10 mL) through the cartridge. The final eluate was collected separately and lyophilized.

### HPSEC-based in-solution affinity selection

High-performance size exclusion chromatography (HPSEC) was carried out using an Agilent 1260 Infinity II LC System with the Agilent BIOSEC-3 HPLC column (7.8 × 150 mm, 3 μm particle size, 100 Å pore size). HPSEC samples, such as proteins, libraries, or protein-library mixtures (30 min incubation at 4 °C), were prepared in 100 μL buffer, and then eluted in buffered mobile phase at 1 mL/min flow rate for 15 min. While performing affinity selection experiments, the protein-binder complex fraction was detected by UV (214 and 280 nm) and collected. Prior to the LC-MS/MS-based *de novo* peptide sequencing, the protein-binder fraction was lyophilized. After the HPSEC affinity selection experiments, SEC column was cleaned with an IPA/water/MeCN/MeOH (1:1:1:1, *v/v*) mixture containing 0.1% formic acid (FA).

### Method of adjusting affinity selection conditions

Stringency of affinity selection is adjusted by the ratio of protein and peptide library. Using our HPSEC method, a less stringent selection condition is achieved with a molar ratio of peptide:protein = 1:10, e.g., protein concentration 10 pM, peptide library per member concentration 1 pM, volume 100 µL. For a stringent selection condition, molar ratio of peptide:protein = 1:1000, e.g., protein concentration 10 pM, peptide library per member concretion 10 fM, volume 100 µL.

### Method for de novo peptide sequencing

The lyophilized sample was dissolved in 50 μL water containing 0.2% FA and 10% trichloroacetic acid (TCA), and then subjected to LC-MS/MS using an Agilent 6550 quadrupole time-of-flight LC-MS with an Agilent Zorbax 300SB C3 column (2.1 × 150 mm, 5 μm particle size, 300 Å pore size). Mobile phases were water with 0.1% FA (solvent A) and MeCN with 0.1% FA (solvent B). A linear gradient of 1% B to 61% B (34 min, flow rate: 0.5 mL/min) was used to perform liquid chromatography. Absolute MS/MS threshold was typically set to 1500 counts and selected precursor ions had 2+ and 3+ charges. MS/MS spectra were imported and analyzed using PEAKS Studio software from Bioinformatics Solutions. Quality control of the peptide library was conducted prior to screening (Supplementary Fig. 7 and Supplementary Table 3).

### Method of expected value calculation

The expected pairwise alanine percentage (Ala-Ala%)_Additive_ is calculated as the product of single alanine frequencies (Ala-Ala%)_Additive_. For example, the (Thr1, Ser2) pair has an expected (Ala-Ala%)_Additive_ = 0.56 × 0.39 = 0.22 (22%). The calculation is repeated for the other possible pairs of residues in the peptide sequence.

### Method of calculating standard deviation

To compute the potential error distribution in the expected pairwise alanine frequencies, we generated large sets (1000 sets) of randomized sequences. Each set contains several random sequences. The total number of sequences in each set is equal to the total number of sequences enriched from the experiment. The sequences were generated by a random generator weighted by the positional alanine substitutional frequencies. The theoretical additive double-mutant frequencies were computed from the 1000 sets of randomly-generated and independent alanine-substituted sequences, in which the randomization of each position is weighted by its positional alanine substitution frequency^14^. From the 1000 output matrices, we calculated the standard deviation (σ) for alanine-substituted pairs and the mean value of the pairwise substitutional frequencies. These pairwise substitutional frequencies represent the expected values that are numerically equal to the product of positional alanine frequencies.

With the experimentally observed sequences, we computed the observed pairwise alanine frequencies, then divided the simultaneous alanine substitutional frequencies to the total number of sequences, (Ala-Ala%)_Observed_. To evaluate the difference between the expected pairwise alanine substitutional frequencies and the observed alanine substitutional frequencies, we divided the difference by the standard deviation values obtained in the randomization process. This difference, or (Ala-Ala%)_Observed_–(Ala-Ala%)_Additive_ was compared to the standard deviation (σ) of the theoretical additive values calculated from the random sets of sequences and assessed by the z-test.

### Method of statistical model

To provide quantitative data on residue-specific contributions to binding affinity, positional alanine frequencies from the combinatorial scanning were converted to changes in Gibbs free binding energy (∆∆*G*_scanning_). This calculation assumes that the ratio of WT to Ala for each position (*n*_WT_*/n*_Ala_) approximates the ratio of equilibrium association constants *K*_A,WT_ to *K*_A,Ala_, such that ∆∆*G*_scanning_ is given by: *∆∆G*_Ala-WT_ = *RT* ln(*K*_A,WT_*/K*_A,Ala_) *= RT* ln(*n*_WT_*/n*_Ala_)^19^. By comparing the *∆∆G*_Ala-WT_ values calculated from combinatorial scanning reported here (∆∆*G*_scanning_) to the ∆∆*G*_binding_ values previously measured by point alanine mutagenesis^26^, we found the two correlated linearly (R^2^ = 0.88, Supplementary Fig. 1).

### Theoretical *K*_D_ analysis

The theoretical *K*_D_’ was computed by linear addition of the *K*_D_ values of single alanine mutants. Assuming substitution follows additivity, we calculated the theoretical *K*_D_’ by adding up the binding free energy of single alanine substituted peptides. Therefore, theoretical *K*_D_’ is given by the equation: *RT* ln(*K*_D_’/*K*_D,PMI_) = ∑ (*∆∆G*_binding_ of single alanine mutations) = *RT* ln(Π *K*_D_ ratio). *K*_D,PMI_ = 3.2 ± 1.1 nM^3^, as reported (Supplementary Table 2). For example, peptide **17** (TSFAEYWAALSPK) has a theoretical *K*_D_’ given by the equation: *RT* ln(*K*_D_’/*K*_D,PMI_) = *RT* ln(0.2 $\times$ 0.8), such that the theoretical *K*_D_’ = 0.2 $\times$ 0.8 $\times$ 3.2 nM = 0.5 nM.

### In-solution competition assay

A competition binding assay was performed as described below using ForteBio Octet RED96 BLI system (Octet RED96) to estimate the binding affinity of peptides.

#### Calibration curve

Streptavidin sensors were soaked in blocking buffer (PBS supplemented with 0.05% Tween-20 and 1 mg/mL bovine serum albumin) for 5 min. After immobilizing the PEG4-biotinylated^15–29^ p53 peptide (100 nM of Biotin-PEG4-SQETFSDLWKLLPEN) onto streptavidin sensors, serial dilutions of SUMO-^25-109^MDM2 in blocking buffer were analyzed for binding. The response was recorded at equilibrium after 2 min. A curve of sensor response (nm) vs. MDM2 concentration (nM) was generated to calibrate the free MDM2 concentration in solution observed in the competition assay. The curve was generated using Prism 8.

#### ﻿Competition assay

Various concentrations of peptides were incubated in wells with a constant concentration of SUMO-^25-109^MDM2 protein (either ~50 nM or ~100 nM, as described for each peptide in the Supplementary Method 2) in the blocking buffer for 30 min. The PEG4-biotinylated p53^15–29^ peptide was immobilized onto streptavidin sensors and dipped into preincubated sample wells. The association events were measured at 30 °C, 1,000 rpm. Response at equilibrium after 2 min was recorded. Based on the binding response (nm) values, the concentration of ‘free’ SUMO-MDM2 (Y) was interpolated for each sample using the calibration curve (Supplementary Method 4). Nonlinear regression analysis was performed using the GraphPad Prism 8 software to estimate the *K*_D_ value based on the equation:

${K}_{D}=\frac{{Free}[{peptide}]\times {Free}[{MDM}2]}{[{peptide}-{protein\; complex}]}=\frac{[X\,-\,\left(b\,-\,Y\right)]\times Y}{(b\,-\,Y)}$, where Y is ‘free’ [SUMO-MDM2] in nM determined as described above, X is the total [peptide] in nM, *K*_D_ is the dissociation constant in nM, and b is the maximum [SUMO-MDM2] value fitted by the equation (Y_max_), equivalent to the total SUMO-MDM2 concentration provided in the assay. Reorganizing the quadratic equation for Y we obtain: Y^2^ + (*K*_D_ + X − b) × Y − b × *K*_D_ = 0. Solving the equation for the positive Y value, we arrived at the following equation used to generate the fitted curves and calculate *K*_D_: Y = 0.5 × [b − *K*_D_ − X + ((*K*_D_ + X − b)^2^ + 4b × *K*_D_)^0.5^]. In fitting this equation, the parameters *K*_D_ and b (Y_max_, the maximum possible protein concentration) were fitted by the software. The fitted Y_max_ value was compared in each case to the nominal total protein concentration used in the assay. After the regression analysis, we set the following criteria for the fit to be considered acceptable: (1) the fitted Y_max_ value should be within 10% of the nominal total SUMO-MDM2 concentration, (2) the fitted curve should reach a plateau at the lowest peptide concentration(s) used in the fit. The Y_max_ value was not fixed in any case. If either of the two criteria were not met for a given fit, the binding study was repeated with fresh solutions of the peptides and protein.

### Reporting summary

Further information on research design is available in the Nature Research Reporting Summary linked to this article.

## Supplementary information

Supplementary Information

Reporting Summary

## Data Availability

All data generated during this study are available either in the main text or supplementary materials.
