# Supplementary material for: Binary combinatorial scanning reveals potent poly-alanine-substituted inhibitors of protein-protein interactions
Source: Commun Chem. 2022 Oct 14;5:128. doi: 10.1038/s42004-022-00737-w (PMC9814900; doi:10.1038/s42004-022-00737-w)
Supplement: Supplementary file 1 — Supplementary Information [file 42004_2022_737_MOESM1_ESM.pdf]

Supplementary Information for

Binary combinatorial scanning reveals potent poly-alanine-substituted inhibitors of protein-protein interactions

Xiyun Ye,[1†] Yen-Chun Lee,[1†,a] Zachary P. Gates,[1,b] Yingjie Ling,[2] Jennifer C. Mortensen,[2] Fan-Shen Yang,[3] Yu-Shan Lin,[2] Bradley L. Pentelute[1,4,5,6*]

[1] Department of Chemistry, Massachusetts Institute of Technology, 77 Massachusetts Avenue, Cambridge, MA 02139, USA

[2] Department of Chemistry, Tufts University, 62 Talbot Avenue, Medford, MA 02155, USA

[3] Department of Chemistry and Frontier Research Center on Fundamental and Applied Sciences and Matters, National Tsing Hua University, 101, Sec. 2, Guang-Fu Road, Hsinchu 300, Taiwan

[4] The Koch Institute for Integrative Cancer Research, Massachusetts Institute of Technology, 500 Main Street, Cambridge, MA 02142, USA

[5] Center for Environmental Health Sciences, Massachusetts Institute of Technology, 77 Massachusetts Avenue, Cambridge, MA 02139, USA

[6] Broad Institute of MIT and Harvard, 415 Main Street, Cambridge, MA 02142, USA

* Correspondence to: blp@mit.edu

† These authors contributed equally to this work

[a] *Current address*: Department of Chemistry, National Cheng Kung University, No.1, University Road, Tainan City 701, Taiwan

[b] *Current address*: p53Lab, Agency for Science, Technology and Research (A*STAR), 8A Biomedical Grove, Singapore 138648, Singapore

Contents

1. Supplementary Note 1: Statistic analysis of alanine matrix 2
2. Supplementary Methods 1: Preparation of combinatorial alanine library 7
2.1 Preparation of PMI-based combinatorial alanine library 7
2.2 Preparation of HA tag-based combinatorial alanine library 10
2.3 Preparation of 14-3-3 binder-based combinatorial alanine library 11
3. Supplementary Methods 2: In-solution affinity selection of combinatorial alanine library 13
3.1 Method for HPSEC-based in-solution affinity selection 13
3.2 Method for *de novo* peptide sequencing 13
3.3 Affinity selection against MDM2 13
3.3.1 Selection conditions 13
3.3.2 Identified sequences from condition A 14
3.3.3 Identified sequences from condition B 15
3.4 Affinity selection with 12ca5 21
3.4.1 Selection conditions 21
3.4.2 Identified sequences from condition A 22
3.4.3 Identified sequences from condition B 22
3.4.4 Identified sequences from condition C 23
3.5 Affinity selection against 14-3-3 23
3.5.1 Selection condition 23
3.5.2 Identified sequences 23
4. Supplementary Methods 3: Method of molecular docking 24
4.1 Preparation of structures 24
4.2 Docking Experiment 25
4.3 Docking result 25
5. Supplementary Note 2: Peptide characterization and validation 26
5.1 (i, i+4) alanine-substituted peptides 26
5.2 (i, i+4) perfluoroaryl stapled peptides 30
5.3 Multi-alanine-substituted peptides 34
5.4 Multi-alanine-substituted stapled peptides 43
6. Supplementary Methods 4: Binding validation by bio-layer interferometry 46
6.1 Methods for BLI assay 46
6.2 Methods for BLI competition assay 47
6.3 Results of competition BLI assay 49
7. Supplementary References 62

1. Supplementary Note 1: Statistic analysis of alanine matrix

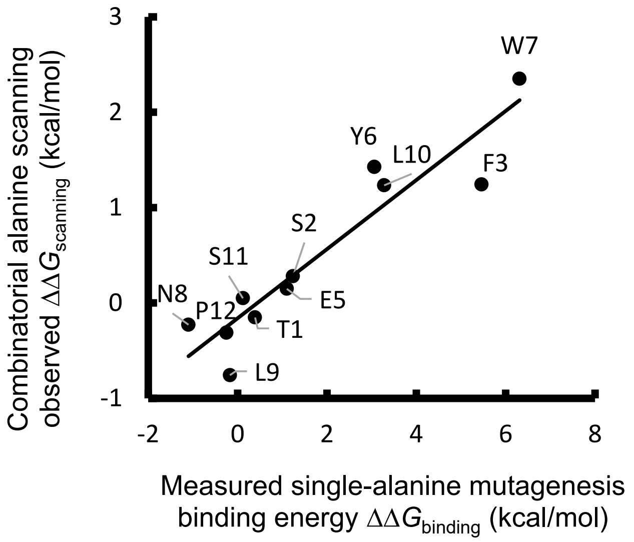

Supplementary Fig. 1. ΔΔG correlation between combinatorial alanine scanning and single alanine-mutagenesis. $\Delta\Delta G$ Correlation between the change of Gibbs free binding energy observed with combinatorial alanine scanning ($\Delta\Delta G_{scanning}$) and the reported change of Gibbs free binding energy ($\Delta\Delta G_{binding}$) measured by binding assay[3]. R^2 = 0.88. The linear correlation was fitted by the equation: $y = 0.36x - 0.16$.

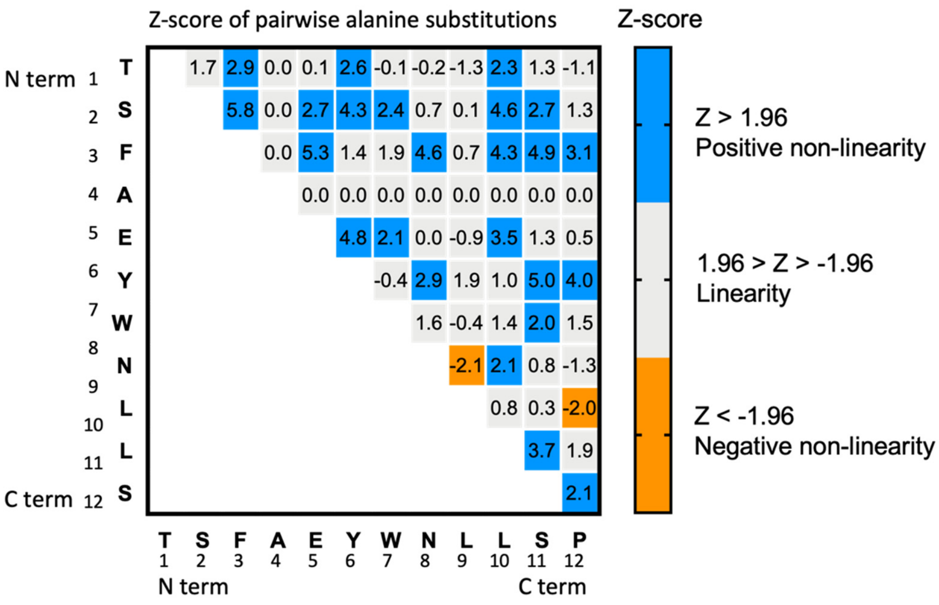

Supplementary Fig. 2. Statistic z-test analysis of pairwise alanine substitution frequency analysis. Z-score matrix of pairwise alanine substitutions. The number in each box of the matrix is the z-score calculated as $(\mathrm{Ala} - \mathrm{Ala\%})_{\mathrm{Observed}} - (\mathrm{Ala} - \mathrm{Ala\%})_{\mathrm{Additive}}$, (Additive = assuming the effects are additive). At 95% confidence level, doubly alanine-substituted pairs with a z-score within the range of -1.96 to 1.96 are a linear addition of single alanine substitution frequencies, marked in grey. The pairs with z-score larger than 1.96 are a positive non-linear addition of single alanine substitution frequency, marked in blue. The pairs with z-score smaller than -1.96 are negative non-linear addition of single alanine substitution frequency, marked in orange.

	T 1 (N term)	S 2	F 3	A 4	E 5	Y 6	W 7	N 8	L 9	L 10	S 11	P 12 (C term)
N term 1 T		11	0	0	15	0	0	26	46	0	26	26
2 S			0	0	7	0	0	15	23	0	11	19
3 F				0	0	0	0	0	0	0	0	0
4 A					0	0	0	0	0	0	0	0
5 E						0	0	23	30	0	11	26
6 Y							0	0	0	0	0	0
7 W								0	0	0	0	0
8 N									53	0	23	30
9 L										0	42	57
10 L											0	0
C term 11 S												26

Supplementary Fig. 3. **Pairwise alanine matrix of the enriched sequences from a stringent selection condition.** The pairwise alanine tolerance is indicated by a substitution frequency matrix. Each box represents the pairwise alanine substitution frequency (Ala-Ala%) of two residues, calculated as the ratio of number of observed simultaneous pairwise alanine substitutions to the total number of identified sequences, expressed as Ala-Ala% = $[(n_{\text{Ala,Ala}})/n_{\text{total}}] \times 100\%$. List of sequences is showed in supplementary information session 3.3.2.

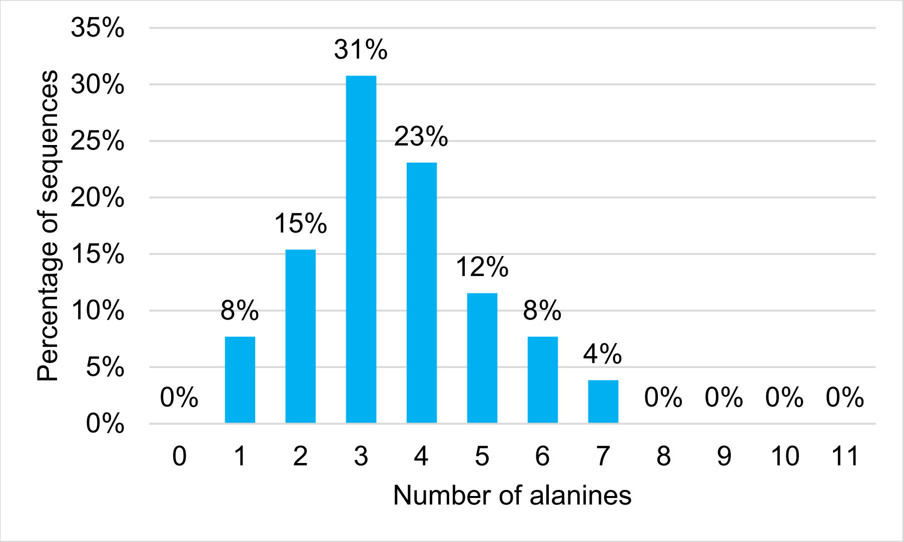

Supplementary Fig. 4. Distribution of number of alanine residues per sequence. The alanine distribution of the peptide sequences identified from the MDM2 affinity selection condition A (see section 4.3.2). The x-axis groups the sequences by their number of alanine residues and the y-axis shows the percentage of sequences that contain certain number of alanine residues. Alanine-substituted sequences with three and four substitutions were the most commonly identified sequences. Wild-type alanine is excluded from the analysis.

	T	S	F	A	E	Y	W	N	L	L	S	P
**T**		22	5	56	25	4	1	33	44	7	27	35
**S**			4	39	17	3	1	23	30	5	19	24
**F**				9	4	1	0	5	7	1	4	6
**A**					44	7	2	59	78	12	48	62
**E**						3	1	26	34	5	21	27
**Y**							0	4	5	1	3	4
**W**								1	2	0	1	1
**N**									46	7	28	37
**L**										9	37	48
**L**											6	7
**S**												30

Supplementary Fig. 5. Matrix of expected pairwise alanine percentage. Each box represents the pairwise alanine substitution frequency (Ala-Ala%)$_{exp}$ of two residues, calculated as the product of single positional alanine percentage.

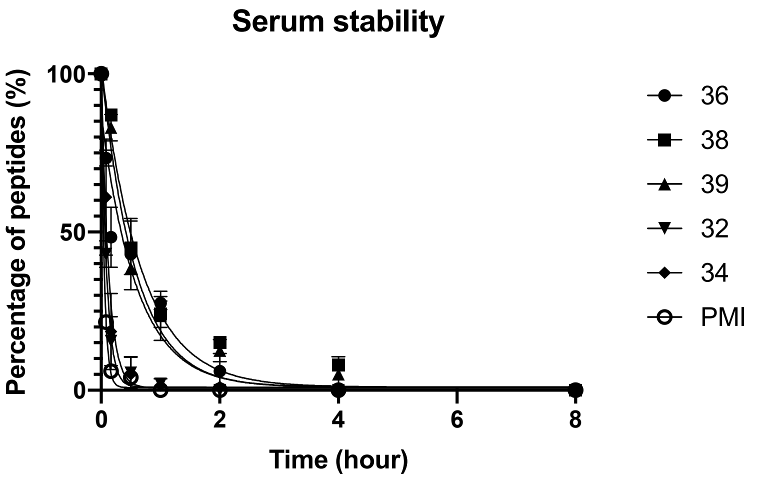

Compound name	PMI	32	34	36	38	39
Half-life (min)	2.2	3.9	5.1	26	27	25

Supplementary Fig. 6. Serum stability assay of peptide PMI, **32**, **34**, **36**, **38**, and **39**. 10 μM peptide were incubated with 25% human serum in buffer 1x PBS (*v/v*). At each time point (0 min, 5 min, 10 min, 30 min, 1 h, 2 h, 4 h, 8 h, 12h and 24 h), the mixture was sampled, treated with 10% trichloroacetic acid, and centrifuged at 12,000 *g* to remove serum proteins. The supernatant was acidified with 0.2% formic acid in water (*v/v*) and subjected to LC-MS analysis. The percentage of peptides (Y-axis) was calculated as the ratio of peak area in extracted ion chromatogram (EIC). Three experiments were carried out. The serum digestion curve was plotted using Prism8 software. The half-life of each peptide was calculated by fitted the digestion curve with the first order kinetic equation: $[X]_t = [X]_0 \times e^{-kt}$, $[X]$ is the remaining peptide percentage, t is the time in min. Half-life was calculated as $t_{1/2} = (\ln 2)/k$.

**Name**	**Peptide**	**Affinity of Best Pose (kcal/mol)**
PMI	TSFAEYWNLLSP-NH_2	–8.3
PMI-K	TSFAEYWNLLSPK-NH_2	–7.9
PMI-7Ala	AAFAAYWAALAA-NH_2	–11.7
PMI-K-7Ala (Peptide 34)	AAFAAYWAALAAK-NH_2	–10.4

Supplementary Table 1. Molecular docking result. Binding affinity of the best poses of the four peptide ligands to MDM2 inferred by molecular docking. Simultaneous Ala substitutions of all 7 non-hot spot residues (residues 1, 2, 5, 8, 9, 11, and 12) improves the calculated binding affinity with a $\Delta\Delta G$ of –3.4 kcal/mol for PMI and –2.5 kcal/mol for PMI-K. The addition of Lys impairs the binding affinity by 0.4 kcal/mol for PMI wild type and by 1.3 kcal/mol for PMI mutant.

Name	Sequence	K_D ratio[3]
PMI	TSFAEYWNLLSP	1.0
T1A	ASFAEYWNLLSP	1.9
S2A	TAFAEYWNLLSP	8.4
F3A	TSAAEYWNLLSP	11750
A4A	TSFAEYWNLLSP	1.0
E5A	TSFAAYWNLLSP	6.7
Y6A	TSFAEAWNLLSP	191
W7A	TSFAEYANLLSP	50720
N8A	TSFAEYWALLSP	0.2
L9A	TSFAEYWNALSP	0.8
L10A	TSFAEYWNLASP	227
S11A	TSFAEYWNLLAP	1.2
P12A	TSFAEYWNLLSA	0.7

Supplementary Table 2. Theoretical K_D' calculation. Theoretical K_D' is given by equation: $RT \ln(K_D'/K_{D,PMI}) = \sum (\Delta\Delta G_{binding}$ of single alanine mutations$) = RT \ln(\Pi\ K_D$ ratio$)$, using the reported $K_{D,PMI} = 3.2 \pm 1.1$ nM[3]. For example, peptide **17** (TSFAEYWAALSPK) has a theoretical K_D' given by the equation:

$RT \ln(K_D'/K_{D,PMI}) = RT \ln(0.2 \times 0.8)$, such that the theoretical $K_D' = 0.2 \times 0.8 \times 3.2$ nM $= 0.5$ nM.

2. Supplementary Methods 1: Preparation of combinatorial alanine library

2.1 Preparation of PMI-based combinatorial alanine library

Wide type sequence: TSFAEYWNLLSP
Library design: $(X)_{12}K\text{-}CONH_2$ (X = WT or Ala),
Library size: 2,048

Split-and-pool SPPS:

Coupling:

400 mg of 130 µm TentaGel resin (0.26 mmol/g, 0.10 mmol, 782770 beads/g, 3.13 x 10^5 beads) was placed in a 10 mL polypropylene syringe containing a porous polypropylene disc (Torviq). After swelling in DMF for 10 min, the resin was washed with DMF (3x). Fmoc-Rink amide linker (560 mg, 1.0 mmol, 10.0 equiv) was dissolved in 0.38 M HATU solution in DMF (2.4 mL, 0.49 mmol, 9.0 equiv), activated with DIEA (544 µL, 1.56 mmol, 30.0 equiv). The mixture was sonicated briefly, transferred to the fritted syringe containing the resin, and allowed to stir for 20 minutes. After the coupling solution was drained, and the resin was washed with DMF (3x). Fmoc protection group was achieved by soaking the resin with 20% piperidine in DMF (6 mL) for 5 min for twice, and then washed with DMF (3x). A sequential Fmoc-Lys(Boc)-OH coupling, Fmoc removal and DMF washes were performed in the same manner.

Portioning:

The peptidyl resin was suspended in DMF (6 mL), homogenized, then evenly divided among two 10 mL Torviq fritted syringes. After draining DMF from the syringes, the couplings were carried out as follows: Fmoc-protected WT amino acid and Fmoc-Ala-OH (0.26 mmol) were separately dissolved in 0.38 M HATU (0.66 mL, 0.26 mmol), activated with DIEA (136 µL, 0.36 mmol). Each of the activated amino acids was added to the individual resin-containing syringes (~200 mg, 0.052 mmol). After coupling for 20 min, the coupling solution was removed, and the resin was washed with DMF (3x). Then, all portions of peptidyl resin were combined and washed with DMF. Fmoc deprotection was achieved by treating the resin with 20% piperidine in DMF (6 mL, 5 min batch treatment), and this step was repeated twice. The resulting resin was then washed with DMF (3x). Twelve cycles of split-and-pool synthesis were performed using this procedure.

Cleavage from resin and global side chain deprotection:

The library was cleaved from resin and globally deprotected by treating the peptidyl resin with a cleavage cocktail containing 94% TFA, 2.5% EDT, 2.5% water, and 1.0% TIPS (v/v), for 1 h at ambient temperature. TFA was removed under a gentle stream of nitrogen gas, and the crude peptide was precipitated using cold Et_2O (-80

°C). After centrifugation at 3220 rcf for 3 min, the supernatant was removed, and the precipitated peptide was triturated three times with cold Et_2O. The resulting material was dissolved in 50% MeCN in water with 0.1% TFA, and lyophilized.

Solid phase extraction:

The library (60 mg) was dissolved in 6.0 mL of 5% MeCN in water with 0.1% TFA. 6 mL Bond Elut C18 cartridge (Agilent, P/N 12256130) was used for the solid phase extraction. A cartridge was conditioned with MeCN with 0.1% TFA (~10 mL), and then equilibrated with 1% MeCN in water with 0.1% TFA (~10 mL). Afterward, the library solution was loaded, and the cartridge was washed with 1% MeCN in water with 0.1% TFA (~10 mL). Sample elution was achieved by passing 70% MeCN in water with 0.1% TFA (~10 mL) through the cartridge. The final eluate was collected separately and lyophilized.

Quality control:

20 mg resin was weighed out, cleaved and analyzed by LC-MS/MS (20 mg loaded resin should theoretically contained 52 unique peptides; 52 = number of beads / copies of each peptide, number of beads = 7826, copies of peptide = number of total beads / library size = 3.1 x 10^5 beads / 2,048 = 151). 55 unique peptide sequences were identified. The library passes the quality control if the amino acid percentage at each position was equally distributed.

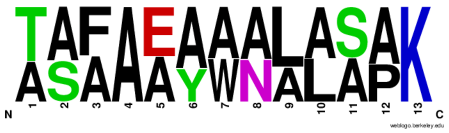

Supplementary Fig. 7. An example weblogo plot of a peptide library with equally distributed amino acid percentages.

Entry	Peptide	ALC (%)	m/z	z	Mass
1	TSFAEYWALLAPK	99	748.399	2	1494.7871
2	AAFAEAWALLAAK	99	666.3796	2	1330.7397
3	AAAAEAAAAASPK	98	549.8004	2	1097.5828
4	AAAAAAANAAAAK	98	521.2947	2	1040.5726
5	TAAAEAAAAASPK	98	564.806	2	1127.5935
6	AAAAAAAAAASPK	98	520.7975	2	1039.5774
7	AAAAAAAAAASAK	98	507.7916	2	1013.5618
8	TAAAEAANLASAK	98	594.3304	2	1186.6306
9	TAAAAAAAAASAK	98	522.7954	2	1043.5723
10	AAAAEAAALAAAK	98	549.814	2	1097.6191
11	TAFAEAAAALAAK	98	602.8406	2	1203.6611
12	AAFAAAAALAAAK	98	558.8314	2	1115.645
13	TSFAEYWALAAPK	98	727.3809	2	1452.74
14	TAFAAAWALAAPK	98	644.3667	2	1286.7134
15	AAFAEAWALAAPK	98	658.366	2	1314.7085
16	TAFAAAWAALAAK	98	631.3601	2	1260.6978
17	TAFAEAWALAAAK	98	660.3489	2	1318.7034
18	TAFAEAWAALAPK	98	673.3563	2	1344.719
19	AAFAEYWALAAPK	98	704.3668	2	1406.7346
20	TAFAEYWAALAPK	98	719.3831	2	1436.7451
21	AAFAAYWALAAAK	98	662.3675	2	1322.7134
22	TAFAEYWAALAAK	98	706.3785	2	1410.7295
23	TSFAAAWALLAPK	98	673.3871	2	1344.7554
24	TSFAAYWALLAAK	98	706.3895	2	1410.7659
25	TAFAEAWNLLAAK	98	702.8884	2	1403.7561
26	AAFAEAWALLAPK	98	679.3875	2	1356.7554
27	TAFAEAWALLSPK	98	702.3924	2	1402.7607
28	TAFAAAWALLSAK	98	660.3813	2	1318.7397
29	TAFAEYWALLAAK	98	727.3877	2	1452.7766
30	TAFAEAWALLAPK	98	694.3824	2	1386.7659

Supplementary Table 3. Table of identified sequences in the quality control. The first 30 sequences were shown.

2.2 Preparation of HA tag-based combinatorial alanine library

Wide type sequence: YPYDVPDYA

Library design: $(X)_9$(β-Ala)K-$CONH_2$ (X = WT or Ala)

Library size: 256

Split-and-pool synthesis:

Coupling

200 mg of 130 µm TentaGel resin (0.26 mmol/g, 0.052 mmol, 782770 beads/g, 1.57 x 10^8 beads) was placed in a 5 mL Torviq fritted syringe. After swelling in DMF for 10 min, the resin was then washed with DMF (3x). Fmoc-Rink amide linker (140 mg, 0.26 mmol, 5.0 equiv) was dissolved in 0.38 M HATU solution in DMF (0.65 mL, 0.25 mmol), activated with DIEA (63 µL, 0.36 mmol, 6.9 equiv). The mixture was sonicated briefly, transferred to the fritted syringe containing the resin, and allowed to stir for 20 minutes. After the coupling solution was drained, the resin was washed with DMF (3x). Fmoc deprotection was achieved by soaking the resin with 20% piperidine in DMF (v/v) (3 mL) for 5 min and this step was repeated twice. The resulting resin was then washed with DMF (3x). A sequential Fmoc-Lys(Boc)-OH coupling and Fmoc-β-Ala-OH were performed in the same manner.

Portioning:

The peptidyl resin was suspended in DMF (6 mL), homogenized, then evenly divided among two 10 mL Torviq fritted syringes. After draining DMF from the syringes, the couplings were carried out as following: Fmoc-protected WT amino acid and Fmoc-Ala-OH (0.13 mmol) were separately dissolved in 0.38 M HATU (0.33 mL, 0.13 mmol), activated with DIEA (32 µL, 0.18 mmol). Each of the activated amino acids was added to the individual resin-containing syringes (~100 mg, 0.026 mmol). After coupling for 20 min, the coupling solutions were drained, and the resin was washed with DMF (3x). Then, all portions of peptidyl resin were combined and washed with DMF. Fmoc deprotection was achieved by treating the resin with 20% piperidine in DMF (3 mL, 5 min batch treatment) and this step was repeated twice. The resulting resin was then washed with DMF (3x). Nine cycles of split-and-pool synthesis were performed using this procedure.

Cleavage from resin and global side chain deprotection:

The library was cleaved from resin and globally deprotected by treating the peptidyl resin with a cleavage cocktail containing 94% TFA, 2.5% EDT, 2.5% water, and 1.0% TIPS (*v/v*), for 1 h at ambient temperature. TFA was removed under a gentle stream of nitrogen gas, and the crude peptide was precipitated by the addition of cold

Et_2O (-80 °C). After centrifugation at 3220 rcf for 3 min, the supernatant was removed, and the precipitated peptide was triturated three times with cold Et_2O. The resulting material was dissolved in 50% MeCN in water with 0.1% TFA, and lyophilized.

Solid phase extraction:

The library (30 mg) was dissolved in 3.0 mL of 5% MeCN in water with 0.1% TFA. 6 mL Bond Elut C18 cartridge (Agilent, P/N 12256130) was used for the solid phase extraction. A cartridge was conditioned with MeCN with 0.1% TFA (~10 mL), and then equilibrated with 1% MeCN in water with 0.1% TFA (~10 mL). Afterward the library solution was loaded, and the cartridge was washed with 1% MeCN in water with 0.1% TFA (~10 mL). Sample elution was achieved by passing 70% MeCN in water with 0.1% TFA (~10 mL) through the cartridge. The final eluate was collected and lyophilized.

Quality control:

20 mg resin was weighed out, cleaved and analyzed by LC-MS/MS (20 mg loaded resin should theoretically contain 14 unique peptides; 14 = number of beads / copies of each peptide, number of beads = 7826, copies of peptide = number of total beads / library size = 1.5 x 10^5 beads / 256 = 585). 25 unique peptide sequences were identified. The library passes the quality control if the amino acid percentage at each position was equally distributed.

2.3 Preparation of 14-3-3 binder-based combinatorial alanine library

Wide type sequence: Cha Cha β-Ser Orn pSer Nph β-Ser β-Ser Nph
Library design: $(X)_9$K-$CONH_2$ (X = WT or Ala)

Abbreviations: Cha, cyclohexyl alanine; β-Ser, beta-homoserine; Orn, ornithine; Nph, 4-nitro-phenylalanine: pSer, phosphoserine. (b = Cha, T = β-Ser, c = Orn, d = pSer, e = Nph).

Split-and-pool synthesis:

Coupling:

200 mg of 130 µm TentaGel resin (0.26 mmol/g, 0.05 mmol, 782770 beads/g, 3.91 x 10^4 beads) was placed in a 5 mL Torviq fritted syringe. After swelling in DMF for 10 min, the resin was then washed with DMF (3x). Fmoc-Rink amide linker (268 mg, 0.50 mmol, 10.0 equiv) was dissolved in 0.38 M HATU solution in DMF (1.19 mL, 0.45 mmol, 9.0 equiv), activated with DIEA (260 µL, 1.5 mmol, 30.0 equiv). The mixture was sonicated briefly, transferred to the fritted syringe containing the resin, and allowed to stir for 20 minutes. After the coupling solution was drained, the resin was washed with

DMF (3x). Fmoc deprotection was achieved by soaking the resin with 20% piperidine in DMF (4 mL) for 5 min and this step was repeated twice. The resulting resin was then washed with DMF (3x). A sequential Fmoc-Lys(Boc)-OH coupling, Fmoc removal and DMF washes were performed in the same manner.

Portioning:

The peptidyl resin was suspended in DMF (6 mL), homogenized, then evenly divided among two 10 mL Torviq fritted syringes. After draining DMF from the syringes, the couplings were carried out as follows: Fmoc-protected WT amino acid and Fmoc-Ala-OH (0.5 mmol, 10.0 equiv.) were separately dissolved in 0.38 M HATU (0.59 mL, 0.22 mmol), activated with DIEA (130 μL, 0.75 mmol). Each of the activated amino acids was added to the individual resin-containing syringes (~100 mg, 0.025 mmol). After coupling for 20 min, the coupling solutions were drained, and the resin was washed with DMF (3x). Then, all portions of peptidyl resin were combined and washed with DMF. Fmoc deprotection was achieved by treating the resin with 20% piperidine in DMF (3 mL, 5 min batch treatment) and this step was repeated twice. The resulting resin was then washed with DMF (3x). Twelve cycles of split-and-pool synthesis were performed using this procedure.

Cleavage from resin and global side chain deprotection:

The library was cleaved from resin and globally deprotected by treating the peptidyl resin with a cleavage cocktail containing 94% TFA, 2.5% EDT, 2.5% water, and 1.0% TIPS (*v/v*), for 1 h at ambient temperature. TFA was removed under a gentle stream of nitrogen gas, and the crude peptide was precipitated by the addition of cold Et_2O (-80 °C). After centrifugation at 3220 rcf for 3 min, the supernatant was removed, and the precipitated peptide was triturated three times with cold Et_2O. The resulting material was dissolved in 50% MeCN in water with 0.1% TFA, and lyophilized.

Solid phase extraction:

The library (~20 mg) was dissolved in 5 mL of 5% MeCN in water with 0.1% TFA. 6 mL Bond Elut C18 cartridge (Agilent, P/N 12256130) was used for the solid phase extraction. A cartridge was conditioned with MeCN with 0.1% TFA (5 mL), and then equilibrated with 1% MeCN in water with 0.1% TFA (5 mL). Next, the library solution was loaded, and the cartridge was washed with 1% MeCN in water with 0.1% TFA (5 mL). Sample elution was done by 70% MeCN in water with 0.1% TFA (5 mL). The eluted fraction was collected and lyophilized.

Quality control:

20 mg resin was weighed out, cleaved and analyzed by LC-MS/MS (20 mg loaded resin should theoretically contained 27 unique peptides; 27 = number of beads / copies

of each peptide, number of beads = 7826, copies of peptide = number of total beads / library size = 1.5 x 10^5 beads / 512 = 293). 39 unique peptide sequences were identified. The library passes the quality control if the amino acid percentage at each position was equally distributed.

3. Supplementary Methods 2: In-solution affinity selection of combinatorial alanine library

3.1 Method for HPSEC-based in-solution affinity selection

High-performance size exclusion chromatography (HPSEC) was carried out using an Agilent 1260 Infinity II LC System with the Agilent BIOSEC-3 HPLC column (7.8 x 150 mm, 3 µm particle size, 100 Å pore size). HPSEC samples, such as proteins, libraries, or protein-library mixtures (30 min incubation at 4 °C), were prepared in 100 µL buffer, and then eluted in buffered mobile phase at 1 mL/min flow rate for 15 min. While performing affinity selection experiments, the protein-binder complex fraction was detected by UV (214 and 280 nm) and collected. Prior to the LC-MS/MS-based *de novo* peptide sequencing, the protein-binder fraction was lyophilized. After the HPSEC affinity selection experiments, SEC column was cleaned with an IPA/water/MeCN/MeOH (1:1:1:1, *v/v*) mixture containing 0.1% formic acid (FA).

3.2 Method for *de novo* peptide sequencing

The lyophilized sample was dissolved in 50 µL water containing 0.2% FA and 10% trichloroacetic acid (TCA), and then subjected to LC-MS/MS using an Agilent 6550 quadrupole time-of-flight LC-MS with an Agilent Zorbax 300SB C3 column (2.1 x 150 mm, 5 µm particle size, 300 Å pore size). Mobile phases were water with 0.1% FA (solvent A) and MeCN with 0.1% FA (solvent B). A linear gradient of 1% B to 61% B (34 min, flow rate: 0.5 mL/min) was used to perform liquid chromatography. Absolute MS/MS threshold was typically set to 1500 counts and selected precursor ions had 2+ and 3+ charges. MS/MS spectra were imported and analyzed using PEAKS Studio software from Bioinformatics Solutions.

3.3 Affinity selection against MDM2

3.3.1 Selection conditions

Condition[a]	MDM2	Library	per member[b]
**A**	1 nmol, 23 µg	4 µg	1 ng
**B1, B2, B3**	1 nmol, 23 µg	500 µg	122 ng

MDM2 affinity selection condition. [a]Library was incubated with MDM2 in 1x PBS (total volume: 100 µL) for 30 min in 4 °C before the HPSEC-based affinity selection. [b]Amount of each library member: amount of library divided by library diversity.

3.3.2 Identified sequences from condition A

The selection was replicated five times. The following list combined results from five repetitions.

Entry	Compound name	Sequence	ALC (%)	*m/z*	*z*	Mass	K_D (nM)	Mass error (ppm)	RT (min)
1	17	TSFAEYWAALSPK	95	735.3814	2	1468.7351	31.5 ± 4.2	1.3	15.51
2	18	TSFAEYWNALSAK	98	743.8728	2	1485.7251	2.9 ± 1.6	0.6	15.81
3	19	TSFAEYWNALAPK	99	748.8862	2	1495.7458	8.8 ± 1.9	0.4	15.93
4	20	TSFAEYWNALAAK	99	735.8782	2	1469.7302	6.2 ± 1.8	1.1	15.07
5	21	ASFAEYWAALSPK	99	720.3765	2	1438.7244	2.0 ± 1.8	-0.6	15.65
6	22	TSFAEYWAALSAK	99	722.3726	2	1442.7195	4.9 ± 2.7	-2.1	15.78
7	23	TAFAEYWNALSAK	99	735.8763	2	1469.7302	3.7 ± 1.5	0.1	16.13
8	24	ASFAEYWAALAPK	99	712.3774	2	1422.7295	59.3 ± 11.6	4.1	15.2
9	25	TSFAAYWAALSAK	85	693.3683	2	1384.7139	2.2 ± 0.8	3.9	15.24
10	26	TAFAEYWAALSAK	94	714.3749	2	1426.7244	1.0 ± 1.0	-1	16.26
11	27	AAFAEYWAALSPK	96	712.3769	2	1422.7295	9.9 ± 3.3	2.9	16.52
12	28	ASFAEYWNALAAK	92	720.8735	2	1439.7197	4.7 ± 1.5	-0.5	15.9
13	29	ASFAEYWAALAAK	93	699.3627	2	1396.703	2.3 ± 1.2	-1.2	16.6
14	30	ASFAAYWAALSAK	98	678.3644	2	1354.7034	1.9 ±1.9	-0.8	15.94
15	31	AAFAEYWNALAAK	95	712.8754	2	1423.7249	3.2 ± 2.6	2.4	15.53
16	32	TAFAAYWAALAAK	99	677.3744	2	1352.7241	3.4 ± 2.1	1.4	16.68
17	33	ASFAAYWAALAAK	95	670.3667	2	1338.7085	67.8 ± 14.4	-0.7	16.78
18	34	AAFAAYWAALAAK	97	662.3692	2	1322.7134	4.7 ± 2.4	0	16.50
19		TSFAEYWAALAPK	99	727.3827	2	1452.74		0.2	15.11
20		TSFAEYWNALSPK	98	756.883	2	1511.741		-2.7	16.43
21		TSFAAYWAALSPK	96	706.3765	2	1410.7295		-0.5	15.78
22		TSFAEYWNLLSAK	95	764.9009	2	1527.7722		2.2	16.84
23		ASFAEYWNALSPK	94	741.8788	2	1481.7302		0.7	15.3
24		ASFAEYWNALAPK	94	733.8811	2	1465.7354		2.2	16.84
25		TSFAAYWNALSAK	93	714.8728	2	1427.7197		-1.9	15.82
26		ASFAAYWNALSAK	81	699.8665	2	1397.7092		0.4	15.93

Identified sequences from MDM2 affinity selection condition A. Sequences were enriched from MDM2 affinity selection condition A with high average local confidence (ALC > 80). Peptides selected for resynthesizing and validation were given a compound number. Binding dissociation constant K_D was determined by BLI competition assay. RT = retention time.

3.3.3 Identified sequences from condition B

The selection was replicated for three times. On average, 79 unique sequences were identified. Selections with 73, 73, 90 unique identified sequences are listed.

Entry	Peptide	ALC (%)	*m/z*	*z*	Mass	RT (min)	Mass error (ppm)
1	TSFAEYWAALSPK	99	735.3752	2	1468.7351	13.91	-1
2	ASFAEYWAALAPK	99	712.3723	2	1422.7295	15.68	0.6
3	TSFAEYWAALAPK	99	727.3778	2	1452.74	16.01	0.3
4	TSFAEYWALLSAK	99	736.3823	2	1470.7507	16.04	0.7
5	TSFAEYWNALSAK	99	743.8708	2	1485.7251	16.21	-0.4
6	TAAAAAWALLAAK	99	614.3632	2	1226.7136	15.15	1.4
7	TSFAEYWNALAAK	99	735.8717	2	1469.7302	15.19	-1.5
8	AAFAEYWAALSAK	99	699.3636	2	1396.7139	15.62	-0.9
9	AAFAAYWNAAAAK	98	662.8452	2	1323.6724	15.72	-0.9
10	AAFAAYWAAASPK	98	662.3447	2	1322.677	12.51	2.7
11	ASFAEYWAAAAPK	98	691.3486	2	1380.6826	12.9	-1.6
12	TSFAEYWNALSPK	98	749.8688	2	1497.7251	13.52	0.1
13	ASFAEYWNALAAK	98	713.8593	2	1425.7041	14.05	-1.3
14	ASFAEYWAALSPK	98	713.3624	2	1424.7087	14.34	-0.1
15	TSFAEYWAALSAK	98	715.3613	2	1428.7036	14.46	-1.6
16	ASFAEYWNALSPK	98	741.8723	2	1481.7302	14.68	1.1
17	ASFAEYWNALAPK	98	733.8743	2	1465.7354	14.97	3.5
18	AAFAEYWAALSPK	98	712.3712	2	1422.7295	14.93	3.1
19	TSFAEYWNALAPK	98	748.8795	2	1495.7458	14.92	-0.1
20	ASFAEYWAALAAK	98	692.3597	2	1382.6982	14.99	-1.5
21	TSFAEYWNLLSAK	98	764.8924	2	1527.7722	15.23	-0.9
22	AAFAEYWNALSAK	98	720.8658	2	1439.7197	15.32	-1.1
23	TAFAEYWAALSAK	98	714.3677	2	1426.7244	15.32	-0.9
24	AAFAAAWNLLAAK	98	658.8769	2	1315.74	15.62	4.7
25	TSFAEYWNLLSPK	98	777.9005	2	1553.7878	15.68	1.6
26	ASFAEYWNALSAK	98	721.8557	2	1441.699	16.31	-1.3
27	AAFAAAWAALAAK	98	609.342	2	1216.6716	14.38	-1.8
28	TSFAEYWAALAAK	98	707.3628	2	1412.7087	15.51	-2.5
29	ASFAEYWALLSAK	98	721.377	2	1440.74	15.74	-0.6
30	TSFAEAWAALAPK	97	681.3595	2	1360.7139	15.75	1.7
31	ASFAAYWAALSAK	97	678.3594	2	1354.7034	16.57	-1
32	ASFAEYWAALSAK	97	707.3636	2	1412.7087	14.13	-1.5

33	AAAAEYWAALAAK	97	653.3528	2	1304.6877	14.97	-1.7
34	TAFAEYWNALSAK	97	735.8715	2	1469.7302	16.04	1.6
35	TSFAAYWAALSAK	97	693.3629	2	1384.7139	16.62	-0.4
36	ASFAEYWAAASPK	97	699.3471	2	1396.6775	14.34	-1.4
37	AAAAAYWALAAAK	97	624.3459	2	1246.6821	15.38	-6.9
38	ASAAAYWAALAPK	97	645.3525	2	1288.6929	15.65	0.7
39	AAFAAAWALAAAK	97	616.3502	2	1230.6873	15.6	2.7
40	TSFAEYWNLLAAK	97	749.8883	2	1497.7615	13.74	2.6
41	ASFAEYWNLLSAK	97	749.8866	2	1497.7615	14.69	-1.2
42	AAAAAYWAALAPK	96	637.3564	2	1272.6978	15.53	-1.9
43	AAFAEYWNALAAK	96	705.8611	2	1409.7092	13.39	1.6
44	AAFAEYWAALAPK	96	697.3643	2	1392.719	13.76	-3.9
45	TAFAEYWAALSPK	96	727.3778	2	1452.74	13.97	-1.9
46	AAFAAYAALLAAK	96	625.8669	2	1249.7183	15.28	-1.2
47	AAFAAYWAAAAPK	96	647.3444	2	1292.6665	16.33	0.4
48	AAAAAAWALLAAK	96	599.3575	2	1196.7029	16.65	-1.9
49	AAFAEYWALAAPK	96	704.3736	2	1406.7346	13.29	0.5
50	ASFAAAWAALAAK	95	617.3406	2	1232.6665	14.19	-1.1
51	AAAAAYWALAAPK	95	637.3544	2	1272.6978	15.1	-3.5
52	ASFAEYWNLASAK	95	721.8559	2	1441.699	15.47	-0.5
53	ASFAAYWNALSAK	95	699.8611	2	1397.7092	15.5	0.7
54	TSFAAYWNALSAK	95	714.8654	2	1427.7197	15.15	0.8
55	TSFAAYWAALSPK	95	706.3705	2	1410.7295	12.98	6
56	TSFAEYWALLSPK	95	749.3895	2	1496.7664	14.88	-2
57	AAAAAYWALASAK	95	632.3438	2	1262.677	15.45	-1.4
58	ASFAAYWAALSPK	95	691.3661	2	1380.719	14.52	0.2
59	AAFAEYWNALAPK	94	725.8757	2	1449.7405	13.26	-2.7
60	TSAAAAWALLAAK	94	622.3607	2	1242.7085	13.8	-1.2
61	TAFAAAWAALAPK	94	644.3613	2	1286.7134	14.78	-1.2
62	AAFAEYWAALAAK	93	691.3661	2	1380.719	14.73	-2.5
63	AAAAEYWALLAAK	93	667.3655	2	1332.719	14.97	-2.3
64	AAFAEYWNLASPK	92	733.8737	2	1465.7354	15.61	-2.2
65	ASFAEAWALLAAK	92	667.3688	2	1332.719	16.4	-1.3
66	ASFAAYAALLAAK	91	633.8636	2	1265.7131	12.94	-3.2
67	TSFAAYWAALAAK	91	685.3677	2	1368.719	15.02	-1.3
68	AAFAAYWAALSAK	91	670.3555	2	1338.7085	15.98	-0.9
69	AAFAEYWAAAAAK	91	670.3434	2	1338.6721	14.92	0
70	TAFAEYWAALAAK	91	706.371	2	1410.7295	14.91	-2.5

71	AAFAEYWNALSPK	89	733.874	2	1465.7354	16.5	0.6
72	AAFAAYWAAASAK	89	642.3295	2	1282.6458	14.83	-1.3
73	ASFAAAWAALSAK	88	632.3445	2	1262.677	15.63	-4.2
74	ASFAEYWNAAAAK	87	699.844	2	1397.6729	16.08	-1
75	AAAAAYWNLLSAK	87	667.8634	2	1333.7144	15.13	1.3
76	TAFAEYWAALAPK	87	719.3804	2	1436.7451	14.86	-1.9
77	TAAAAAWAALAAK	86	593.34	2	1184.6665	14.5	-1.8
78	TSAAEYWNLLAPK	86	731.8821	2	1461.7615	15.14	-1.7
79	TAFAEYWNALSPK	84	748.8786	2	1495.7458	16.32	3
80	ASAAAYWALAAAK	82	632.343	2	1262.677	15.19	-0.3
81	TAFAAAWNALAAK	81	652.8569	2	1303.7036	16.37	1.4
82	AAAAAYWAALSAK	81	625.3373	2	1248.6614	16.53	-0.2
83	AAAAAYWAALSPK	79	645.347	2	1288.6929	13.8	1.5
84	AAFAEYWNLASAK	78	720.8655	2	1439.7197	15.33	-9
85	ASFAAAWALLAPK	78	651.3713	2	1300.7292	14.19	0.1
86	ASFAAAWAALAPK	76	637.3546	2	1272.6978	16.68	-1.5
87	TAFAEYWNALAAK	75	727.8723	2	1453.7354	14.39	-4.4
88	ASFAEAWAALSAK	75	661.3501	2	1320.6826	14.53	-1.3
89	AAAAAYWNALAAK	74	645.8525	2	1289.688	12.67	-1.1
90	ASAAAYWNLLAPK	72	680.8703	2	1359.73	14.34	-0.3

Identified sequences from MDM2 affinity selection condition B1. (ALC >70)
RT = retention time.

Entry	Peptide	ALC (%)	*m/z*	*z*	Mass	RT (min)	Mass error (ppm)
1	AAAAAYWAALAAK	99	617.3381	2	1232.6665	13.36	-4
2	AAAAAYWALAAAK	99	624.343	2	1246.6821	13.68	-8.6
3	TSFAEYWAALAPK	99	727.3776	2	1452.74	16.53	0.4
4	AAAAAYWAALSAK	98	632.3411	2	1262.677	13.11	-7.5
5	AAFAAYWAAAAPK	98	654.3472	2	1306.6821	13.61	-1.8
6	ASFAAYWAAAAPK	98	662.3434	2	1322.677	13.89	-3.6
7	TSFAEYWAAAAPK	98	706.3522	2	1410.6931	13.93	-2.3
8	TSFAEYWNALAPK	98	748.8783	2	1495.7458	15.56	-2.5
9	AAFAAYWAALSPK	98	683.3693	2	1364.7241	15.64	0
10	TSFAEYWNALAAK	98	735.8724	2	1469.7302	15.65	0
11	AAFAAYWAALSAK	98	670.3605	2	1338.7085	15.74	-1.6
12	ASAAAYWAALAAK	97	625.3362	2	1248.6614	13.38	-2.9

13	TSFAEYWNALSAK	97	743.867	2	1485.7251	15.12	-3.8
14	TSFAEYWNALSPK	97	756.8762	2	1511.741	15.19	-2.1
15	TSFAAYWNALAAK	97	706.8688	2	1411.7249	15.56	-1.2
16	AAFAEYWAALSAK	97	699.3644	2	1396.7139	15.7	0.3
17	AAAAAYWNLLAAK	96	659.8677	2	1317.7192	14.09	1.3
18	AAFAAYWNALSAK	96	684.8502	2	1367.6985	14.25	-9.2
19	AAFAAYWNALSPK	96	704.8688	2	1407.73	14.95	-5
20	ASFAEYWAALSPK	96	713.36	2	1424.7087	15	-2.3
21	AAFAEYWAALAPK	96	697.3643	2	1392.719	15.41	-3.5
22	ASFAEYWNALAPK	96	733.8726	2	1465.7354	15.58	-3.2
23	AAFAEYWAALSPK	96	712.3723	2	1422.7295	15.59	0.3
24	ASFAAYWAALAPK	96	683.3708	2	1364.7241	15.94	0.9
25	AAAAAYWNLLAPK	95	679.8798	2	1357.7507	16.44	2.1
26	TAFAEYWNALAAK	95	720.8647	2	1439.7197	14.77	-4.2
27	ASFAEYWNALSPK	95	741.8707	2	1481.7302	14.89	-3.4
28	ASFAEYWAALAAK	95	692.3508	2	1382.6982	15.21	-2.2
29	ASFAEYWNLLSPK	95	755.8871	2	1509.7615	15.84	-8.1
30	TAFAEYWAALAPK	95	719.378	2	1436.7451	16.27	-1.1
31	AAFAAYWAAAAAK	94	634.3331	2	1266.6509	16.29	-2.6
32	ASAAAYWNLLAAK	94	667.86	2	1333.7144	13.57	0.6
33	ASFAAYWNALSAK	94	692.8503	2	1383.6936	14.02	-6.7
34	ASFAEYWNALAAK	94	713.8553	2	1425.7041	14.16	-5.4
35	AAFAAYWNLASPK	94	704.8688	2	1407.73	14.61	-5.7
36	TSFAEYWAALSPK	94	728.3641	2	1454.7195	14.95	-5
37	TAAAAAWALLAAK	94	614.3604	2	1226.7136	15.07	-4
38	ASFAAYWAALSAK	94	678.3605	2	1354.7034	15.13	-6
39	TSFAAYWAALSAK	94	693.3624	2	1384.7139	15.88	2.2
40	ASFAEYWAALAPK	94	712.3726	2	1422.7295	15.92	-2.6
41	AAAAAYWALLSPK	93	659.3653	2	1316.7241	16.42	0.8
42	TAFAEYWNALSAK	93	735.8701	2	1469.7302	14.28	-7.3
43	TSFAEYWAALSAK	93	715.3601	2	1428.7036	14.71	-6.1
44	TAFAEYWNALAPK	93	740.883	2	1479.751	14.96	-3.2
45	ASFAAYWNALAPK	93	704.8707	2	1407.73	15.23	1.4
46	ASFAAAWNLLAAK	93	666.8737	2	1331.7349	15.38	0.3
47	TSFAEYWNLLAAK	93	749.8869	2	1497.7615	15.44	-2.3
48	ASFAAYWAAASPK	92	670.3438	2	1338.6721	15.48	-7.9
49	AAFAAAWALLSAK	92	638.3632	2	1274.7134	16.13	-1.5
50	TAFAAAWNLLSAK	92	681.8802	2	1361.7456	16.31	-1.5

51	TSFAAYWAALAAK	92	685.3661	2	1368.719	13.49	0.6
52	AAFAAAWNLLAAK	91	658.8754	2	1315.74	16.11	-1.3
53	TSFAEYWALLSAK	91	736.3832	2	1470.7507	16.19	0.3
54	TSFAAYWNALSAK	90	714.8643	2	1427.7197	16.76	-1
55	ASFAAYWALASPK	90	691.3682	2	1380.719	16.17	-2.9
56	ASFAAYWAALAAK	90	670.3616	2	1338.7085	16.62	0.8
57	ASFAEYWNLASPK	89	741.8715	2	1481.7302	15.15	-4
58	AAFAAYWAAASPK	88	662.3378	2	1322.677	15.96	2
59	AAAAAYWAALAPK	88	637.3521	2	1272.6978	16.74	0.1
60	TAAAAYWAALAPK	88	652.3594	2	1302.7085	14.67	-1.2
61	ASFAAYWNLAAPK	88	697.8576	2	1393.7141	16.03	2.1
62	TSFAAYWNALSPK	88	720.8551	2	1439.7197	13.22	-12.1
63	ASAAAYWNLLAPK	88	687.8765	2	1373.7456	13.73	-6.4
64	ASFAAYWAALSPK	88	691.3682	2	1380.719	13.8	-3.3
65	ASFAEYWNLLAAK	88	734.8823	2	1467.751	14.24	-9.7
66	ASFAAAWAALSAK	87	632.343	2	1262.677	14.35	-9.8
67	AAFAAYAALLAAK	87	625.8641	2	1249.7183	14.39	-16.7
68	ASAAAYWALAAAK	86	632.3417	2	1262.677	14.81	-5.3
69	ASFAAYWNALAAK	86	691.8635	2	1381.7141	15.97	2
70	TSFAAYWAALAPK	85	698.3774	2	1394.7346	16.25	-0.6
71	AAFAEYWAALAAK	84	691.3651	2	1380.719	15.09	-4.4
72	TAFAEYWAALSAK	82	707.3613	2	1412.7087	15.62	-3.6

Identified sequences from MDM2 affinity selection condition B2 (ALC >70)
RT = retention time.

Entry	Peptide	ALC (%)	*m/z*	*z*	Mass	RT (min)	Mass error (ppm)
1	TSFAEYWAALAPK	99	727.3783	2	1452.74	16.46	1.4
2	ASAAAYWALAAAK	98	632.3451	2	1262.677	13.65	-1.1
3	ASFAEYWAAAAPK	98	691.3495	2	1380.6826	13.91	1.4
4	ASAAAYWAALAPK	98	645.3535	2	1288.6929	13.94	-0.3
5	TSFAEYWNALSAK	98	736.8605	2	1471.7095	14.34	-2
6	TSFAEYWNALAAK	98	728.8646	2	1455.7146	14.85	0
7	TSFAEYWAALSAK	98	715.3605	2	1428.7036	15.24	-1.2
8	TSFAEYWNALSPK	98	756.8768	2	1511.741	15.29	2
9	ASFAEYWAALSPK	98	720.3701	2	1438.7244	15.38	-1.3
10	TSFAEYWAALSPK	98	735.3755	2	1468.7351	15.73	-0.2

11	ASFAEYWAALAPK	98	712.3723	2	1422.7295	16	0.9
12	AAFAAYWAALAAK	98	662.3634	2	1322.7134	16.03	1
13	TSFAEYWALLSAK	98	736.3837	2	1470.7507	16.38	0.3
14	AAFAAYWAAASPK	97	662.3444	2	1322.677	16.63	-0.8
15	TAAAAAWALLAAK	97	614.3621	2	1226.7136	16.63	1.4
16	TSFAEYWNALAPK	97	748.8801	2	1495.7458	13.25	-2.1
17	AAFAAYAALLAAK	97	625.8654	2	1249.7183	15.24	-3.3
18	ASFAEYWAALSAK	97	707.3629	2	1412.7087	15.71	-0.2
19	TSFAEYWNLLSAK	97	764.8933	2	1527.7722	15.66	-1.7
20	AAFAAYWNAAAAK	96	662.8416	2	1323.6724	16.08	1.8
21	AAAAAYWALAAAK	96	624.3488	2	1246.6821	16.72	-0.1
22	AAFAEYWAAAAAK	96	670.3434	2	1338.6721	12.9	-2.9
23	ASFAEYWAALAAK	96	692.3596	2	1382.6982	13.79	0.8
24	TAFAAYWAALSAK	96	685.3663	2	1368.719	14.21	0.2
25	ASFAEYWNALSAK	95	721.8554	2	1441.699	16.02	4.7
26	AAFAAAWAALAAK	95	609.3421	2	1216.6716	16.02	-0.6
27	AAFAEYWAALSAK	95	699.3657	2	1396.7139	14.2	-1.9
28	TAFAEYWAALSAK	95	714.3693	2	1426.7244	15.03	-1.6
29	AAFAEYWAALAPK	95	704.3668	2	1406.7346	15.24	1.8
30	AAFAAAWNLLAAK	95	658.8762	2	1315.74	15.75	2.2
31	AAFAAYWAALAPK	95	675.3718	2	1348.7292	16.02	-0.2
32	AAAAAYWAALAPK	94	637.356	2	1272.6978	16.18	-0.9
33	TAFAEYWNALSAK	94	735.8719	2	1469.7302	16.15	-11.1
34	TSFAAYWNALAAK	94	706.8693	2	1411.7249	16.2	-1.7
35	AAFAAYWAALSAK	94	670.3616	2	1338.7085	16.22	-0.1
36	TSFAEYWNLLAAK	94	749.8884	2	1497.7615	13.74	-0.3
37	AAFAAYWNALSAK	93	691.8629	2	1381.7141	15.09	-0.7
38	AAFAEYWNALAAK	93	712.8685	2	1423.7249	15.45	0
39	ASFAEYWNALSPK	93	741.8715	2	1481.7302	15.67	-0.6
40	TAFAEYWNALAPK	93	740.8812	2	1479.751	15.89	0.1
41	ASFAEYWNALAAK	93	720.868	2	1439.7197	16.33	0.5
42	TAFAAAWNLLSAK	93	681.8788	2	1361.7456	14.95	-2.1
43	AAFAAYWAAASAK	92	642.3296	2	1282.6458	15.24	-1.7
44	TAAAAYWALASAK	92	647.3498	2	1292.6877	15.33	-1.3
45	AAFAEYWAALSPK	92	712.3741	2	1422.7295	15.43	-1.8
46	ASFAAYWAALSAK	92	678.3596	2	1354.7034	15.46	-2.1
47	TSFAAYWAALAAK	92	685.3661	2	1368.719	15.69	1.2
48	TSFAEYWAALAAK	92	714.3695	2	1426.7244	16.27	-1.8

49	TSFAAYWNALSAK	91	707.8601	2	1413.7041	12.76	-0.9
50	ASFAAYWNALSPK	91	705.8607	2	1409.7092	13.06	-2.1
51	AAAAAYWNLLSPK	91	687.8792	2	1373.7456	14.74	3.1
52	ASFAEYWNALAPK	91	733.8737	2	1465.7354	15.67	3
53	AAFAEYWNLAAAK	90	712.8698	2	1423.7249	15.97	1
54	TAFAAYWNALSAK	90	706.8688	2	1411.7249	16.85	-1
55	AAFAAYWALAAAK	90	655.3568	2	1308.6978	16.98	0.1
56	ASFAAYWNALSAK	89	692.8526	2	1383.6936	14.33	1.1
57	TAFAAAWNLLAAK	89	666.8742	2	1331.7349	14.44	-1.7
58	ASFAAYWAALSPK	88	691.3685	2	1380.719	14.75	-1.3
59	ASFAEYWNLLSAK	88	749.8881	2	1497.7615	15.57	-1.8
60	ASFAAAWAALAAK	87	617.3376	2	1232.6665	14.76	0.2
61	ASFAAAWAALSAK	87	632.3442	2	1262.677	15.19	-1.3
62	ASFAAAWALLSAK	86	646.3615	2	1290.7085	15.57	0.9
63	AAFAAYWAAAAPK	85	654.3486	2	1306.6821	14.27	-2.1
64	AAAAAYWNLLSAK	85	667.8629	2	1333.7144	15.07	1.9
65	ASFAAYWAALAPK	84	683.3719	2	1364.7241	15.16	-2.1
66	AAFAAAWALLAPK	83	643.3757	2	1284.7341	15.82	-0.8
67	TSFAAYWNALSPK	82	720.8656	2	1439.7197	15.97	2.5
68	AAFAEYWNALSAK	82	720.8672	2	1439.7197	16.7	0.1
69	AAFAAYWNALSPK	79	704.8701	2	1407.73	14.97	-4.7
70	ASFAAYAALLAAK	78	633.8635	2	1265.7131	15.16	-0.4
71	TAFAEYWNALAAK	76	727.8732	2	1453.7354	15.23	-2.5
72	ASFAAYWNALAAK	76	691.8661	2	1381.7141	16	-0.1
73	TSFAAYWAALAPK	74	698.3796	2	1394.7346	13.76	0.4

Identified sequences from MDM2 affinity selection condition B3. (ALC >70)
RT = retention time.

3.4 Affinity selection with 12ca5

3.4.1 Selection conditions

Condition	12ca5	Library	per member[2]
**A**	0.7 nmol,100 μg	1 μg	2 ng
**B**	0.7 nmol,100 μg	10 μg	20 ng
**C**	0.7 nmol,100 μg	50 μg	100 ng

12ca5 affinity selection condition. [a]Library was incubated with 12ca5 in 1x PBS (total volume: 100 μL) for 30 min in 4 °C before the HPSEC-based affinity selection. [b]Amount of each library member: amount of library divides by library diversity.

3.4.2 Identified sequences from condition A

Entry	Sequence	ALC (%)	*m/z*	z	Mass	RT (min)	Mass error (ppm)
1	AAADVPDYAAK	98	545.784	2	1089.545	9.87	4
2	AAYDAPDYAAK	99	577.7808	2	1153.54	9.29	7.8
3	APADVPDYAAK	96	558.7908	2	1115.561	9.78	4.5
4	YPADAPDYAAK	87	590.7881	2	1179.556	9.5	-0.6
5	YAADVADYAAK	99	578.7883	2	1155.556	11.01	8.9
6	YAYDAPDYAAK	96	623.7952	2	1245.567	10.2	7.3
7	YAADVPDYAAK	98	591.7977	2	1181.572	10.7	1.5
8	AAYDVADYAAK	97	578.7892	2	1155.556	10.31	-1
9	YPYDAPDYAAK	98	636.8034	2	1271.582	10.52	5.4
10	YPADVPDYAAK	99	604.8066	2	1207.587	11.06	2.4
11	APYDVADYAAK	98	591.7969	2	1181.572	10.48	6.5
12	YAYDVADYAAK	98	624.8032	2	1247.582	11.59	-2.3
13	YAYDVPDYAAK	99	637.812	2	1273.598	12.2	-1.4
14	YPYDVPDYAAK	96	650.8189	2	1299.614	12.03	1.4
15	AAADVPDYAAK	98	545.784	2	1089.545	9.88	4

Identified sequences from 12ca5 affinity selection condition A. (ALC > 80)
RT = retention time.

3.4.3 Identified sequences from condition B

Entry	Sequence	ALC (%)	*m/z*	z	Mass	RT (min)	Mass error (ppm)
1	AAADVADYAAK	98	532.7753	2	1063.53	8.14	0.7
2	YAADAPDYAAK	90	577.7821	2	1153.54	9.29	7.8
3	AAADVPDYAAK	94	545.7843	2	1089.545	9.59	5.4
4	APADVPDYAAK	96	558.7921	2	1115.561	9.78	4.5
5	YPADAPDYAAK	93	590.7892	2	1179.556	9.5	-0.6
6	YAADVADYAAK	98	578.79	2	1155.556	10.31	-1
7	YAYDAPDYAAK	97	623.7964	2	1245.567	10.2	7.3
8	AAYDVADYAAK	93	578.7907	2	1155.556	10.81	7.6
9	YPYDAPDYAAK	97	636.8035	2	1271.582	10.52	5.4
10	YPADVPDYAAK	87	604.8062	2	1207.587	11.36	2.5
11	AAYDVPDYAAK	92	591.7988	2	1181.572	10.48	6.5
12	YAYDVADYAAK	97	624.804	2	1247.582	11.43	7.4

13	YAYDVPDYAAK	96	637.8125	2	1273.598	12.06	4.5
14	YPYDVPDYAAK	96	650.8205	2	1299.614	11.86	3

Identified sequences from 12ca5 affinity selection condition B. (ALC > 80)

3.4.4 Identified sequences from condition C

Entry	Sequence	ALC (%)	*m/z*	z	Mass		
1	AAADVADYAAK	94	532.7751	2	1063.53	8.14	2.1
2	YAADAPDYAAK	96	577.7806	2	1153.54	9.07	0.7
3	AAADVPDYAAK	92	545.7846	2	1089.545	9.29	1.5
4	APADVPDYAAK	90	558.7915	2	1115.561	9.35	4.3
5	YPADAPDYAAK	93	590.7885	2	1179.556	9.4	0.9
6	YAADVADYAAK	94	578.7891	2	1155.556	10.21	-1.7
7	YAYDAPDYAAK	94	623.7961	2	1245.567	10.1	5
8	YPYDAPDYAAK	88	636.804	2	1271.582	10.46	-0.5
9	YPADVPDYAAK	85	604.8069	2	1207.587	10.83	1.9
10	YAYDVADYAAK	98	624.8035	2	1247.582	11.35	2.8
11	YAYDVPDYAAK	91	637.8116	2	1273.598	11.48	5.3
12	YPYDVPDYAAK	96	650.8194	2	1299.614	11.1	1.3

Identified sequences from 12ca5 affinity selection condition C. (ALC > 80)
RT = retention time.

3.5 Affinity selection against 14-3-3

3.5.1 Selection condition

Condition	14-3-3	Library	per member
**A**	11 nmol, 300 µg	900 µg	1753 ng

14-3-3 affinity selection condition. [a]Library was incubated with 14-3-3 in 1x PBS (total volume: 100 µL) for 30 min in 4 °C before the HPSEC-based affinity selection. [b]Amount of each library member: amount of library divides by library diversity.

3.5.2 Identified sequences

Entry	Peptide	ALC (%)	*m/z*	z	Mass	RT (min)	Mass error (ppm)
1	bbTAdAATeK	97	613.8123	2	1225.6106	9.35	2
2	AbTcdAATeK	96	594.2922	2	1186.5747	9.57	1.3
3	bAAcdeTTeK	96	654.8088	2	1307.5911	9.73	2

4	bbAAdeTTeK	96	674.3237	2	1346.627	9.76	1.8
5	AbTAdAATeK	95	572.7759	2	1143.5325	10.09	2.9
6	AbAAdeTTeK	93	633.2781	2	1264.5488	10.26	4.4
7	bAAAdAATeK	92	557.7689	2	1113.522	10.86	0.8
8	AbAcdeTTeK	92	654.8052	2	1307.5911	11.19	4.1
9	bATAdeTTeK	92	648.2841	2	1294.5593	11.31	2.3
10	bbTcdAATeK	92	635.3298	2	1268.6528	11.6	2.6
11	AbTAdeTTeK	92	648.2933	2	1294.5593	11.62	0.3
12	bAAAdeTAeK	92	618.2775	2	1234.5383	11.8	2.5
13	bbAcdeTTeK	92	695.8378	2	1389.6692	12.07	1.4
14	bAAcdAATeK	89	579.2857	2	1156.564	12.32	-0.4
15	bbTcdeTTeK	89	710.8444	2	1419.6799	12.37	-0.2
16	bATcdAATeK	88	396.5301	3	1186.5747	12.59	1.2
17	AbTcdeTTeK	87	669.8039	2	1337.6016	12.91	-0.1
18	bATcdeTAeK	86	654.8079	2	1307.5911	12.95	2
19	bbTAdeTTeK	86	689.3232	2	1376.6377	13.16	1.1
20	bAAAdeTTeK	85	633.2827	2	1264.5488	13.22	1.3
21	AATcdeTTeK	84	628.7672	2	1255.5234	13.4	0.9
22	AAAcdeTAeK	84	598.7556	2	1195.5022	13.93	0.4
23	bAAcdeTAeK	84	639.8054	2	1277.5806	14.17	0.5
24	AbAcdeTAeK	84	639.8019	2	1277.5806	14.19	3.7
25	bATAdATTeK	83	587.7764	2	1173.543	14.46	-0.8
26	AAAAdeTAeK	83	577.2344	2	1152.4602	14.63	4.8
27	bbAcdAATeK	83	620.3272	2	1238.6423	14.85	2.1
28	bbAcdeTAeK	83	680.8388	2	1359.6587	15.13	1.8
29	AAAAdeTTeK	82	592.2467	2	1182.4707	15.59	1.2
30	bbTAdAAAeK	82	598.8046	2	1195.6001	15.81	0.6

Identified sequences from 14-3-3 affinity selection condition A. (**ALC** > 80) (One letter code: b = Cha, T = β-Ser, c = Orn, d = pSer, e = Nph). Abbreviations: Cha, cyclohexyl alanine; β-Ser, beta-homoserine; Orn, ornithine; pSer, phosphoserine; Nph, 4-nitrophenylalanine. RT = retention time.

4. Supplementary Methods 3: Method of molecular docking

4.1 Preparation of structures

Besides alanine substitutions at residues 1, 2, 5, 8, 9, 11, and 12, peptide **34** also has an additional amino acid at the C-terminus (Lys13). To allow for separate evaluation of the effects of (1) the alanine substitutions and (2) the addition of Lys13 on MDM2 binding affinity, four peptides were prepared: PMI wild type (TSFAEYWNLLSP-NH_2),

PMI mutant (AAFAAYWAALAA-NH_2), PMI-K (TSFAEYWNLLSPK-NH_2), and peptide **34** (AAFAAYWAALAAK-NH_2). The crystal structure was obtained from RCSB (PDB: 3LNZ)[3]. Chain G was used as the model for MDM2. The one missing residue in the structure (Glu 25) was added and minimized. Hydrogens were added using UCSF Chimera (glutamate, aspartate, and lysine residues are charged)[4]. Chain H was used as the ligand with residue 8 mutated from an Ala to an Asn to restore the wild type sequence of PMI. For all four peptide structures, the C-terminus was capped with NH_2. Based on the PMI wild type, each mutated structure was created.

To create PMI-K, a Lys residue was added to the uncapped PMI and then capped with NH_2. The added region of the peptide was minimized while the first twelve residues and side chains were left fixed. All the aforementioned operations were carried out using UCSF Chimera[4]. PMI mutant AAFAAYWAALAA-NH_2 was created by mutating residues 1, 2, 5, 8, 9, 11, and 12 in PMI wild type to alanine. Peptide **34** was created by mutating residues 1, 2, 5, 8, 9, 11, and 12 of PMI-K to alanine. Note that the mutated structures were not minimized. The backbone ϕ, ψ angles of the first twelve residues on all four structures were the same as those of chain H in 3LNZ. Hydrogens were added to all peptide structures using UCSF Chimera (the N-termini and any Glu, Asp, and Lys side chains were charged).

4.2 Docking Experiment

AutoDock Vina was used to conduct the docking experiment.[5] The inputs were generated using AutoDock Tools. For the MDM2 protein, Kollman charges were added, and the backbone and side chains remained rigid. For the peptide ligands, the backbones were kept rigid and the side chain torsion angles were flexible. To ensure that starting position of docking did not match the crystal structure, the MDM2 protein was moved from the crystal structure position by 10 Å in each of the *x*, *y*, and *z* directions. The search box was set to 30 Å by 30 Å by 30 Å centered at the coordinates of $C\alpha$ of residue 6 from chain H in the crystal structure (shifted by 10 Å in each direction to match the shift performed in the MDM2 coordinates).

4.3 Docking result

Simultaneous Ala substitutions of all 7 non-hot spot residues (residues 1, 2, 5, 8, 9, 11, and 12) improves the calculated binding affinity with a $\Delta\Delta G$ of –3.4 kcal/mol for PMI and –2.5 kcal/mol for PMI-K. The addition of Lys impairs the binding affinity by 0.4 kcal/mol for PMI wild type and by 1.3 kcal/mol for PMI mutant.

5. Supplementary Note 2: Peptide characterization and validation

5.1 (i, i+4) alanine-substituted peptides

Peptide 1

Sequence: ASFAAYWNLLSP-*CONH₂*

HPLC: method A

HRMS (ESI-QTOF): Calcd. for $(M + 2H)^{2+}$: 669.843, found: 669.844. ppm=1.5

Total ion chromatogram:

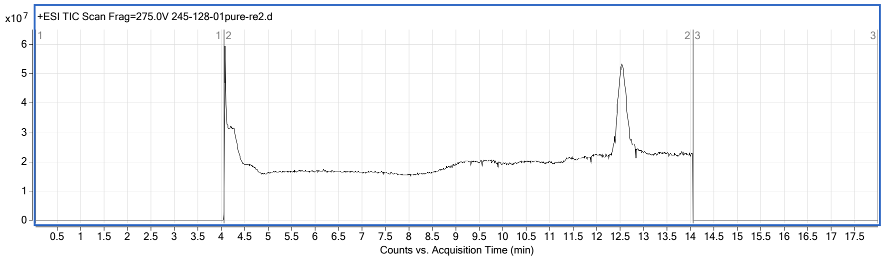

Mass spectrum of major compound

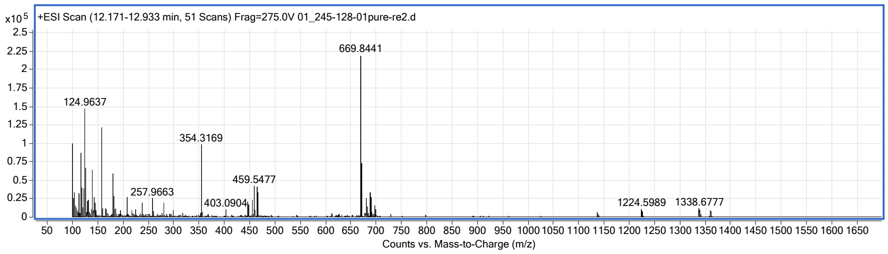

Peptide 2

Sequence: TAFAEAWNLLSP-*CONH₂*

HPLC: method B

HRMS (ESI-QTOF): Calcd. for $(M + H)^+$: 1318.476, found: 1318.474. ppm=1.5

Total ion chromatogram:

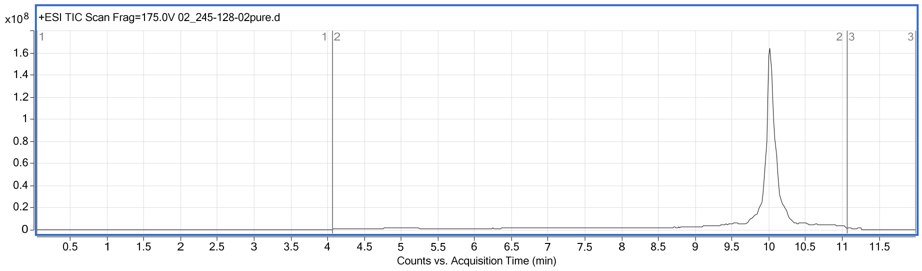

Mass spectrum of major compound

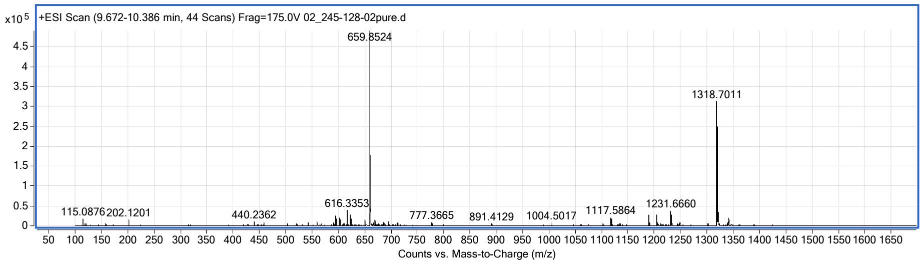

Peptide 3

Sequence: TSAAEYANLLSP-*CONH₂*

HPLC: method B

HRMS (ESI-QTOF): Calcd. for $(M + H)^{+1}$: 1235.648, found: 1235.647. ppm=1.0

Total ion chromatogram:

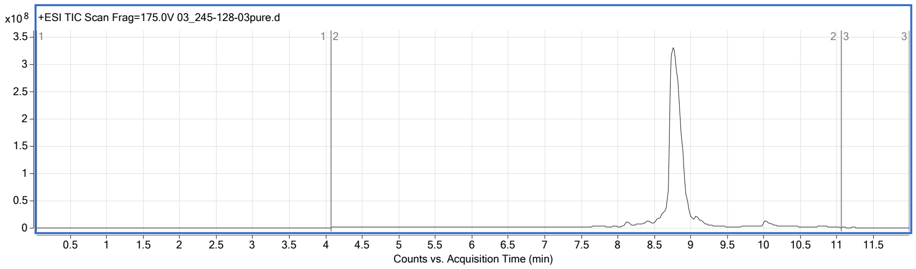

Mass spectrum of major compound

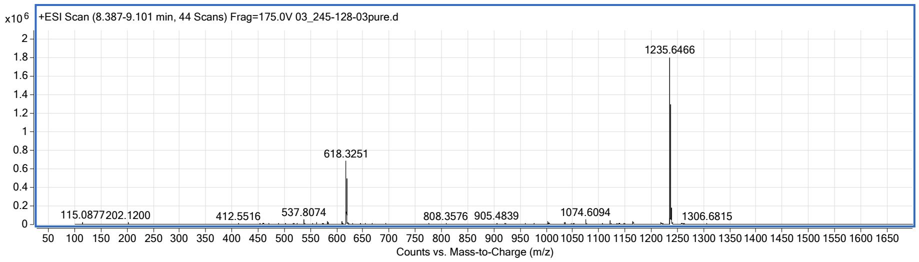

Peptide 4

Sequence: TSFAEYWALLSP-*CONH₂*

HPLC: method B

HRMS (ESI-QTOF): Calcd. for $(M + 2H)^{2+}$: 692.368, found: 692.363. ppm=7.2

Total ion chromatogram:

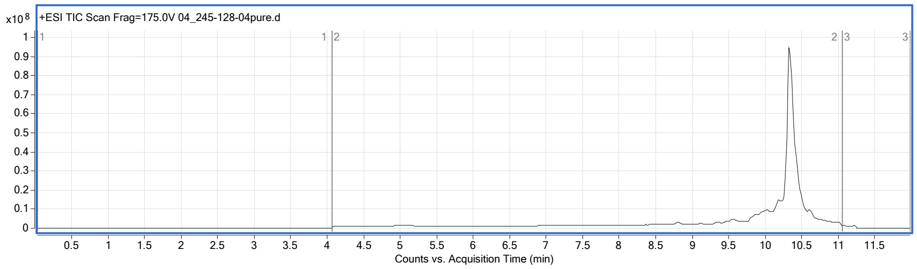

Mass spectrum of major compound

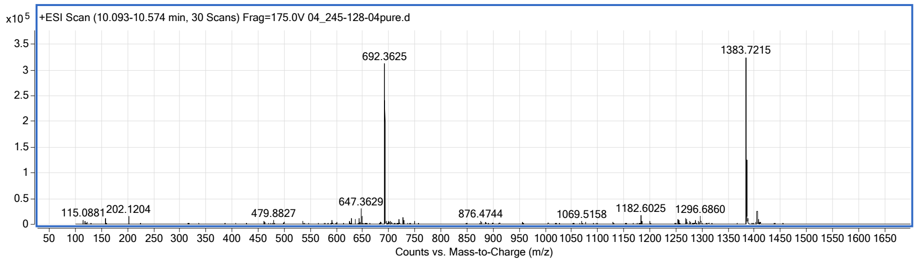

Peptide 5

Sequence: TSFAAYWNALSP-*CONH₂*

HPLC: method B

HRMS (ESI-QTOF): Calcd. for $(M + H)^{+}$: 1326.649, found: 1326.656. ppm=5.2

Total ion chromatogram:

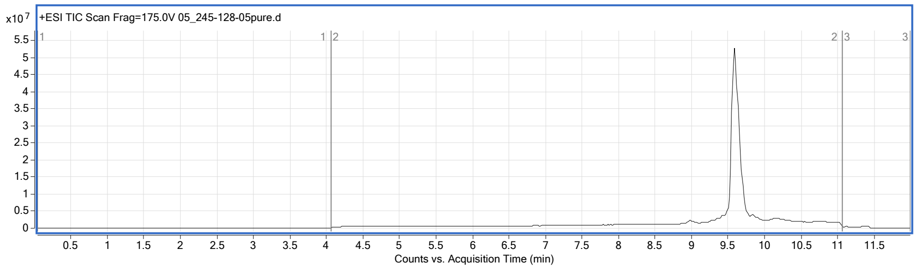

Mass spectrum of major compound

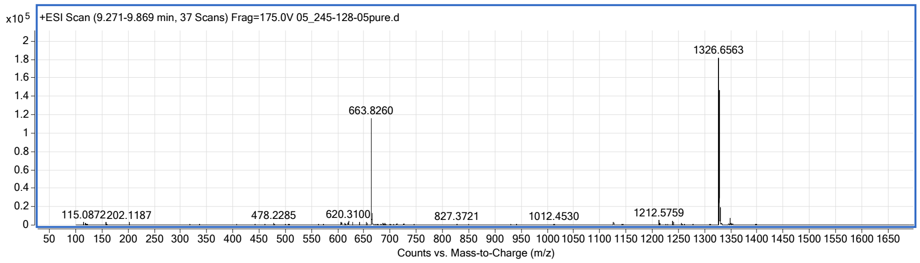

Peptide 6

Sequence: TSFAEAWNLASP-*CONH₂*

HPLC: method A

HRMS (ESI-QTOF): Calcd. for $(M + 2H)^{2+}$: 646.805, found: 646.814. ppm=13

Total ion chromatogram:

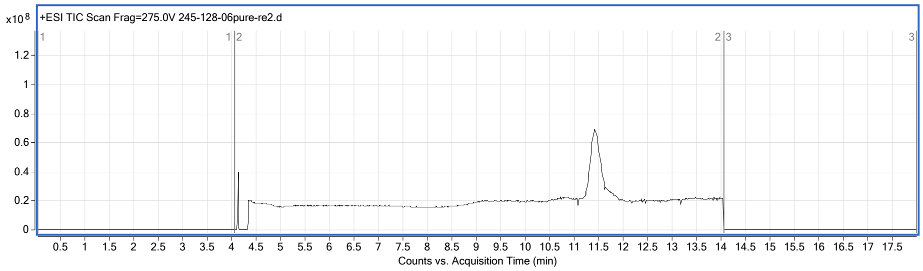

Mass spectrum of major compound

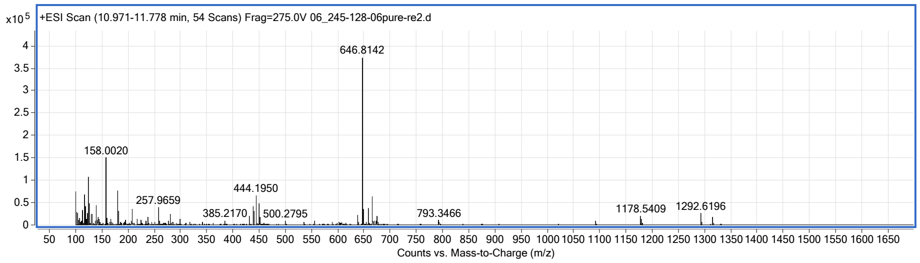

Peptide 7

Sequence: TSFAEYANLLAP-*CONH₂*

HPLC: method B

HRMS (ESI-QTOF): Calcd. for $(M + H)^{+}$: 1295.675, found: 1295.679. ppm=1.5

Total ion chromatogram:

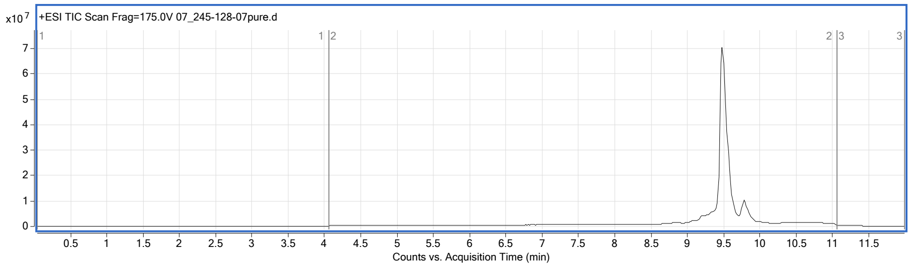

Mass spectrum of major compound

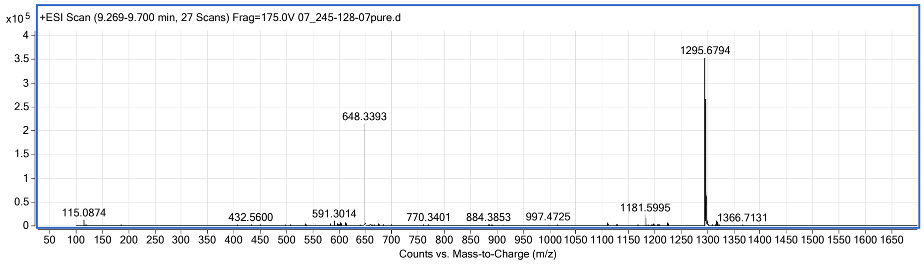

Peptide 8

Sequence: TSFAEYWALLSA-*CONH₂*

HPLC: method A

HRMS (ESI-QTOF): Calcd. for $(M + H)^+$: 1357.680, found: 1357.681. ppm=1.0

Total ion chromatogram:

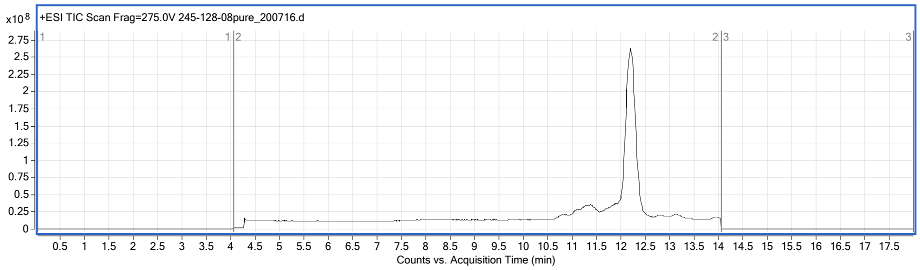

Mass spectrum of major compound

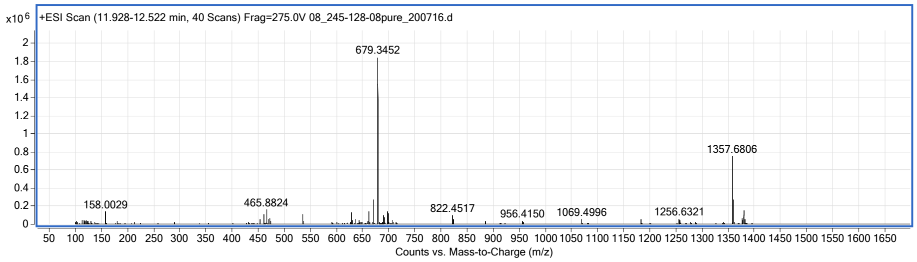

5.2 (i, i+4) perfluoroaryl stapled peptides

Peptide 9

Sequence: C*SFAC*YWNLLSP-*CONH₂* (C*: hexafluorobenzene-stapled cysteine)

HPLC: method C

HRMS (ESI-QTOF): Calcd. for $(M + 2H)^{2+}$: 774.804, found: 774.808. ppm=5.1

Total ion chromatogram:

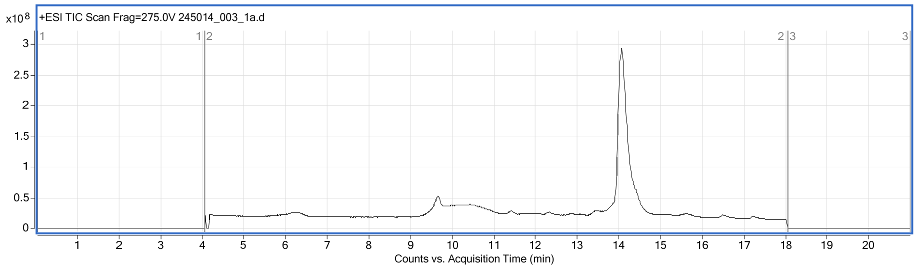

Mass spectrum of major compound

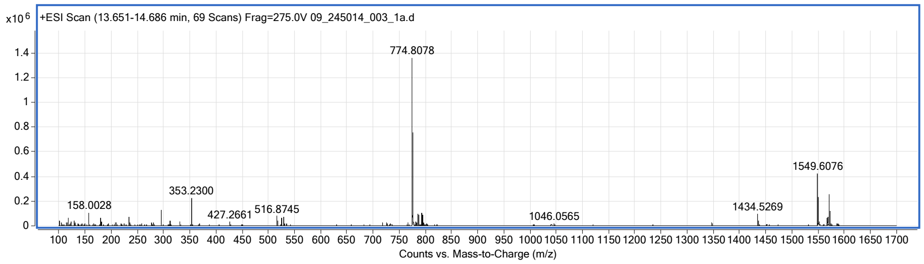

Peptide 10

Sequence: TC*FAEC*WNLLSP-*CONH₂* (C*: hexafluorobenzene-stapled cysteine)

HPLC: method C

HRMS (ESI-QTOF): Calcd. for $(M + H)^+$: 1528.583, found: 1528.601. ppm=12

Total ion chromatogram:

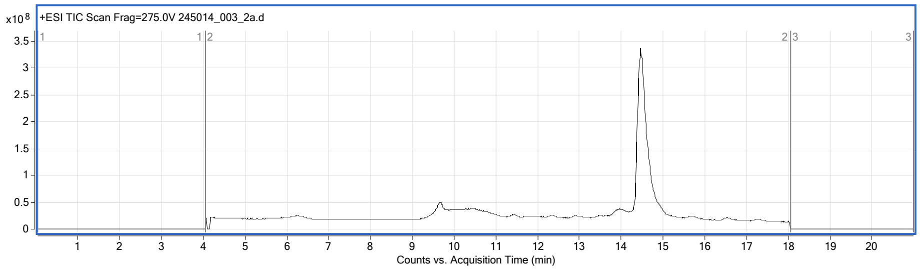

Mass spectrum of major compound

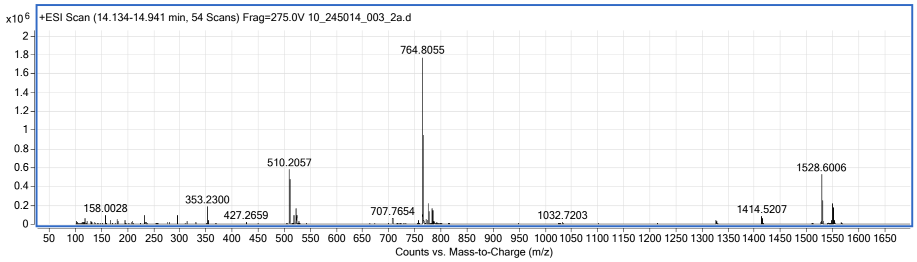

Peptide 11

Sequence: TSC*AEYC*NLLSP-*CONH₂* (C*: hexafluorobenzene-stapled cysteine)

HPLC: method C

HRMS (ESI-QTOF): Calcd. for $(M + H)^+$: 1445.531, found: 1445.548. ppm=11

Total ion chromatogram:

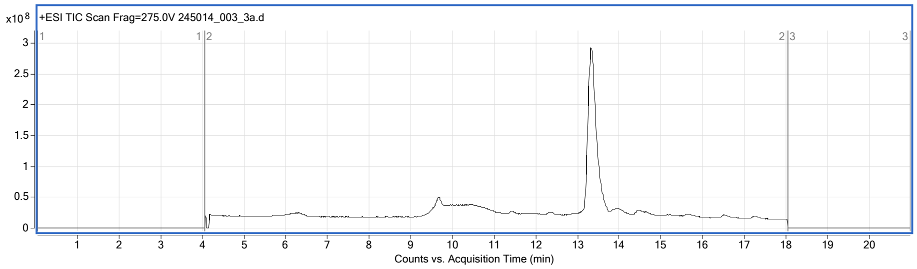

Mass spectrum of major compound

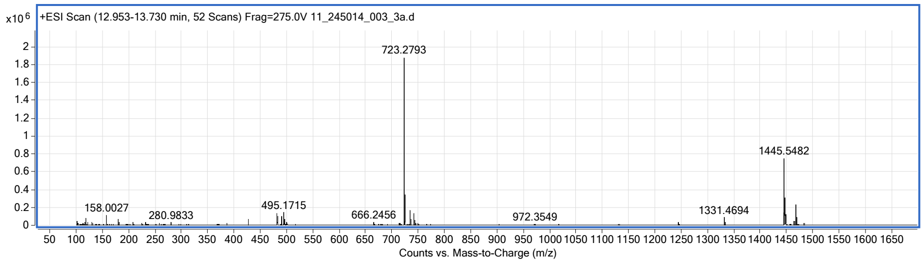

Peptide 12

Sequence: TSFC*EYWC*LLSP-*CONH₂* (C*: hexafluorobenzene-stapled cysteine)

HPLC: method C

HRMS (ESI-QTOF): Calcd. for $(M + 2H)^{2+}$: 797.299, found: 797.313. ppm=17

Total ion chromatogram:

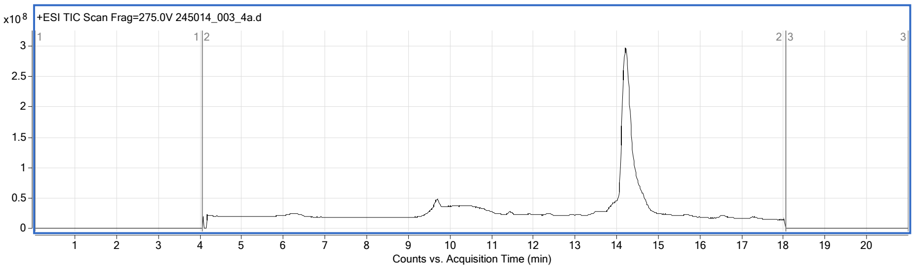

Mass spectrum of major compound

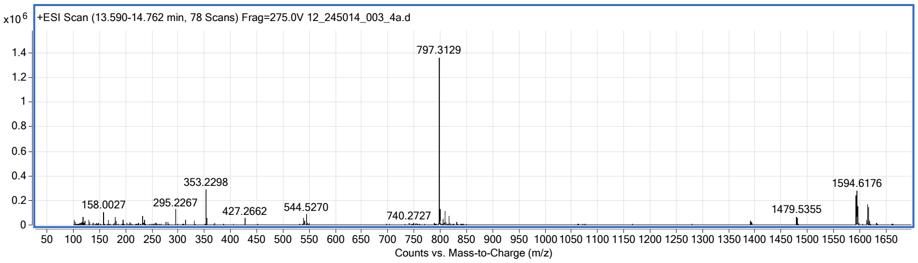

Peptide 13

Sequence: TSFAC*YWNC*LSP-*CONH₂* (C*: hexafluorobenzene-stapled cysteine)

HPLC: method C

HRMS (ESI-QTOF): Calcd. for $(M + 2H)^{2+}$: 768.786, found: 768.790. ppm=5.2

Total ion chromatogram:

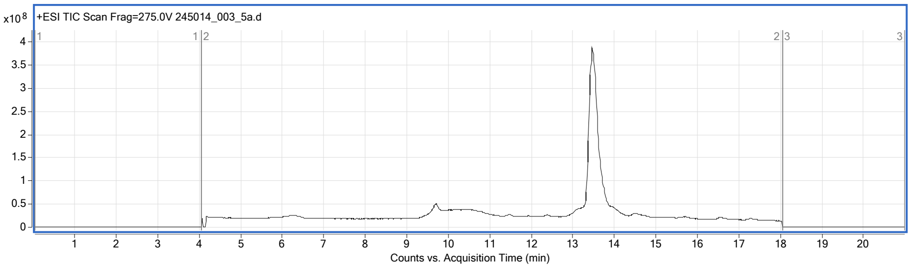

Mass spectrum of major compound

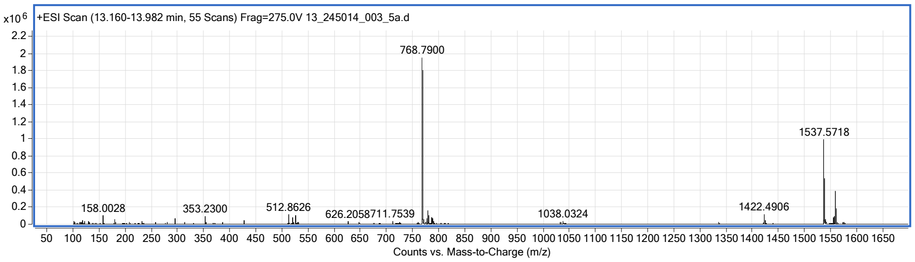

Peptide 14

Sequence: TSFAEC*WNLC*SP-*CONH₂* (C*: hexafluorobenzene-stapled cysteine)

HPLC: Method C

HRMS (ESI-QTOF): Calcd. for $(M + 2H)^{2+}$: 751.766, found: 751.779. ppm=17

Total ion chromatogram:

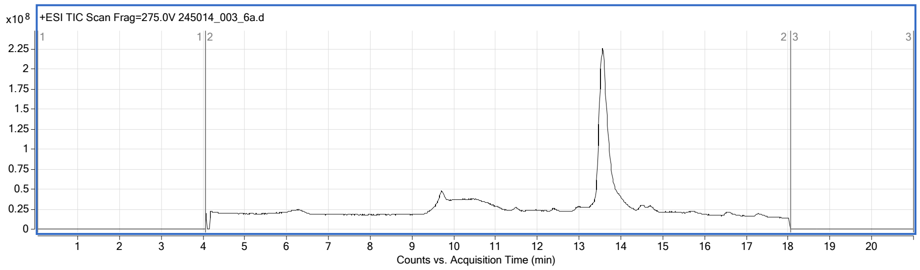

Mass spectrum of major compound

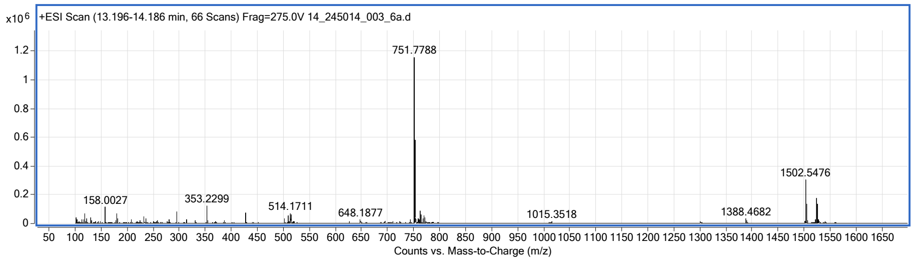

Peptide 15

Sequence: TSFAEYC*NLLC*P-*CONH₂* (C*: hexafluorobenzene-stapled cysteine)

HPLC: Method C

HRMS (ESI-QTOF): Calcd. for $(M + H)^{+}$: 1505.567, found: 1505.585. ppm=12

Total ion chromatogram:

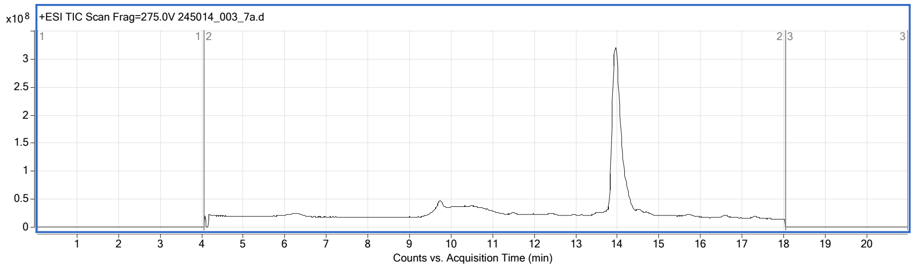

Mass spectrum of major compound

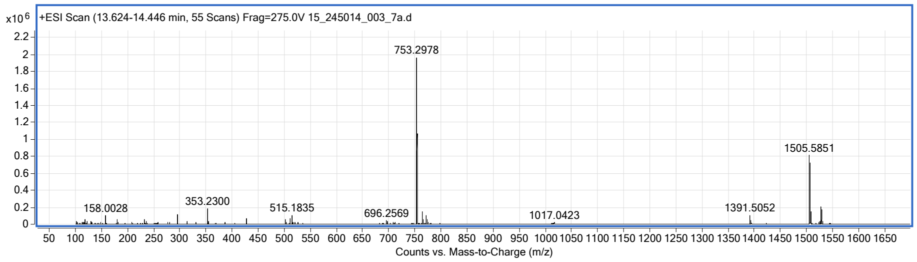

Peptide 16

Sequence: TSFAEYWC*LLSC*-*CONH₂* (C*: hexafluorobenzene-stapled cysteine)

HPLC: Method C

HRMS (ESI-QTOF): Calcd. for $(M + H)^+$: 1567.583, found: 1567.599. ppm=10

Total ion chromatogram:

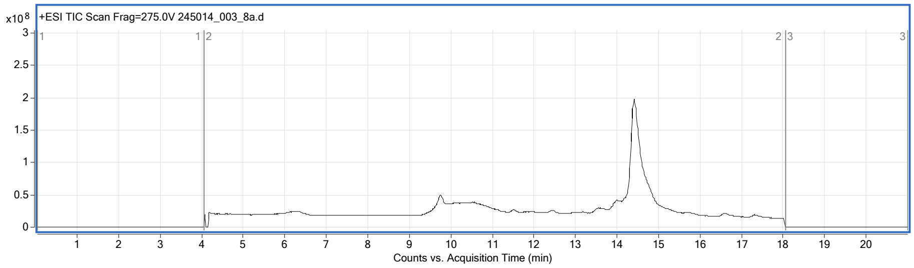

Mass spectrum of major compound

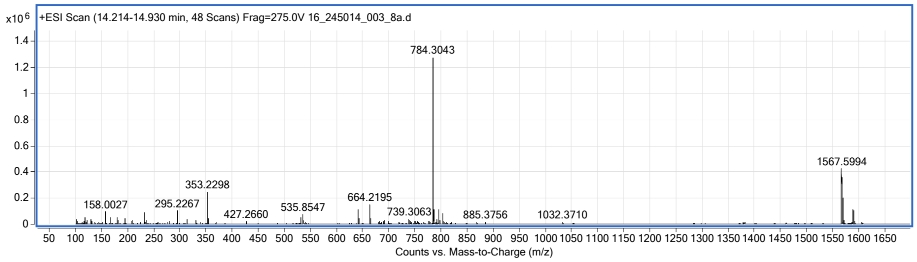

5.3 Multi-alanine-substituted peptides

Peptide 17

Sequence: TSFAEYWAALSPK-*CONH₂*

HPLC: Method B

HRMS (ESI-QTOF): Calcd. for $(M + H)^+$: 1469.724, found: 1469.737.ppm=8.8

Total ion chromatogram:

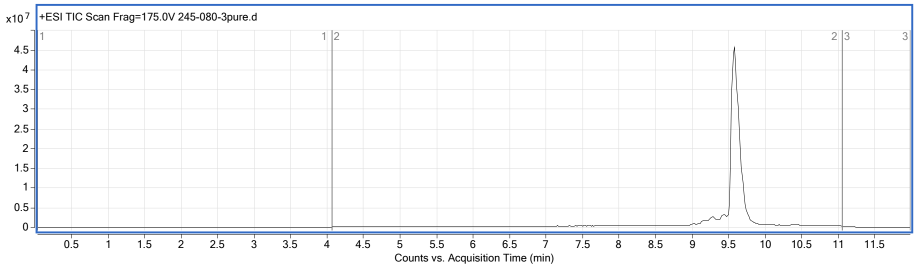

Mass spectrum of major compound

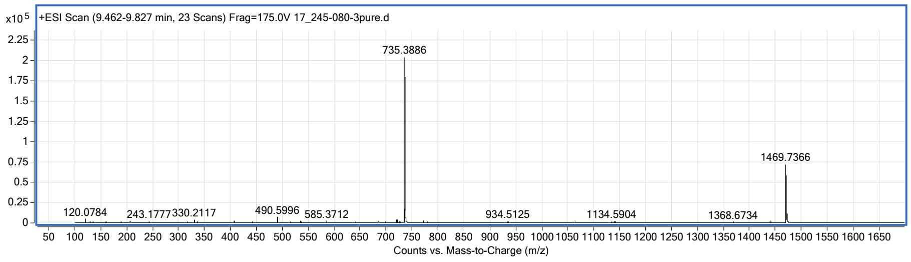

Peptide 18

Sequence: TSFAEYWNALSAK-*CONH₂*

HPLC: Method B

HRMS (ESI-QTOF): Calcd. for $(M + H)^{+}$: 1486.784, found: 1486.799. ppm=10

Total ion chromatogram:

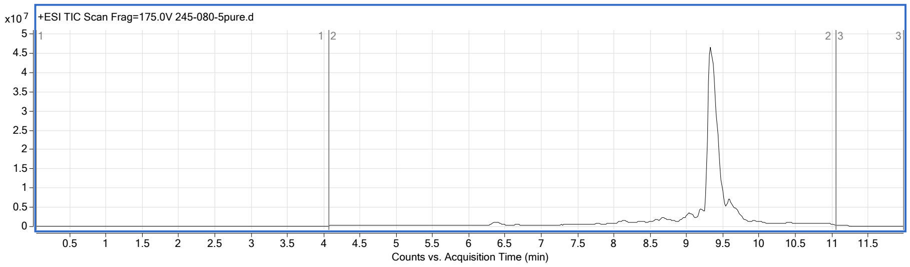

Mass spectrum of major compound

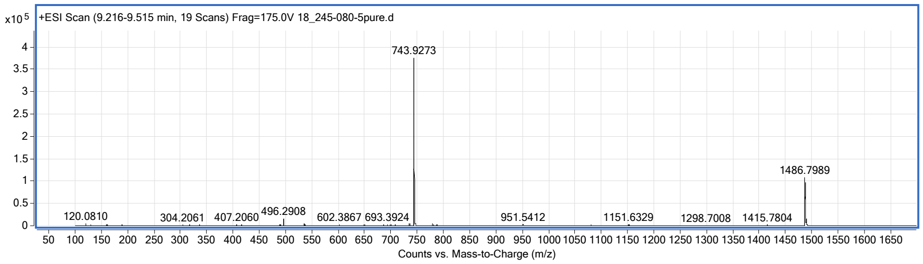

Peptide 19

Sequence: TSFAEYWNALAPK-*CONH₂*

HPLC: Method B

HRMS (ESI-QTOF): Calcd. for $(M + H)^{+}$: 1496.835, found: 1496.847. ppm=8

Total ion chromatogram:

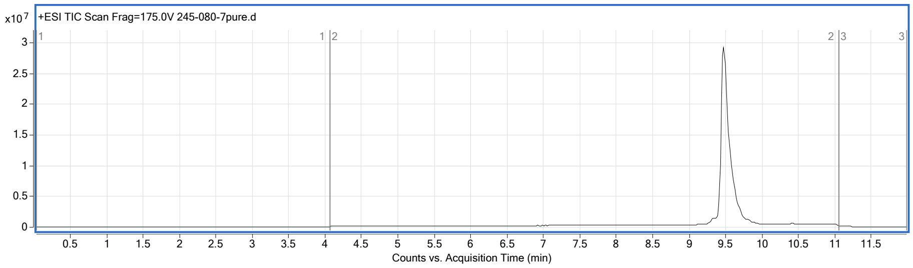

Mass spectrum of major compound

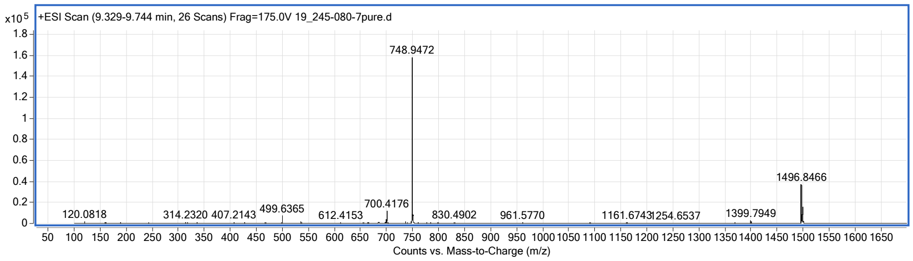

Peptide 20

Sequence: TSFAEYWNALAAK-*CONH₂*

HPLC: Method B

HRMS (ESI-QTOF): Calcd. for $(M + 2H)^{2+}$: 735.860, found: 735.856. ppm=5.4

Total ion chromatogram:

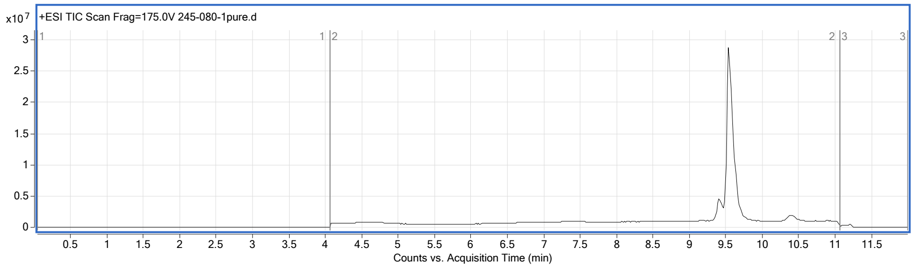

Mass spectrum of major compound

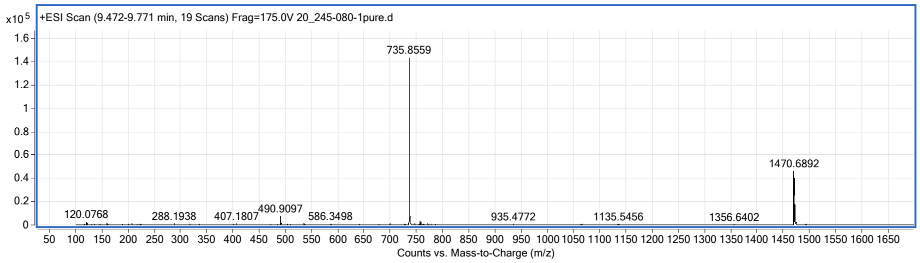

Peptide 21

Sequence: ASFAEYWAALSPK-*CONH₂*

HPLC: Method B

HRMS (ESI-QTOF): Calcd. for $(M + H)^{+}$: 1439.713, found: 1439.7109. ppm=2

Total ion chromatogram:

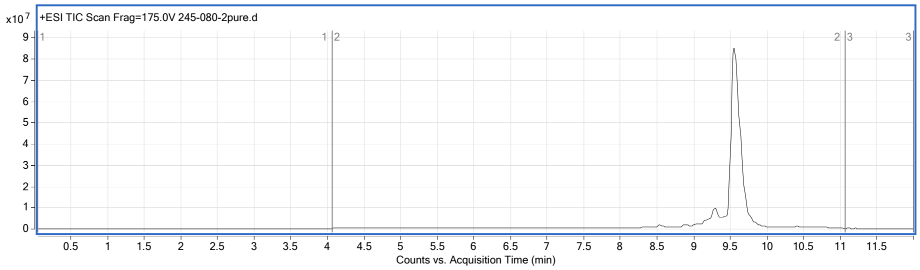

Mass spectrum of major compound

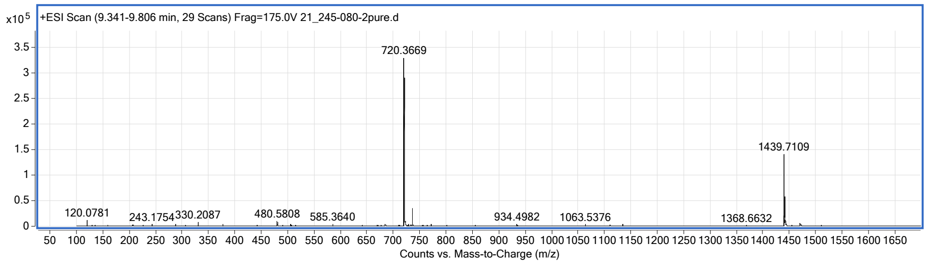

Peptide 22

Sequence: TSFAEYWAALSAK-*CONH₂*

HPLC: Method B

HRMS (ESI-QTOF): Calcd. for $(M + 2H)^{2+}$: 722.344, found: 722.334. ppm=13

Total ion chromatogram:

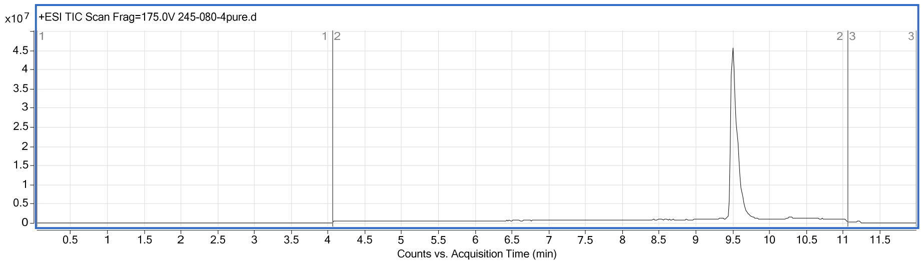

Mass spectrum of major compound

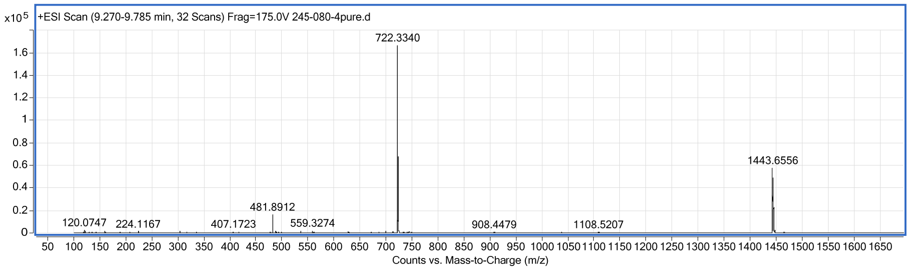

Peptide 23

Sequence: TAFAEYWNALSAK-*CONH₂*

HPLC: Method B

HRMS (ESI-QTOF): Calcd. for $(M + H)^{+}$: 1470.819, found: 1470.829. ppm=6.8

Total ion chromatogram:

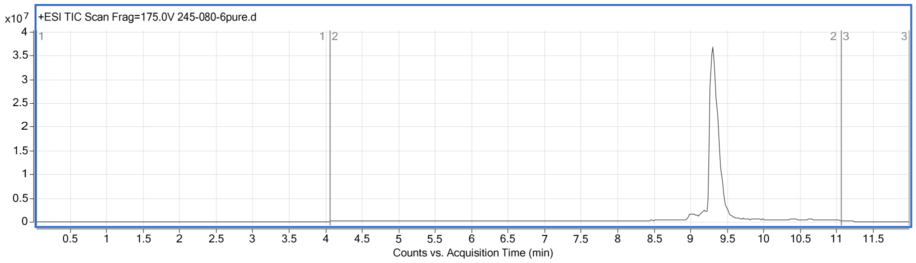

Mass spectrum of major compound

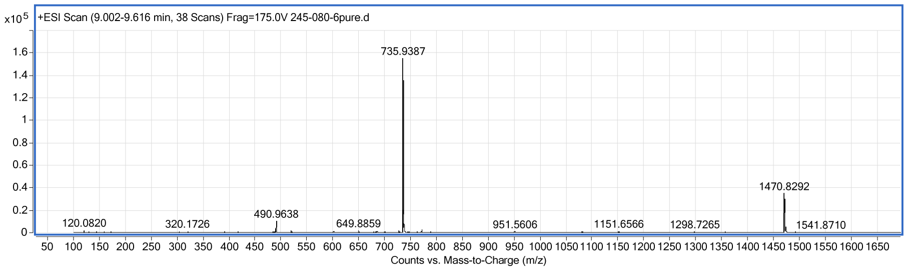

Peptide 24

Sequence: ASFAEYWAALAPK-*CONH₂*

HPLC: Method B

HRMS (ESI-QTOF): Calcd. for $(M + H)^+$: 1423.719, found: 1423.755. ppm=12

Total ion chromatogram:

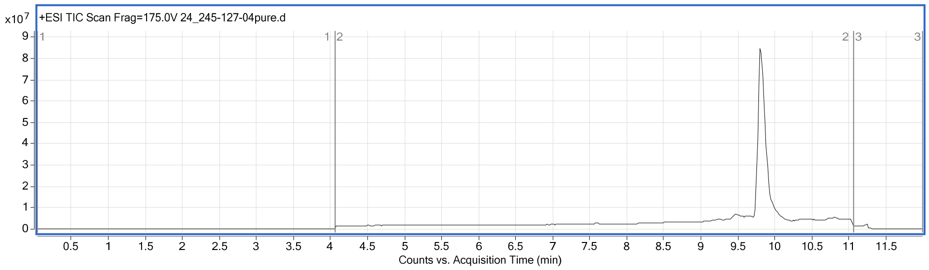

Mass spectrum of major compound

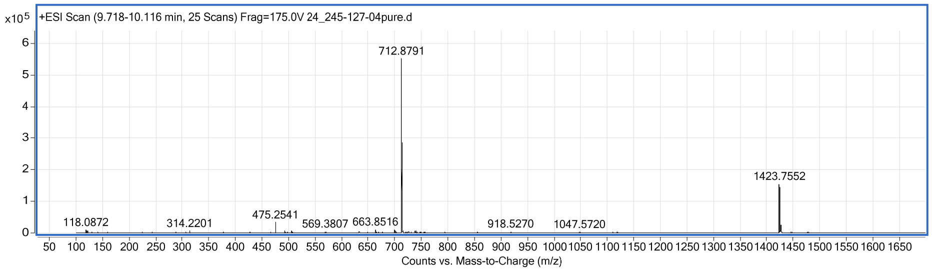

Peptide 25

Sequence: TSFAAYWAALSAK-*CONH₂*

HPLC: Method C

HRMS (ESI-QTOF): Calcd. for $(M + H)^+$: 1385.713, found: 1385.723. ppm=10

Total ion chromatogram:

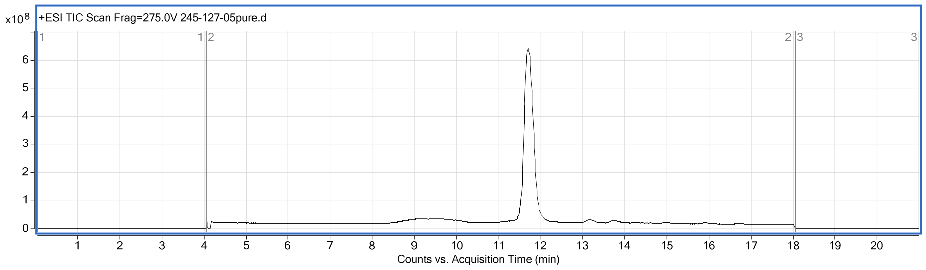

Mass spectrum of major compound

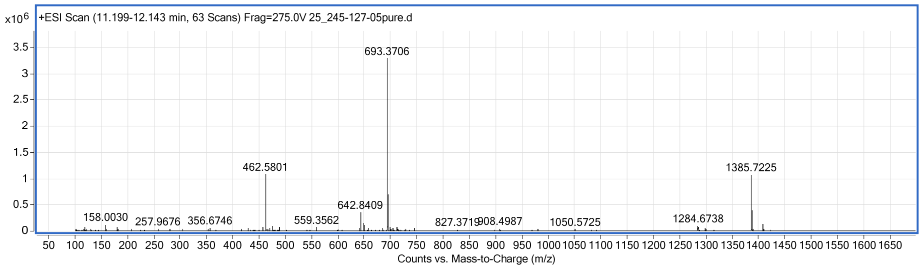

Peptide 26

Sequence: TAFAEYWAALSAK-*CONH₂*

HPLC: Method C

HRMS (ESI-QTOF): Calcd. for $(M + H)^+$: 1427.723, found: 1427.733. ppm=7

Total ion chromatogram:

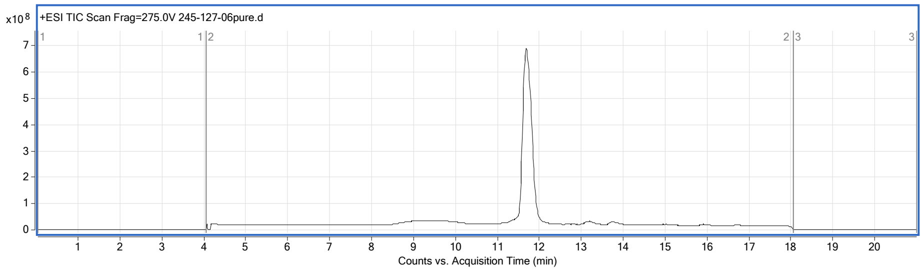

Mass spectrum of major compound

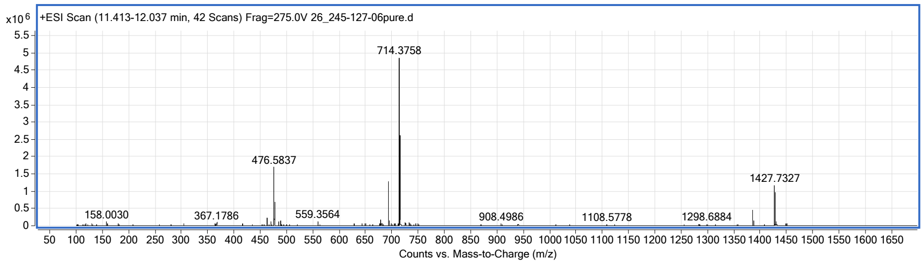

Peptide 27

Sequence: AAFAEYWAALSPK-*CONH₂*

HPLC: Method B

HRMS (ESI-QTOF): Calcd. for $(M + H)^+$: 1423.759, found: 1423.759. ppm=1

Total ion chromatogram:

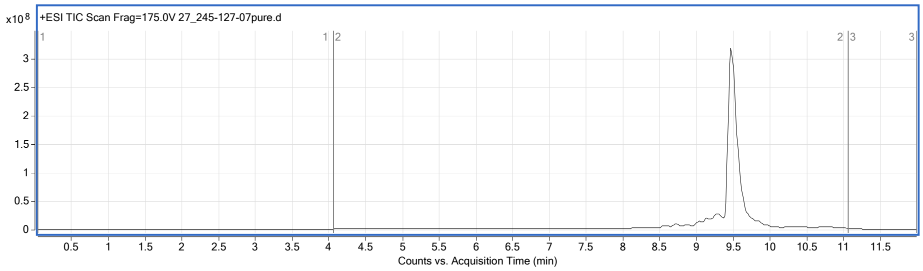

Mass spectrum of major compound

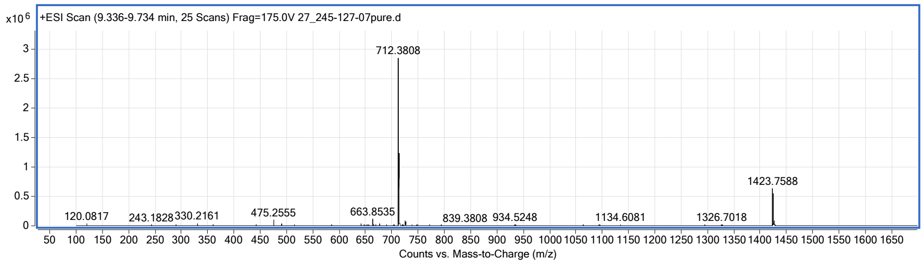

Peptide 28

Sequence: ASFAEYWNALAAK-*CONH₂*

HPLC: Method B

HRMS (ESI-QTOF): Calcd. for $(M + H)^+$: 1440.759, found: 1440.750. ppm=6

Total ion chromatogram:

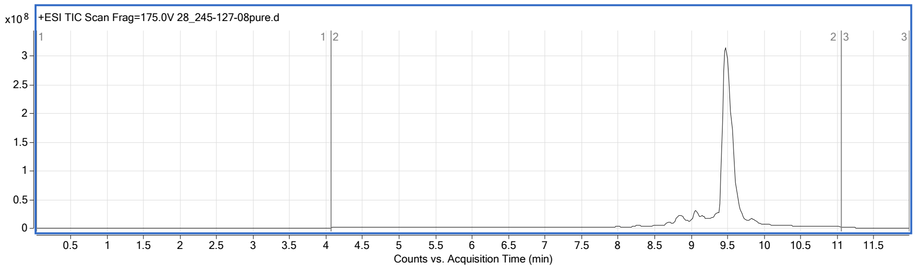

Mass spectrum of major compound

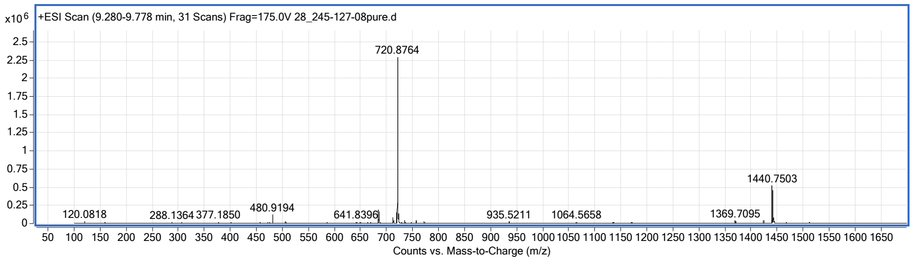

Peptide 29

Sequence: ASFAEYWAALAAK-*CONH₂*

HPLC: Method C

HRMS (ESI-QTOF): Calcd. for $(M + H)^+$: 1397.703, found: 1397.722. ppm=13

Total ion chromatogram:

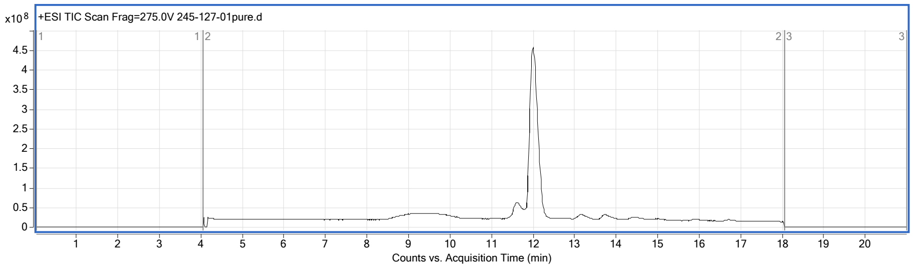

Mass spectrum of major compound

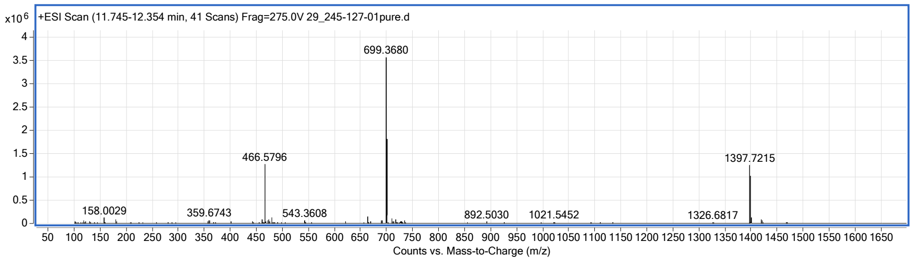

Peptide 30

Sequence: ASFAAYWAALSAK-*CONH₂*

HPLC: Method C

HRMS (ESI-QTOF): Calcd. for $(M + 2H)^{2+}$: 678.346, found: 678.363. ppm=12

Total ion chromatogram:

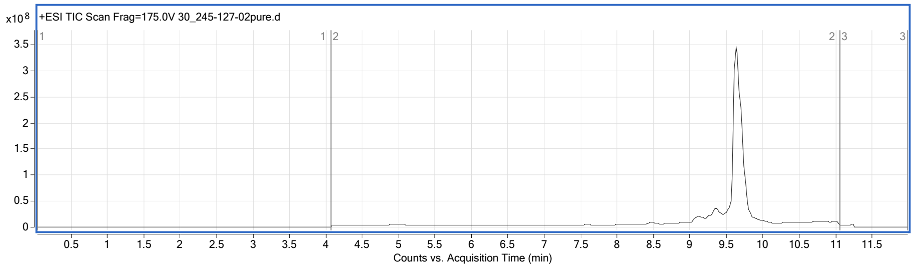

Mass spectrum of major compound

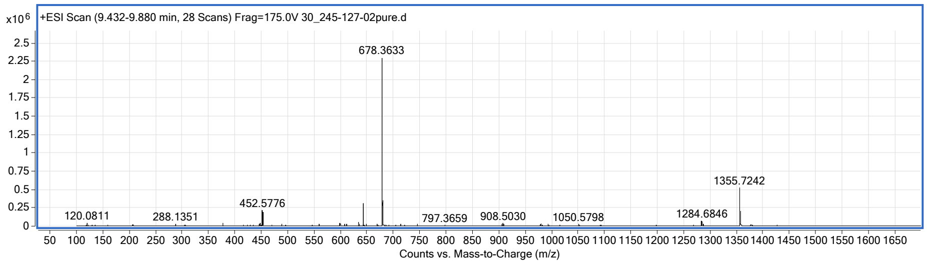

Peptide 31

Sequence: AAFAEYWNALAAK-*CONH₂*

HPLC: Method A

HRMS (ESI-QTOF): Calcd. for $(M + H)^{+}$: 1424.714, found: 1424.727. ppm=7.7

Total ion chromatogram:

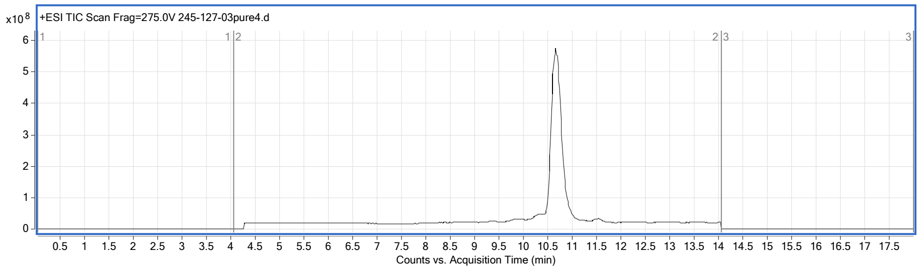

Mass spectrum of major compound

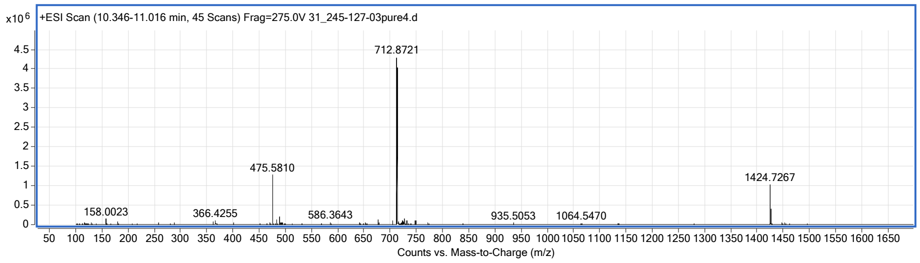

Peptide 32

Sequence: TAFAAYWAALAAK-*CONH₂*

HPLC: Method D

HRMS (ESI-QTOF): Calcd. for $(M + H)^+$: 1353.713, found: 1353.734. ppm=15

Total ion chromatogram:

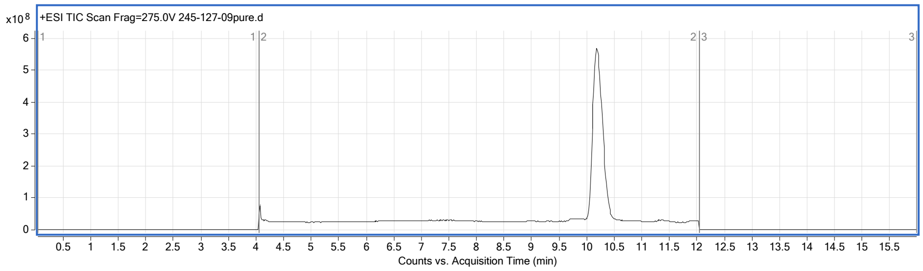

Mass spectrum of major compound

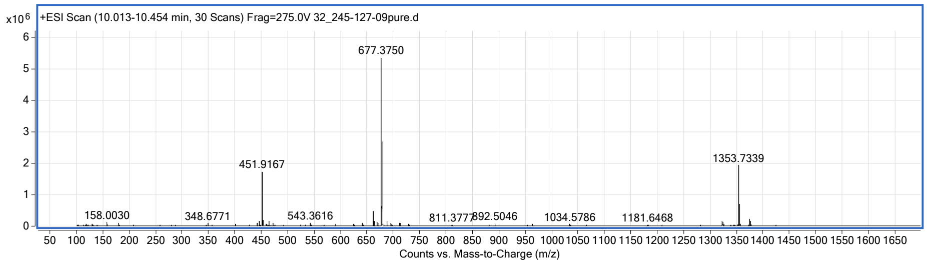

Peptide 33

Sequence: ASFAAYWAALAAK-*CONH₂*

HPLC: Method A

HRMS (ESI-QTOF): Calcd. for $(M + H)^+$: 1339.697, found: 1339.709. ppm=9.4

Total ion chromatogram:

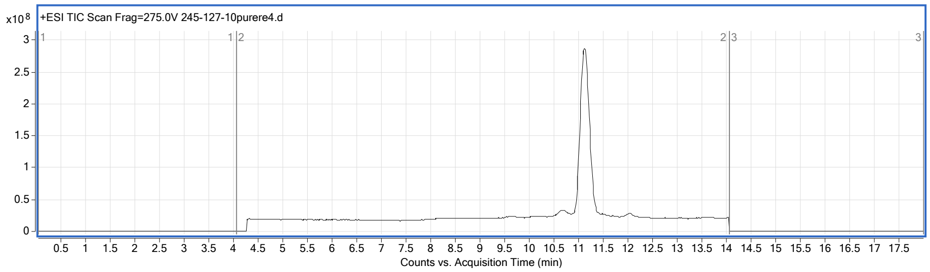

Mass spectrum of major compound

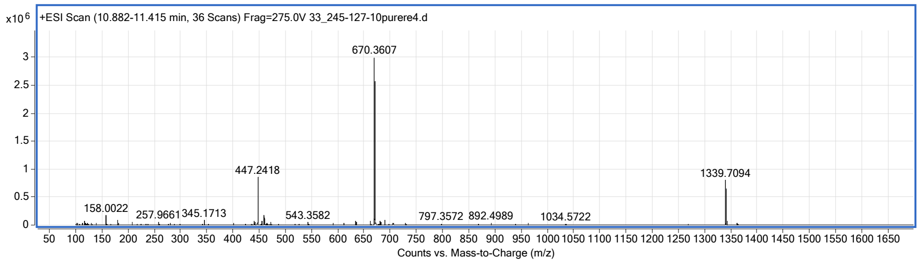

Peptide 34

Sequence: AAFAAYWAALAAK-*CONH₂*

HPLC: Method D

HRMS (ESI-QTOF): Calcd. for $(M + H)^+$: 1323.702, found: 1323.723. ppm=13

Total ion chromatogram:

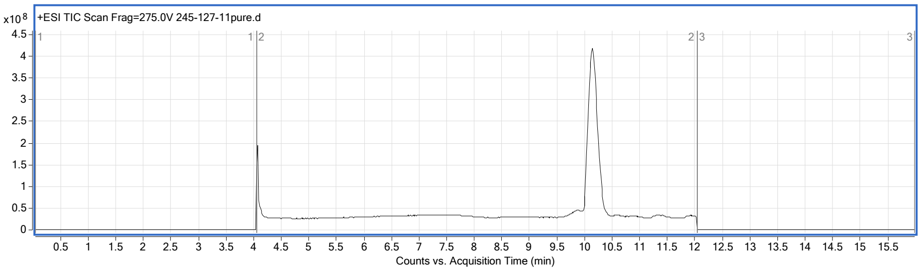

Mass spectrum of major compound

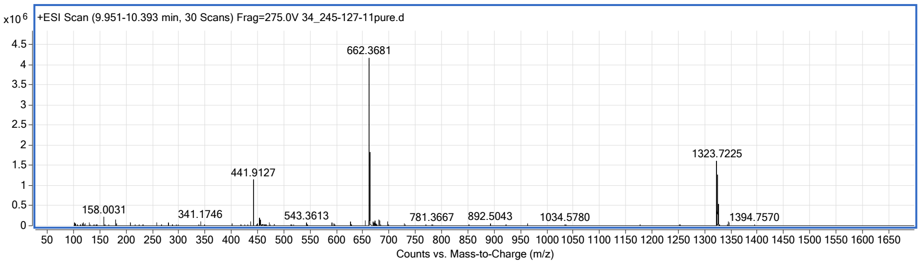

5.4 Multi-alanine-substituted stapled peptides

Peptide 35

Sequence: AAFC*EYWC*ALSP-*CONH₂* (C*: hexafluorobenzene-stapled cysteine)

HPLC: Method E

HRMS (ESI-QTOF): Calcd. for $(M + H)^+$: 1505.546, found: 1505.567. ppm=15

Total ion chromatogram:

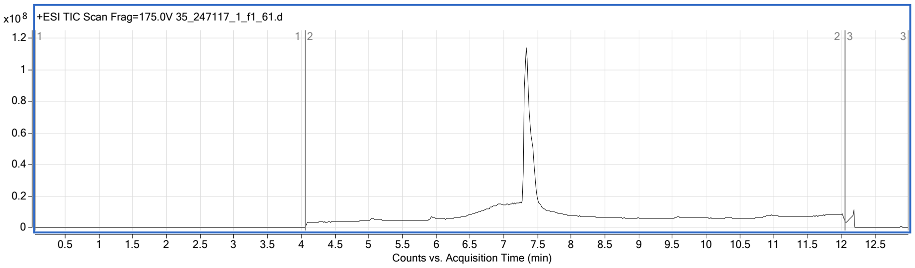

Mass spectrum of major compound

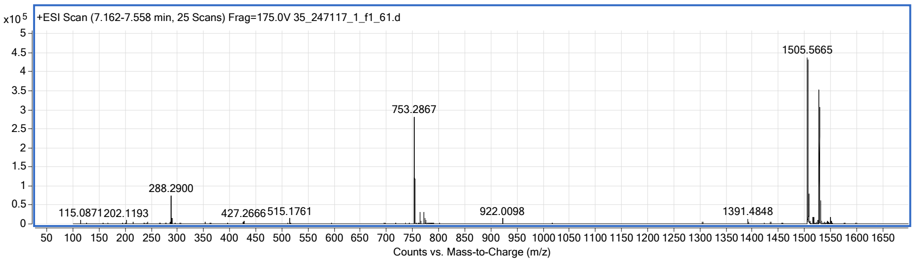

Peptide 36

Sequence: ASFC*EYWC*ALAA-*CONH₂* (C*: hexafluorobenzene-stapled cysteine)

HPLC: Method E

HRMS (ESI-QTOF): Calcd. for $(M + 2H)^{2+}$: 740.265, found: 740.279. ppm=11

Total ion chromatogram:

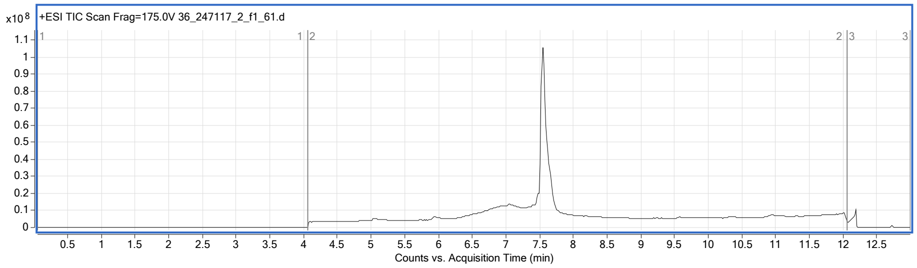

Mass spectrum of major compound

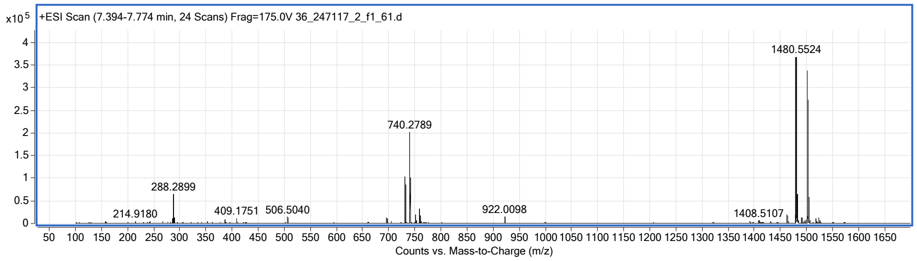

Peptide 37

Sequence: TSFAC*YWAC*LSA-*CONH₂* (C*: hexafluorobenzene-stapled cysteine)

HPLC: Method E

HRMS (ESI-QTOF): Calcd. for $(M + 2H)^{2+}$: 734.265, found: 734.279. ppm=14

Total ion chromatogram:

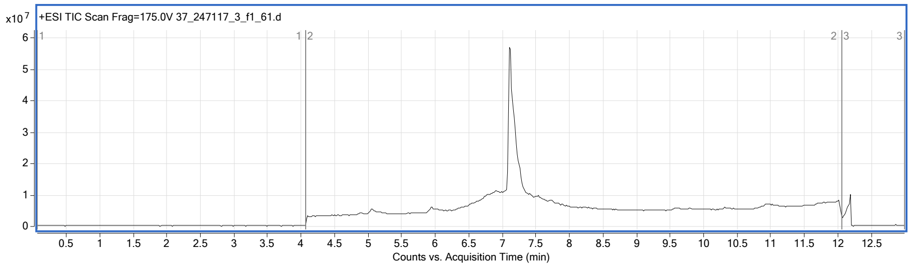

Mass spectrum of major compound

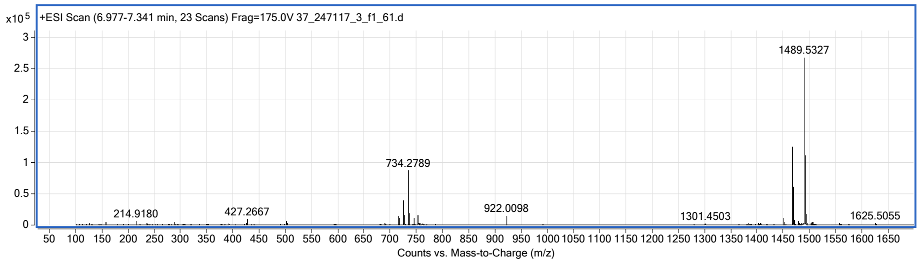

Peptide 38

Sequence: ASFAC*YWAC*LSA-*CONH₂* (C*: hexafluorobenzene-stapled cysteine)

HPLC: Method E

HRMS (ESI-QTOF): Calcd. for $(M + 2H)^{2+}$: 719.260, found: 719.274. ppm=15

Total ion chromatogram:

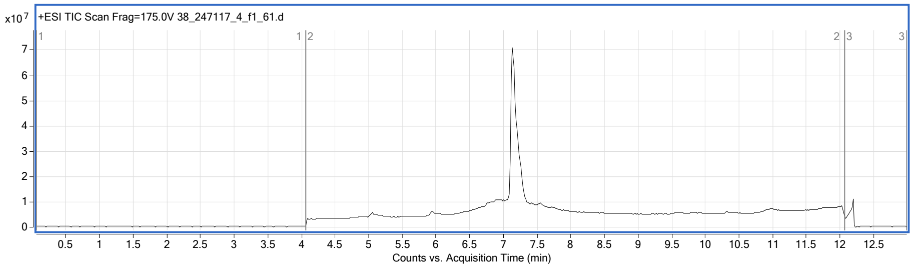

Mass spectrum of major compound

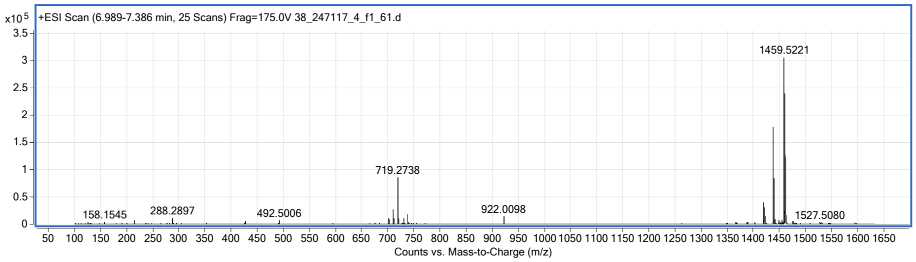

Peptide 39

Sequence: ASFAEYWC*ALAC*-*CONH₂* (C*: hexafluorobenzene-stapled cysteine)

HPLC: Method E

HRMS (ESI-QTOF): Calcd. for $(M + H)^{+}$: 1479.530, found: 1479.551. ppm=13

Total ion chromatogram:

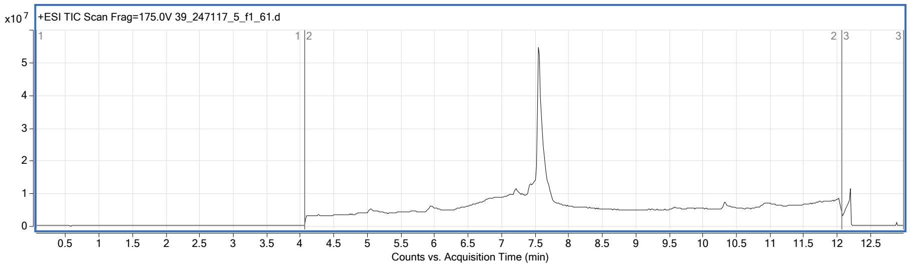

Mass spectrum of major compound

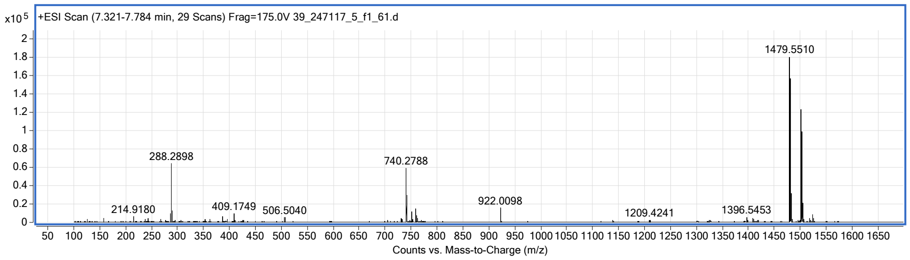

Peptide 40

Sequence: TSFAEYWC*ALSC*-*CONH₂* (C*: hexafluorobenzene-stapled cysteine)

HPLC: Method E

HRMS (ESI-QTOF): Calcd. for $(M + H)^+$: 1525.536, found: 1525.556. ppm=13

Total ion chromatogram:

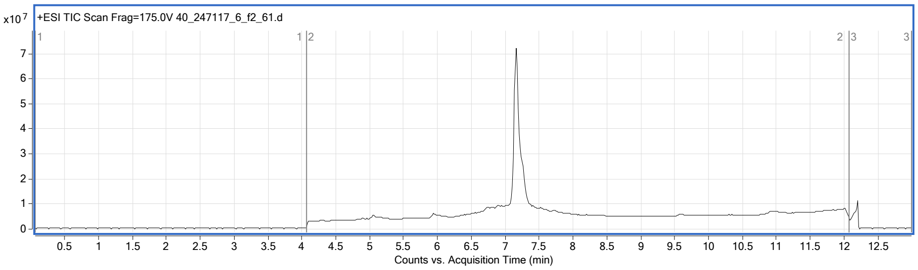

Mass spectrum of major compound

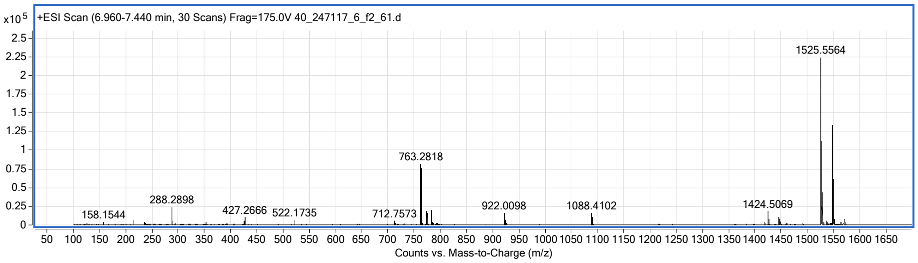

6. Supplementary Methods 4: Binding validation by bio-layer interferometry

6.1 Methods for BLI assay

In vitro binding assay was performed using ForteBio Octet RED96 BLI system (Octet RED96). In a typical experiment, the streptavidin (SA) tips were dipped in 200 µL biotinylated- peptide solution (2.0 µM in PBS with 0.02% Tween 20 and 1 mg/mL BSA) for the loading step. The loaded tips were then dipped in the sample wells for association curves. Buffer-only condition were used as reference for background subtraction. After association, the tips were dipped in blocking buffer (1x PBS, 0.02% Tween 20, 1 mg/mL BSA) to obtain dissociation curve. The association and dissociation curves were fitted by the global fitting algorithm, under binding model 1:1 to obtain the binding response value (R) in nm.

Streptavidin sensors were soaked in blocking buffer for 5 min. After immobilizing the PEG4-biotinylated peptide (100 nM of Biotin-PEG4-peptide) onto streptavidin sensors, serial dilutions of SUMO-$^{25\text{-}109}$MDM2 in blocking buffer were analyzed for binding (500 nM, 250 nM, 125 nM, 63nM, 32 nM, 16 nM, 8 nM and 4 nM. The response was recorded at equilibrium after 2 min.

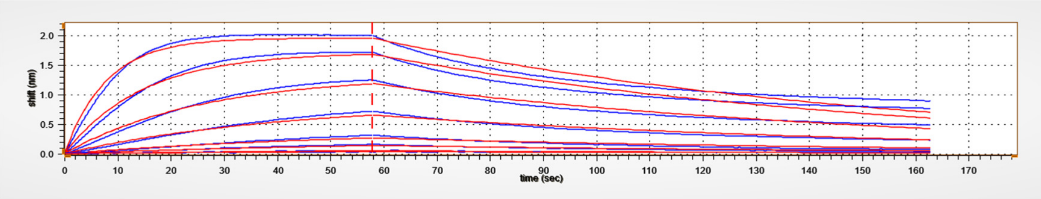

Compound name: **32-**biotin

Sequence: Biotin-PEG4-TAFAAYWAALAAK

k_{off} = 0.00981 (s^{-1}). k_{on} = 6.33E+05 (M^{-1} s^{-1}). K_D = 1.55E-08 (M)

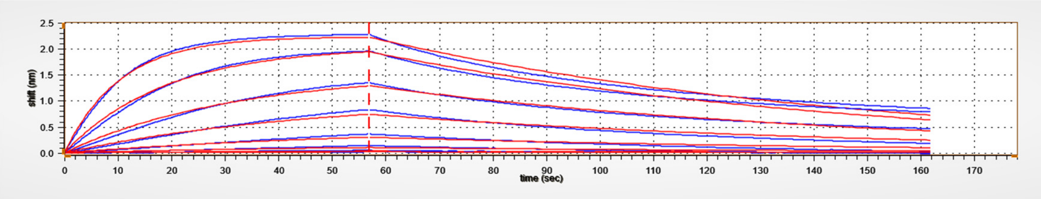

Compound name: **33-**biotin.

Sequence: Biotin-PEG4-ASFAAYWAALAAK

k_{off} = 0.0107 (s^{-1}). k_{on} = 1.70E+05 (M^{-1} s^{-1}). K_D = 6.28E-08 (M)

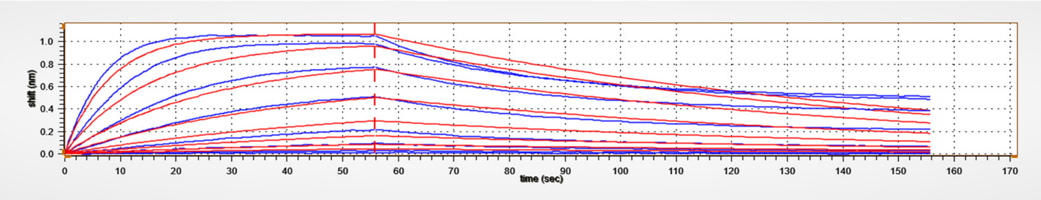

Compound name: **34-**biotin.

Sequence: Biotin-PEG4-AAFAAYWAALAAK

k_{off} = 0.0102 (s^{-1}). k_{on} = 8.24E+05(M^{-1} s^{-1}). K_D = 1.22E-08 (M)

6.2 Methods for BLI competition assay

In vitro binding assay was performed using ForteBio Octet RED96 BLI system (Octet RED96). In a typical experiment, the streptavidin (SA) tips were dipped in 200 µL biotinylated- peptide solution (2.0 µM in PBS with 0.02% Tween 20 and 1 mg/mL BSA) for the loading step. The loaded tips were then dipped in the sample wells for association curves. Buffer-only condition were used as reference for background subtraction. After association, the tips were dipped in blocking buffer (1x PBS, 0.02% Tween 20, 1 mg/mL BSA) to obtain dissociation curve. The association and dissociation curves were fitted by the global fitting algorithm, under binding model 1:1 to obtain the binding response value (R) in nm.

Competition BLI assay: Various concentrations of peptides were incubated in wells with 100 nM SUMO-$^{25\text{-}109}$MDM2 protein in the kinetic buffer for 30 min. The PEG4-biotinylated p53$^{15\text{-}29}$ peptide was immobilized onto streptavidin sensors and dipped into preincubated sample wells. The association events were measured at 30 °C, 1,000 rpm. Response at equilibrium after 2 min was recorded. Based on the binding response (nm) values, the concentration of 'free' MDM2 was interpolated for each sample using the calibration curve. The free [MDM2] was denoted as [Y], and represents the amount of MDM2 available in solution to bind to the immobilized peptide ligand on the BLI sensor. Constant b is the total MDM2 concentration provided in the assay.
The dissociation constant, K_D, can be obtained from the following equation:

$$Kd = \frac{Free\ [peptide] \times Free[MDM2]}{[peptide\ protein\ complex]} = \frac{[X-(b-Y)] \times Y}{(b-Y)}$$ supplementary equation (1)

Reorganize equation (1):

$$Y^2 + (Kd + X - b) \times Y - b \times Kd = 0$$ supplementary equation (2)

Solving equation (2), we have:

$$[\mathrm{Y}] = 0.5 \times [b - \mathrm{K}_d - [\mathrm{X}] + \sqrt{([\mathrm{X}] + \mathrm{K}_d - b)^2 + 4b \times \mathrm{K}_d}\ ]$$ supplementary eqn. (3)

Non-linear regression analysis was performed in Prism 8 using the supplementary equation (3), where [Y] is the free [MDM2] in nM, and [X] is the total [peptide] in nM. K_D is the binding dissociation constant to be fitted by the curve. b is the maximal possible [MDM2] called Y_{max}, a constant to be fitted by the regression. By fitting the free [MDM2] and [peptide] to the equation, a competition binding curve and a binding constant with a standard mean error were generated by the fitting program. The free protein concentration [Y] can be obtained from the response (nm) using the calibration curve described as follows.

Calibration curve: Streptavidin sensors were soaked in blocking buffer for 5 min. After immobilizing the PEG4-biotinylated $^{15\text{-}29}$p53 peptide (100 nM of Biotin-PEG4-SQETFSDLWKLLPEN) onto streptavidin sensors, serial dilutions of SUMO-$^{25\text{-}109}$MDM2 in blocking buffer were analyzed for binding (2500 nM, 1250 nM, 625 nM, 312 nM, 156 nM, 78 nM, 50 nM, 25 nM, 13 nM, 6 nM, 3 nM, 2 nM and 1 nM). The response was recorded at equilibrium after 2 min. The concentration of stock SUMO-$^{25\text{-}109}$MDM2 was confirmed by the Bradford assay.

A curve of sensor response (nm) vs. MDM2 concentration (nM) was generated to calibrate the free MDM2 concentration in solution observed in the competition binding assay. The curve was generated using Prism 8. Approximately 50 or 100 nM of protein was subsequently used in most competition assays, as it follows the pseudo-first order kinetics that would not saturate the biosensor tip response. 250 nM of protein was used in the case of Peptide **9** as described in the following sections.

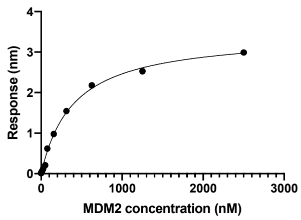

The calibration curve of SUMO-109MDM2 protein concentration when PEG4-biotinylated 15-29p53 peptide was immobilized on the streptavidin tip.

Validation of the competition BLI assay: PMI (TSFAEYWNLLSP) peptide binding validation. Approximately 50 nM SUMO-$^{25-109}$MDM2 and peptide **1** (700 nM, 350 nM, 175 nM, 88 nM, 44 nM, 22 nM, and 11 nM) were mixed together following the protocol for in solution competition assay. Free [MDM2] was estimated using the biotin-p53 peptide calibration curve and the obtained titration curve was fitted. Averaged K_D was 7.7 ± 4.5 nM over three measurements.

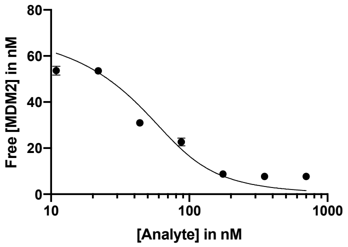

6.3 Results of competition BLI assay

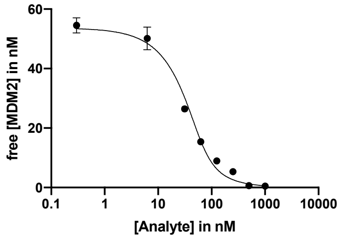

Peptide **1** binding validation. Approximately 50 nM SUMO-$^{25-109}$MDM2 and peptide **1** (1000 nM, 500 nM, 250 nM, 125 nM, 62 nM, 31 nM, 6 nM and 0.3 nM) were mixed together following the protocol for in solution competition assay. Free [MDM2] was estimated using the biotin-p53 calibration curve and the obtained titration curve was fitted. Averaged K_D was 10 ± 2.3 nM over three measurements.

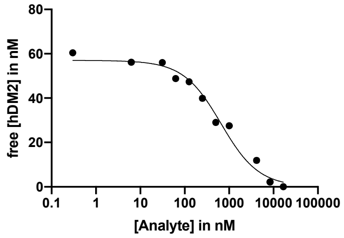

Peptide **2** binding validation. Approximately 50 nM SUMO-$^{25\text{-}109}$MDM2 and peptide **2** (16667 nM, 8333 nM, 4166 nM, 1000 nM, 500 nM, 250 nM, 125 nM, 62 nM, 31 nM, 6 nM and 0.3 nM) were mixed together following the protocol for in solution competition assay. Free [MDM2] was estimated using the biotin-p53 calibration curve and the obtained titration curve was fitted. Averaged K_D was 628 ± 89 nM over three measurements.

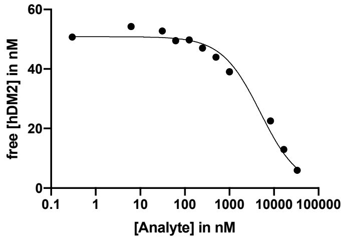

Peptide **3** binding validation. Approximately 50 nM SUMO-$^{25\text{-}109}$MDM2and peptide **3** (33333 nM, 16667nM, 8333nM, 4166 nM, 1000 nM, 500 nM, 250 nM, 125 nM, 62 nM, 31 nM, 6 nM and 0.3 nM) were mixed together following the protocol for in solution competition assay. Free [MDM2] was estimated using the biotin-p53 calibration curve and the obtained titration curve was fitted. Averaged K_D was 4912 ± 713 nM over three measurements.

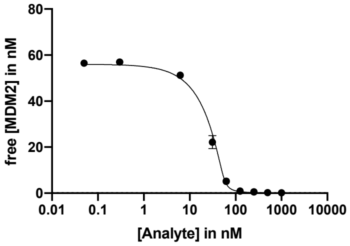

Peptide **4** binding validation. Approximately 50 nM SUMO-$^{25\text{-}109}$MDM2 and peptide **4** (1000 nM, 500 nM, 250 nM, 125 nM, 62 nM, 31 nM, 16 nM, 3 nM and 0.1 nM) were mixed together following the protocol for in solution competition assay. Free [MDM2] was estimated using the biotin-p53 calibration curve and the obtained titration curve was fitted. Averaged K_D was 0.8 ± 0.6 nM over five measurements.

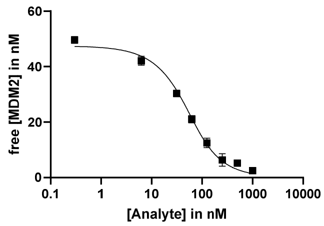

Peptide **5** binding validation. Approximately 50 nM SUMO-$^{25\text{-}109}$MDM2 and peptide **5** (1000 nM, 500 nM, 250 nM, 125 nM, 62 nM, 31 nM, 6 nM and 0.3 nM) were mixed together following the protocol for in solution competition assay. Free [MDM2] was estimated using the biotin-p53 calibration curve and the obtained titration curve was fitted. Averaged K_D was 30 ± 3.8 nM over three measurements.

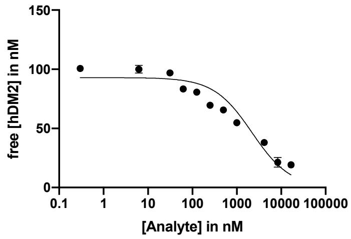

Peptide **6** binding validation. Approximately 100 nM SUMO-$^{25\text{-}109}$MDM2 and peptide **6** (16667 nM, 8133 nM, 4166 nM, 1000 nM, 500 nM, 250 nM, 125 nM, 62 nM, 31 nM and 6 nM) were mixed together following the protocol for in solution competition assay. Free [MDM2] was estimated using the biotin-p53 calibration curve and the obtained titration curve was fitted. Averaged K_D was 2144 ± 390 nM over three measurements.

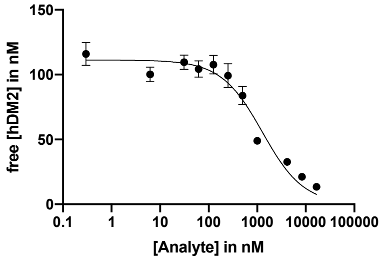

Peptide **7** binding validation. Approximately 100 nM SUMO-$^{25\text{-}109}$MDM2 and peptide **7** (16667 nM, 8133 nM, 4166 nM, 1000 nM, 500 nM, 250 nM, 125 nM, 62 nM, 31 nM, 6 nM and 0.3 nM) were mixed together following the protocol for in solution competition assay. Free [MDM2] was estimated using the biotin-p53 calibration curve and the obtained titration curve was fitted. Averaged K_D was 1224 ± 231 nM over three measurements.

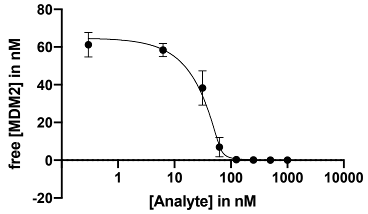

Peptide **8** binding validation. 50 nM SUMO-25-109MDM2 and peptide **8** (1000 nM, 500 nM, 250 nM, 125 nM, 62 nM, 31 nM, 6 nM, and 0.3 nM) were mixed together following the protocol for in solution competition assay. Free [MDM2] was estimated using the biotin-p53 calibration curve and the obtained titration curve was fitted. Averaged K_D was 0.7 ± 0.5 nM over three measurements.

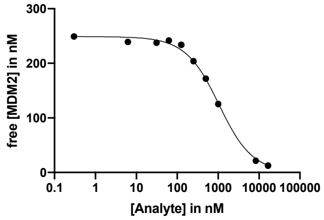

Peptide **9** binding validation. Approximately 250 nM SUMO-25-109MDM2 and peptide **9** (16667 nM, 8133 nM, 1000 nM, 500 nM, 250 nM, 125 nM, 62 nM, 31 nM, 6 nM and 0.3 nM) were mixed together following the protocol for in solution competition assay. Free [MDM2] was estimated using the biotin-p53 calibration curve and the obtained titration curve was fitted. K_D was 925 ± 70 nM (N=1).

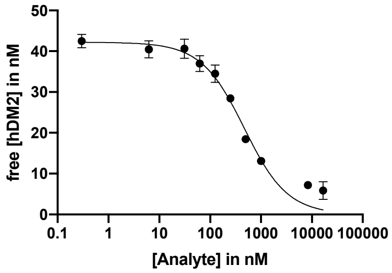

Peptide **10** binding validation. Approximately 50 nM SUMO-25-109MDM2 and peptide **10** (16667 nM, 8133 nM, 1000 nM, 500 nM, 250 nM, 125 nM, 62 nM, 31 nM, 6 nM and 0.3 nM) were mixed together following the protocol for in solution competition assay. Free [MDM2] was estimated using the biotin-p53 calibration curve and the obtained titration curve was fitted. Averaged K_D was 431 ± 46 nM over three measurements.

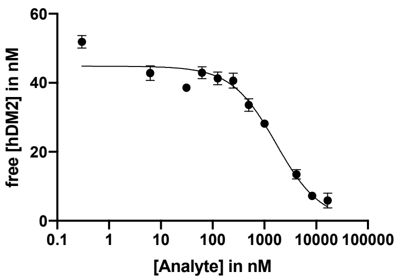

Peptide **11** binding validation. Approximately 50 nM SUMO-[25-109]MDM2 and peptide **11** (16667 nM, 8133 nM, 4166nM, 1000 nM, 500 nM, 250 nM, 125 nM, 62 nM, 31 nM, 6 nM and 0.3 nM) were mixed together following the protocol for in solution competition assay. Free [MDM2] was estimated using the biotin-p53 calibration curve and the obtained titration curve was fitted. Averaged K_D was 1588 ± 315 nM over three measurements.

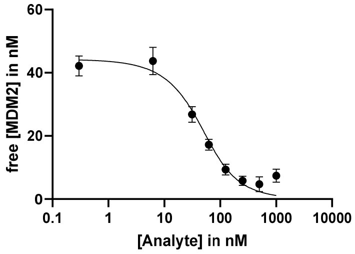

Peptide **12** binding validation. Approximately 50 nM SUMO-[25-109]MDM2 and peptide **12** (1000 nM, 500 nM, 250 nM, 125 nM, 62 nM, 31 nM, 6 nM and 0.3 nM) were mixed together following the protocol for in solution competition assay. Free [MDM2] was estimated using the biotin-p53 calibration curve and the obtained titration curve was fitted. Averaged K_D was 26 ± 5.0 nM over three measurements.

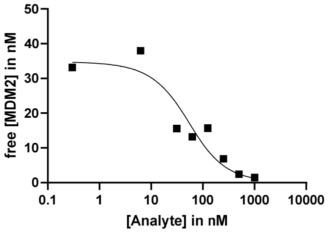

Peptide **13** binding validation. Approximately 30 nM SUMO-[25-109]MDM2 and peptide **13** (1000 nM, 500 nM, 250 nM, 125 nM, 62 nM, 31 nM, 6 nM and 0.3 nM) were mixed together following the protocol for in solution competition assay. Free [MDM2] was estimated using the biotin-p53 calibration curve and the obtained titration curve was fitted. Averaged K_D was 36 ± 8.5 nM over three measurements.

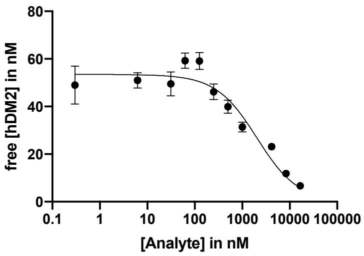

Peptide **14** binding validation. Approximately 50 nM SUMO-$^{25\text{-}109}$MDM2 and peptide **14** (16667 nM, 8133 nM, 4166nM, 1000 nM, 500 nM, 250 nM, 125 nM, 62 nM, 31 nM, 6 nM and 0.3 nM) were mixed together following the protocol for in solution competition assay. Free [MDM2] was estimated using the biotin-p53 calibration curve and the obtained titration curve was fitted. Averaged K_D was 2017 ± 514 nM over three measurements.

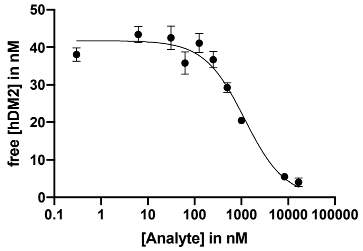

Peptide **15** binding validation. Approximately 40 nM SUMO-$^{25\text{-}109}$MDM2 and peptide **15** (16667 nM, 8133 nM, 1000 nM, 500 nM, 250 nM, 125 nM, 62 nM, 31 nM, 6 nM and 0.3 nM) were mixed together following the protocol for in solution competition assay. Free [MDM2] was estimated using the biotin-p53 calibration curve and the obtained titration curve was fitted. Averaged K_D was 1151 ± 209 nM over three measurements.

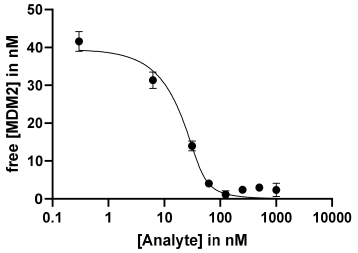

Peptide **16** binding validation. Approximately 40 nM SUMO-$^{25\text{-}109}$MDM2 and peptide **16** (1000 nM, 500 nM, 250 nM, 125 nM, 62 nM, 31 nM, 6 nM and 0.3 nM) were mixed together following the protocol for in solution competition assay. Free [MDM2] was estimated using the biotin-p53 calibration curve and the obtained titration curve was fitted. Averaged K_D was 3.0 ± 1.3 nM over three measurements.

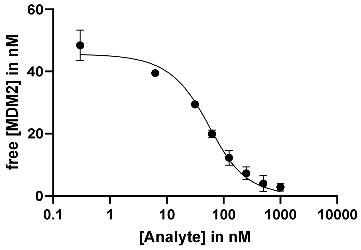

Peptide **17** binding validation. Approximately 50 nM SUMO-$^{25\text{-}109}$MDM2 and peptide **17** (1000 nM, 500 nM, 250 nM, 125 nM, 62 nM, 31 nM, 6 nM and 0.3 nM) were mixed together following the protocol for in solution competition assay. Free [MDM2] was estimated using the biotin-p53 calibration curve and the obtained titration curve was fitted. Averaged K_D was 32 ± 4.2 nM over three measurements.

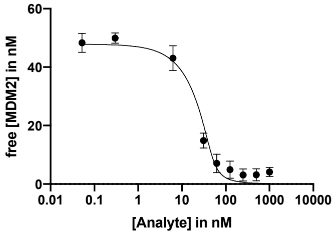

Peptide **18** binding validation. Approximately 50 nM SUMO-$^{25\text{-}109}$MDM2 and peptide **18** (1000 nM, 500 nM, 250 nM, 125 nM, 62 nM, 31 nM, 6 nM and 0.3 nM) were mixed together following the protocol for in solution competition assay. Free [MDM2] was estimated using the biotin-p53 calibration curve and the obtained titration curve was fitted. Averaged K_D was 2.7 ± 1.9 nM over three measurements.

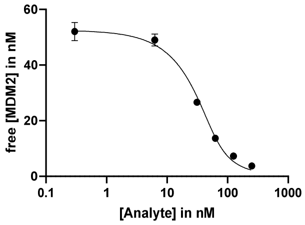

Peptide **19** binding validation. Approximately 50 nM SUMO-$^{25\text{-}109}$MDM2 and peptide **19** (250 nM, 125 nM, 62 nM, 31 nM, 6 nM and 0.3 nM) were mixed together following the protocol for in solution competition assay. Free [MDM2] was estimated using the biotin-p53 calibration curve and the obtained titration curve was fitted. Averaged K_D was 8.8 ± 1.9 nM over three measurements.

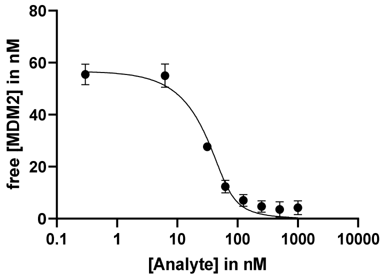

Peptide **20** binding validation. Approximately 50 nM SUMO-$^{25\text{-}109}$MDM2 and peptide **20** (1000 nM, 500 nM, 250 nM, 125 nM, 62 nM, 31 nM, 6 nM and 0.3 nM) were mixed together following the protocol for in solution competition assay. Free [MDM2] was estimated using the biotin-p53 calibration curve and the obtained titration curve was fitted. Averaged K_D was 6.2 ± 1.8 nM over three measurements.

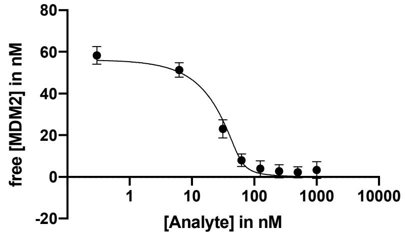

Peptide **21** binding validation. Approximately 50 nM SUMO-$^{25\text{-}109}$MDM2 and peptide **21** (1000 nM, 500 nM, 250 nM, 125 nM, 62 nM, 31 nM, 6 nM and 0.3 nM) were mixed together following the protocol for in solution competition assay. Free [MDM2] was estimated using the biotin-p53 calibration curve and the obtained titration curve was fitted. Averaged K_D was 2.1 ± 1.3 nM over three measurements.

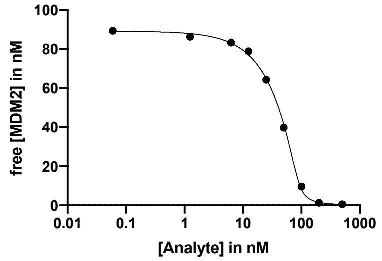

Peptide **22** binding validation. Approximately 90 nM SUMO-$^{25\text{-}109}$MDM2 and peptide **22** (500 nM, 200 nM, 100 nM, 50 nM, 25 nM, 13 nM, 6nM, 1nM and 0.06 nM) were mixed together following the protocol for in solution competition assay. Free [MDM2] was estimated using the biotin-p53 calibration curve and the obtained titration curve was fitted. K_D was 2.0 ± 1.3 nM over three measurements.

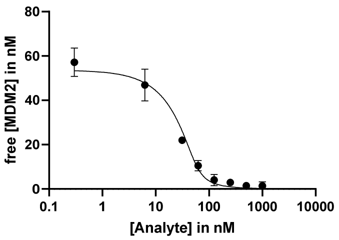

Peptide **23** binding validation. Approximately 50 nM SUMO-[25-109]MDM2 and peptide **23** (1000 nM, 500 nM, 250 nM, 125 nM, 62 nM, 31 nM, 6 nM and 0.3 nM) were mixed together following the protocol for in solution competition assay. Free [MDM2] was estimated using the biotin-p53 calibration curve and the obtained titration curve was fitted. Averaged K_D was 3.7 ± 1.5 nM over three measurements.

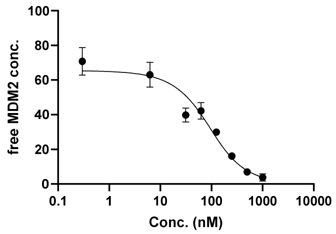

Peptide **24** binding validation. Approximately 80 nM SUMO-[25-109]MDM2 and peptide **24** (1000 nM, 500 nM, 250 nM, 125 nM, 62 nM, 31 nM, 6 nM and 0.3 nM) were mixed together following the protocol for in solution competition assay. Free [MDM2] was estimated using the biotin-p53 calibration curve and the obtained titration curve was fitted. Averaged K_D was 59 ± 11 nM over three measurements.

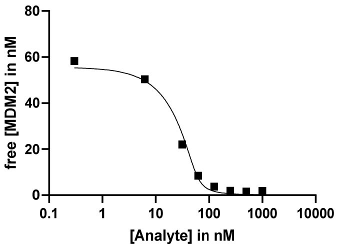

Peptide **25** binding validation. Approximately 50 nM SUMO-[25-109]MDM2 and peptide **25** (1000 nM, 500 nM, 250 nM, 125 nM, 62 nM, 31 nM, 6 nM and 0.3 nM) were mixed together following the protocol for in solution competition assay. Free [MDM2] was estimated using the biotin-p53 calibration curve and the obtained titration curve was fitted. Averaged K_D was 2.2 ± 0.8 nM over three measurements.

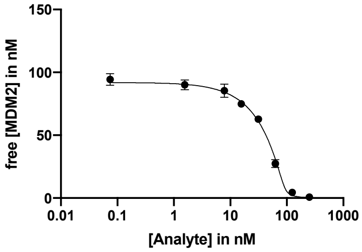

Peptide **26** binding validation. Approximately 100 nM SUMO-$^{25-109}$MDM2 and peptide **26** (250 nM, 125 nM, 62 nM, 31 nM, 16 nM, 8 nM, 2 nM and 0.1 nM) were mixed together following the protocol for in solution competition assay. Free [MDM2] was estimated using the biotin-p53 calibration curve and the obtained titration curve was fitted. Averaged K_D was 1.0 ± 0.8 nM over three measurements.

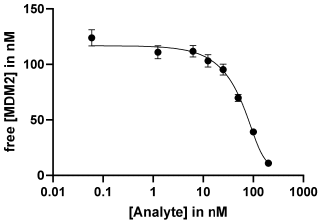

Peptide **27** binding validation. Approximately 100 nM SUMO-$^{25-109}$MDM2 and peptide **27** (1000 nM, 500 nM, 250 nM, 125 nM, 62 nM, 31 nM, 6 nM and 0.3 nM) were mixed together following the protocol for in solution competition assay. Free [MDM2] was estimated using the biotin-p53 calibration curve and the obtained titration curve was fitted. Averaged K_D was 10 ± 3.3 nM over three measurements.

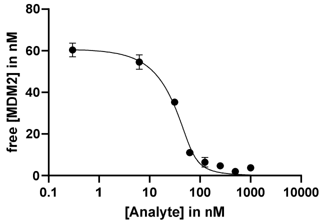

Peptide **28** binding validation. Approximately 60 nM SUMO-$^{25-109}$MDM2 and peptide **28** (1000 nM, 500 nM, 250 nM, 125 nM, 62 nM, 31 nM, 6 nM and 0.3 nM) were mixed together following the protocol for in solution competition assay. Free [MDM2] was estimated using the biotin-p53 calibration curve and the obtained titration curve was fitted. Averaged K_D was 4.7 ± 1.5 nM over three measurements.

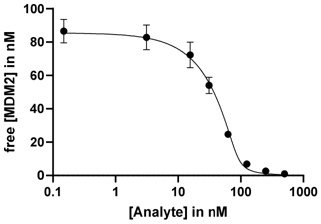

Peptide **29** binding validation. Approximately 80 nM SUMO-$^{25\text{-}109}$MDM2 and peptide **29** (500 nM, 250 nM, 125 nM, 62 nM, 31 nM, 16 nM, 3 nM and 0.1 nM) were mixed together following the protocol for in solution competition assay. Free [MDM2] was estimated using the biotin-PMI calibration curve and the obtained titration curve was fitted. Averaged K_D was 2.3 ± 1.2 nM over three measurements.

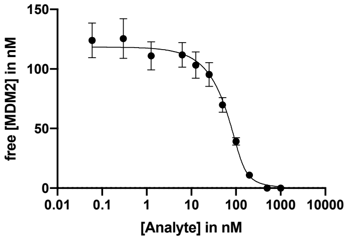

Peptide **30** binding validation. Approximately 100 nM SUMO-$^{25\text{-}109}$MDM2 and peptide **30** (500 nM, 250 nM, 125 nM, 62 nM, 31 nM, 16 nM, 3 nM and 0.1 nM) were mixed together following the protocol for in solution competition assay. Free [MDM2] was estimated using the biotin-PMI calibration curve and the obtained titration curve was fitted. Averaged K_D was 8.6 ± 2.7 nM over three measurements.

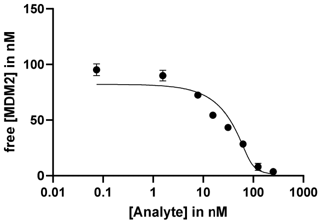

Peptide **31** binding validation. Approximately 100 nM SUMO-$^{25\text{-}109}$MDM2 and peptide **31** (500 nM, 250 nM, 125 nM, 62 nM, 31 nM, 16 nM, 3 nM and 0.1 nM) were mixed together following the protocol for in solution competition assay. Free [MDM2] was estimated using the biotin-p53 calibration curve and the obtained titration curve was fitted. Averaged K_D was 3.2 ± 2.6 nM over three measurements.

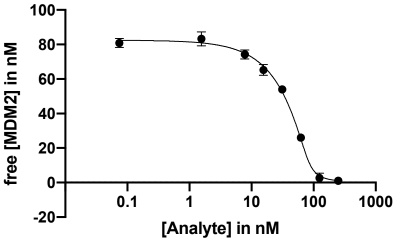

Peptide **32** binding validation. Approximately 80 nM SUMO-[25-109]MDM2 and peptide **32** (1000 nM, 500 nM, 250 nM, 125 nM, 62 nM and 31 nM) were mixed together following the protocol for in solution competition assay. Free [MDM2] was estimated using the biotin-p53 calibration curve and the obtained titration curve was fitted. Averaged K_D was 2.3 ± 0.8 nM over three measurements.

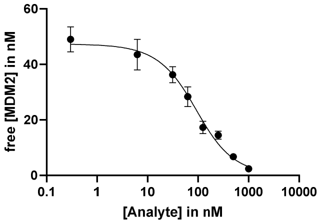

Peptide **33** binding validation. Approximately 50 nM SUMO-[25-109]MDM2 and peptide **33** (1000 nM, 500 nM, 250 nM, 125 nM, 62 nM, 31 nM, 6 nM and 0.3 nM) were mixed together following the protocol for in solution competition assay. Free [MDM2] was estimated using the biotin-p53 calibration curve and the obtained titration curve was fitted. Averaged K_D was 67 ± 14 nM over three measurements.

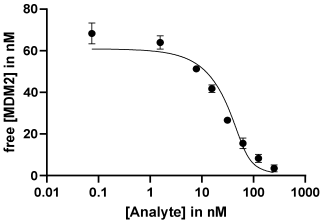

Peptide **34** binding validation. Approximately 60 nM SUMO-[25-109]MDM2 and peptide **34** (1000 nM, 500 nM, 250 nM, 125 nM, 62 nM, 31 nM, 6 nM and 0.3 nM) were mixed together following the protocol for in solution competition assay. Free [MDM2] was estimated using the biotin-p53 calibration curve and the obtained titration curve was fitted. Averaged K_D was 4.7 ± 2.4 nM over three measurements.

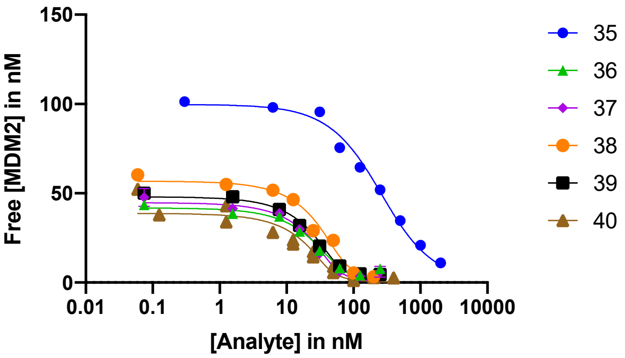

Peptide **35** binding validation. Approximately 100 nM SUMO-$^{25\text{-}109}$MDM2 and peptide **35** (2000 nM, 1000 nM, 500 nM, 250 nM, 125 nM, 62 nM, 31 nM, 6 nM and 0.3 nM) were mixed together following the protocol for in solution competition assay. Free [MDM2] was estimated using the biotin-p53 calibration curve and the obtained titration curve was fitted. Averaged K_D was 316 ± 27 nM over three measurements.

Peptide **36** binding validation. Approximately 50 nM SUMO-$^{25\text{-}109}$MDM2 and peptide **36** (250 nM, 125 nM, 62 nM, 31 nM, 6 nM and 0.3 nM) were mixed together following the protocol for in solution competition assay. Free [MDM2] was estimated using the biotin-p53 calibration curve and the obtained titration curve was fitted. Averaged K_D was 4.4 ± 1.9 nM over three measurements.

Peptide **37** binding validation. Approximately 50 nM SUMO-$^{25\text{-}109}$MDM2 and peptide **37** (250 nM, 125 nM, 62 nM, 31 nM, 15 nM, 8 nM, 1.5 nM and 0.1 nM) were mixed together following the protocol for in solution competition assay. Free [MDM2] was estimated using the biotin-p53 calibration curve and the obtained titration curve was fitted. Averaged K_D was 1.6 ± 1.3 nM over three measurements.

Peptide **38** binding validation. Approximately 50 nM SUMO-$^{25\text{-}109}$MDM2 and peptide **38** (200nM, 100 nM, 50 nM, 25 nM, 13 nM, 6.2 nM, 1.5 nM, 0.4 nM and 0.01 nM) were mixed together following the protocol for in solution competition assay. Free [MDM2] was estimated using the biotin-p53 calibration curve and the obtained titration curve was fitted. Averaged K_D was 0.4 ± 0.3 nM over three measurements.

Peptide **39** binding validation. Approximately 50 nM SUMO-$^{25\text{-}109}$MDM2 and peptide **39** (250 nM, 125 nM, 62 nM, 31 nM, 15 nM, 8 nM, 1.5 nM and 0.1 nM) were mixed together following the protocol for in solution competition assay. Free [MDM2] was estimated using the biotin-p53 calibration curve and the obtained titration curve was fitted. Averaged K_D was 3.3 ± 2.1 nM over three measurements.

Peptide **40** binding validation. Approximately 50 nM SUMO-$^{25\text{-}109}$MDM2 and peptide **40** (400 nM, 200 nM, 100 nM, 50 nM, 25 nM, 13 nM, 6.2 nM, 1.5 nM, 0.4 nM and 0.01 nM were mixed together following the protocol for in solution competition assay. Free [MDM2] was estimated using the biotin-p53 calibration curve and the obtained titration curve was fitted. Averaged K_D was 0.2 ± 0.1 nM over three measurements.

7. Supplementary References

(1) Quartararo, A. J.; Gates, Z. P.; Somsen, B. A.; Hartrampf, N.; Ye, X.; Shimada, A.; Kajihara, Y.; Ottmann, C.; Pentelute, B. L. Ultra-Large Chemical Libraries for the Discovery of High-Affinity Peptide Binders. *Nat. Commun.* **2020**, *11* (1), 3183.

(2) Rabideau, A. E.; Liao, X.; Pentelute, B. L. Delivery of Mirror Image Polypeptides into Cells. *Chem. Sci.* **2015**, *6* (1), 648–653.

(3) Li, C.; Pazgier, M.; Li, C.; Yuan, W.; Liu, M.; Wei, G.; Lu, W. Y.; Lu, W. Systematic Mutational Analysis of Peptide Inhibition of the P53-MDM2/MDMX Interactions. *J. Mol. Biol.* **2010**, *398* (2), 200–213.

(4) Pettersen, E. F.; Goddard, T. D.; Huang, C. C.; Couch, G. S.; Greenblatt, D. M.; Meng, E. C.; Ferrin, T. E. UCSF Chimera: A Visualization System for Exploratory Research and Analysis. *J. Comput. Chem.* **2004**, *25* (13), 1605–1612.

(5) Trott, O.; Olson, A. J. AutoDock Vina: Improving the Speed and Accuracy of Docking with a New Scoring Function, Efficient Optimization, and Multithreading. *J. Comput. Chem.* **2009**, NA-NA.

(6) Mijalis, A. J.; Thomas, D. A.; Simon, M. D.; Adamo, A.; Beaumont, R.; Jensen, K. F.; Pentelute, B. L. A Fully Automated Flow-Based Approach for Accelerated Peptide Synthesis. *Nat. Chem. Biol.* **2017**, *13* (5), 464–466.

(7) Wei, Y.; Thyparambil, A. A.; Latour, R. A. Protein Helical Structure Determination Using CD Spectroscopy for Solutions with Strong Background Absorbance from 190 to 230 Nm. *Biochim. Biophys. Acta - Proteins Proteomics* **2014**, *1844* (12), 2331–2337.
